# Potassium *tert*-Butoxide-Mediated Isomerization of Alkenes: A Versatile Protocol for Miscellaneous Substrates

**DOI:** 10.1021/acsomega.6c00595

**Published:** 2026-03-10

**Authors:** Héctor Mario Heras Martínez, Sydney M. Hampton, Stephen R. Isbel, Chase N. MacFarlane, Enrique B. Aparicio, Ever A. Blé-González, Alejandro Bugarin

**Affiliations:** Department of Chemistry and Physics, 3391 Florida Gulf Coast University, Fort Myers, Florida 33965, United States

## Abstract

The development of rapid and efficient routes to alkene derivatives remains a significant priority due to their broad utility as building blocks and valuable compounds. Herein, we report a simple, general, and highly efficient procedure for the (catalytic) isomerization of alkenes under mild conditions. This method is applicable to a wide range of substrates, including allylic derivatives of benzenes, aromatic and aliphatic systems, heterocycles, ethers, thioethers, amines, and sulfones. In this transformation, KO^*t*^Bu acts as both the base and the proton shuttle, enabling the reaction to proceed under air and affording quantitative yields within minutes for the majority of the substrates examined. The reaction occurs under thermodynamic control, affording the most stable isomer (typically, the *E*-alkene).

## Graphical abstract

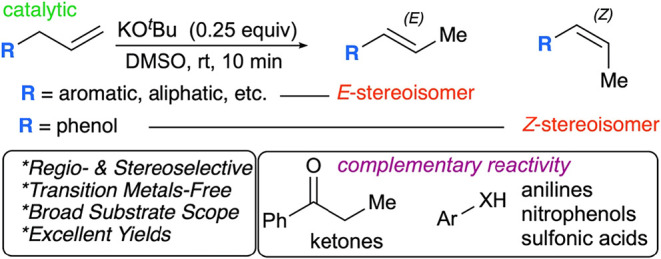

## Introduction

Alkene isomerization is a fundamental transformation in organic chemistry that involves the migration, “chain-walking,” of double bonds within a molecule.^1,2^ This seemingly simple transformation holds immense synthetic value, offering an atom-economical and straightforward approach to access diverse alkene isomers.^3^ The ability to precisely control both the position and the stereochemistry of the double bonds is crucial,^4^ as different alkene isomers exhibit distinct physical and chemical properties, making them pervasive intermediates across numerous chemical processes, particularly in the chemical industry, where alkenes are considered fine chemical feedstocks, flavor and fragrance components, building blocks, and valuable pharmaceutical intermediates.^5–8^

Alkene isomerization presents two major challenges: controlling regioselectivity and stereoselectivity.^1,9–11^ In this context, the most direct and efficient strategy for accessing internal alkenes is the isomerization of terminal alkenes, an approach that has garnered sustained attention, as it enables quick access to regio- and stereochemically defined alkenes from simple precursors (Figure 1, top). Over the past few decades, a few methods have been developed to mediate alkene isomerization that, to our knowledge, represent the best methods currently available to isomerize a broad range of alkenes (Figure 1, middle). In 2022, Morrill reported a borane-catalyzed isomerization of a myriad of terminal alkenes, albeit using a high temperature (150 °C), long reaction times (up to 2 days), and an inert atmosphere (Ar).^12^ In the same year, Cook reported the use of Ni­(NHC) and silane to perform catalytic isomerization of alkenes under slightly milder conditions (70 °C, 7 h, N_2_),^13^ while Leyva-Pérez disclosed a Ru-catalyzed (ppm loading) isomerization of miscellaneous terminal alkenes to *Z*-isomers, at elevated temperatures (150–200 °C).^14^ In 2024, Stephenson described also a ruthenium-catalyzed isomerization to *E-*alkenes under notably mild conditions (MeCN, rt, and 3 h), which in our opinion is one of the best methods to accomplish this transformation.^15^ In the same year, Luo and Zeng reported a chromium-catalyzed chain walk, also for a broad substrate scope, but requiring excess magnesium (2 equiv), to selectively move the double bond by one or two positions.^16^ Most recently, Jia et al. reported an elegant electrochemical isomerization of assorted alkenes favoring the *E* -isomer.^17^

**Figure 1:**
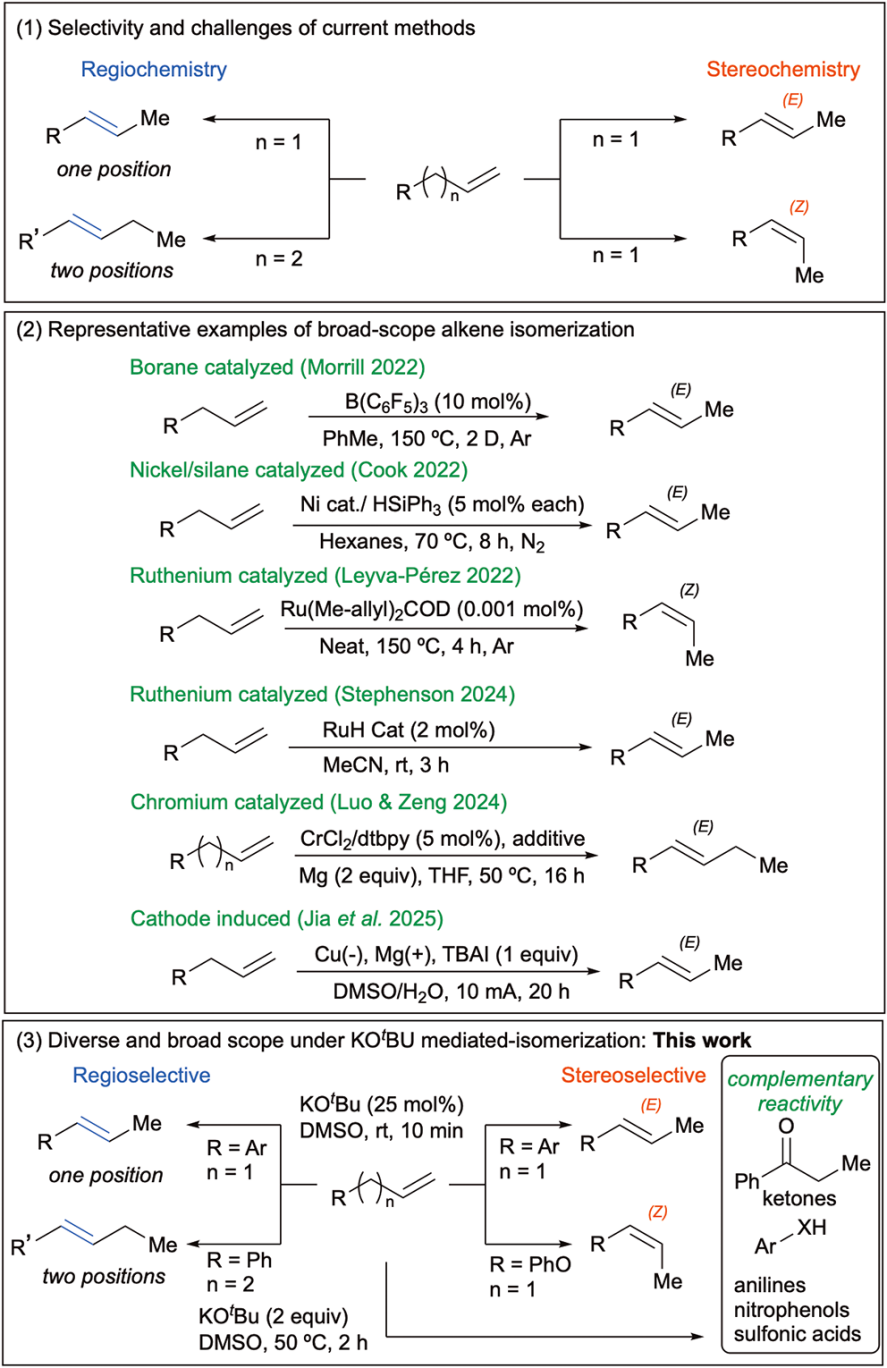
General methods for alkene isomerizations.

Beyond what we consider the best documented isomerization strategies discussed above, alkene isomerizations have been achieved using a plethora of different methods. Traditionally, the most widely used and selective protocols rely on transition-metal catalysts such as Ru,^9,18–20^ Ir,^21,22^ Co,^23–28^ Ni,^4,29,30^ Fe,^31,32^ Mo,^33^ and Pd.^34,35^ While these methods provide good control over regio- and stereoselectivity, they depend on expensive or sensitive catalysts. Alternatively, base-mediated isomerization represents a simpler and more economical option. For instance, potassium *tert*-butoxide (KO^*t*^Bu)^36^ has been known to promote allyl ether isomerization since the 1960s,^37,38^ and has been applied to isomerize allyl aryls in total synthesis. Other bases such as NaO^*t*^Bu,^39^ LDA,^40^ KOH,^41^ NaN­(SiMe_3_)_2_,^39^ crown ether-base pairs,^42^ organomagnesium clusters,^43^ and nonionic bases like proazaphosphatranes^44^ have also been employed. In some cases, these protocols tolerate air and avoid the use of transition-metal catalysts entirely, which is particularly advantageous for scale-up syntheses. As a result, alternative base-mediated methods have emerged as powerful and economical alternatives, typically operating under an ambient atmosphere and mild temperatures (25–60 °C). However, some efforts have shifted toward more sustainable methods, including electrochemical reactions, dual visible-light cobalt catalysis,^45^ and light-driven isomerization of internal to external alkenes.^46^ For example, Nicewicz and co-workers reported a visible-light-induced *E*/*Z* isomerization via single-electron oxidation of electron-rich alkenes, providing access to both stereoisomers under mild conditions.^47^ In addition, Huang recently disclosed a tandem electrochemical process that converts terminal alkenes into alcohols via isomerization to internal alkenes followed by coupling with ketones.^48^ Lastly, Hou reported a scandium-catalyzed, *Z*-selective alkene isomerization that complements existing methods favoring *Z*-alkene formation.^49^

The wide range of synthetic methods that have appeared in the literature reflects the diverse applications that alkene isomerizations can have. For example, tandem alkene isomerizations have been used in coupling reactions,^50,51^ [2 + 2][Bibr ref52] and [4 + 2][Bibr ref53] cycloadditions, rearrangements,^2^ silylations,^54^ arylations,^55^ alkylations,^56^ metathesis reactions,^57,58^ synthesis of heterocycles,^59^ unusual amino acids,^60^ acetamides, chromenes,^61^ amines,^62^ and key intermediates in total synthesis,^63,64^ among many others.^65^ Therefore, it is valid to say that isomerized alkenes are synthetically useful building blocks. However, despite the wealth of available methodologies, there remains a clear need for milder, transition-metal-free, and versatile methods capable of delivering well-defined internal alkenes across a broad substrate scope. To address this gap, we herein report an efficient alkene isomerization strategy that employs readily available starting materials (Figure 1, bottom) to furnish the desired adducts in excellent yields and short reaction times. Importantly, the reaction conditions can be easily tunable to enable access to additional compound classes, as described in the Results and Discussion, highlighting the complementary reactivity of this method for generating valuable scaffolds relevant to drug discovery and materials science, without the need for transition metals or protective atmospheres.

## Results and Discussion

The optimization of our protocol was initiated from an observation made while studying a direct allylic halogenation of allylbenzene (**1a**) in the presence of potassium *tert-*butoxide (KO^*t*^Bu) in DMSO-*d*_6_. It was observed that 1.1 equiv of KO^*t*^Bu promoted full conversion of **1a** to a new adduct (initially difficult to identify due to deuterium incorporation). However, after a few more experiments, we were able to identify the adduct as **2a**. To our delight, this alkene isomerization was completed really fast (minutes) and at room temperature (∼22 °C). To validate this, we first run the experiment in DMSO, where no reaction was observed in the absence of KO^*t*^Bu (Table 1, entry 1). However, the addition of KO^*t*^Bu (0.25 equiv) catalyzed the reaction affording the isomerization adduct **2a** in quantitative yield after only 10 min (entry 2). Other quantities of KO^*t*^Bu either did not complete the reaction (<0.25 equiv) or form the product as expected (>0.5 equiv) (not shown). Both DMSO and DMF proved to be the best solvents (entries 2 and 16, >99%), followed by MeCN (23%, entry 17) and ethanol (3%, entry 18); other nonpolar or protic solvents were ineffective. The bases Et_3_N, ^*i*^Pr_2_EtN, and pyridine alsopromoted the reaction, but gave yields lower than KO^*t*^Bu. DMAP (entry 14), DBU (entry 15), and carbonate bases (entries 6–10) were inefficient bases. High yields were observed with NaO^*t*^Bu and LiO^*t*^Bu (98% and 91%, respectively), while KOH afforded the adduct in 97% yield, albeit using 0.5 equiv (entry 5). Furthermore, the reaction proceeded in quantitative yield under both dark and inert atmosphere, indicating that neither light nor oxygen affects the reaction (entries 19 and 20). Therefore, KO^*t*^Bu was chosen as the base and DMSO as the solvent to investigate the substrate scope.

**Table 1:**
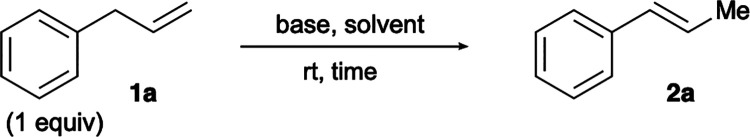
Optimization of Reaction Conditions^a^

entry	time (min)	base (0.25 equiv)	solvent	yield (%)^b^
1	120	none	DMSO	NR
2	10	KO^*t*^Bu	DMSO	>99
3	10	NaO^*t*^Bu	DMSO	98
4	10	LiO^*t*^Bu	DMSO	91
5	120	KOH^c^	DMSO	97
6	120	Li_2_CO_3_	DMSO	NR
7	120	Na_2_CO_3_	DMSO	NR
8	120	K_2_CO_3_	DMSO	NR
9	120	Cs_2_CO_3_	DMSO	NR
10	120	CaCO_3_	DMSO	NR
11	120	Et_3_N	DMSO	10
12	120	*i*-Pr_2_EtN	DMSO	5
13	120	Pyridine	DMSO	traces
14	120	DMAP	DMSO	NR
15	120	DBU	DMSO	NR
16	10	KO^*t*^Bu	DMF	99
17	120	KO^*t*^Bu	MeCN	23
18	120	KO^*t*^Bu	Ethanol	3
19	10	KO^*t*^Bu	DMSO	>99^d^
20	10	KO^*t*^Bu	DMSO	>99^e^

a Reactions were carried out with allylbenzene **1a** (0.4 mmol, 53 μL, 1 equiv), base (0.1 mmol, 0.25 equiv), in 1.0 mL of DMSO at room temperature (∼22 °C). Then, the mixture was stirred for 10 min, analyzed by ^1^H NMR using DMS0-*d*_6_ as the solvent, and mesitylene as the internal standard. Those unreacted after 10 min were analyzed again at 2 h.

b Isolated yields using silica gel flash chromatography.

c 0.5 equiv of KOH was used.

d Dark.

e Argon atmosphere. NR = no reaction.

With the optimal conditions established, the substrate scope was assessed (Table 2). Diverse allylbenzenes were well tolerated under the standard reaction conditions, affording the corresponding adducts in excellent yields with high *E*-stereoselectivity. The parent allylbenzene furnished its styrene adduct in 95% yield. Both electron-withdrawing (**2c**, CF_3_, 97%) and electron-donating substituents (**2e**, OMe, 93%) were tolerated. *Para*-substituted derivatives (**2b**, Me and **2d**, F) were obtained in 94 and 90%, respectively. *Ortho*-substituted moieties provided the desired products (**2f**, OMe and **2g**, Br) in 98 and 95%, respectively. Similarly, *Meta-*substituted allylbenzenes (**2h**, methyl eugenol and **2i**, safrole) gave excellent yields of 98 and 95%, respectively. The most sterically hindered substrate, 2-allylmesitylene, afforded its respective product **2j** in 93% yield but with a 2:1 *E*/*Z* ratio, presumably due to steric effects from the two *ortho* substituents. Finally, 1-allylnaphthalene proved to be an excellent substrate, affording **2k** in 99% yield. It is worth noting that all allylic starting materials exhibited similar *R*_f_ values to their respective styrene products; therefore, all of the reactions were run to full conversion (10 min) to ensure complete product formation. The slightly lower isolated yields reflect product loss during purification by silica gel flash chromatography; otherwise, the yields could have been reported as quantitative for all substrates.

**Table 2:**
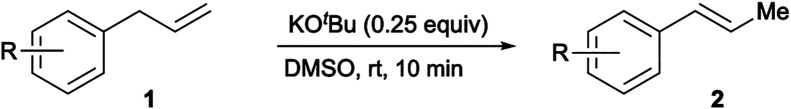

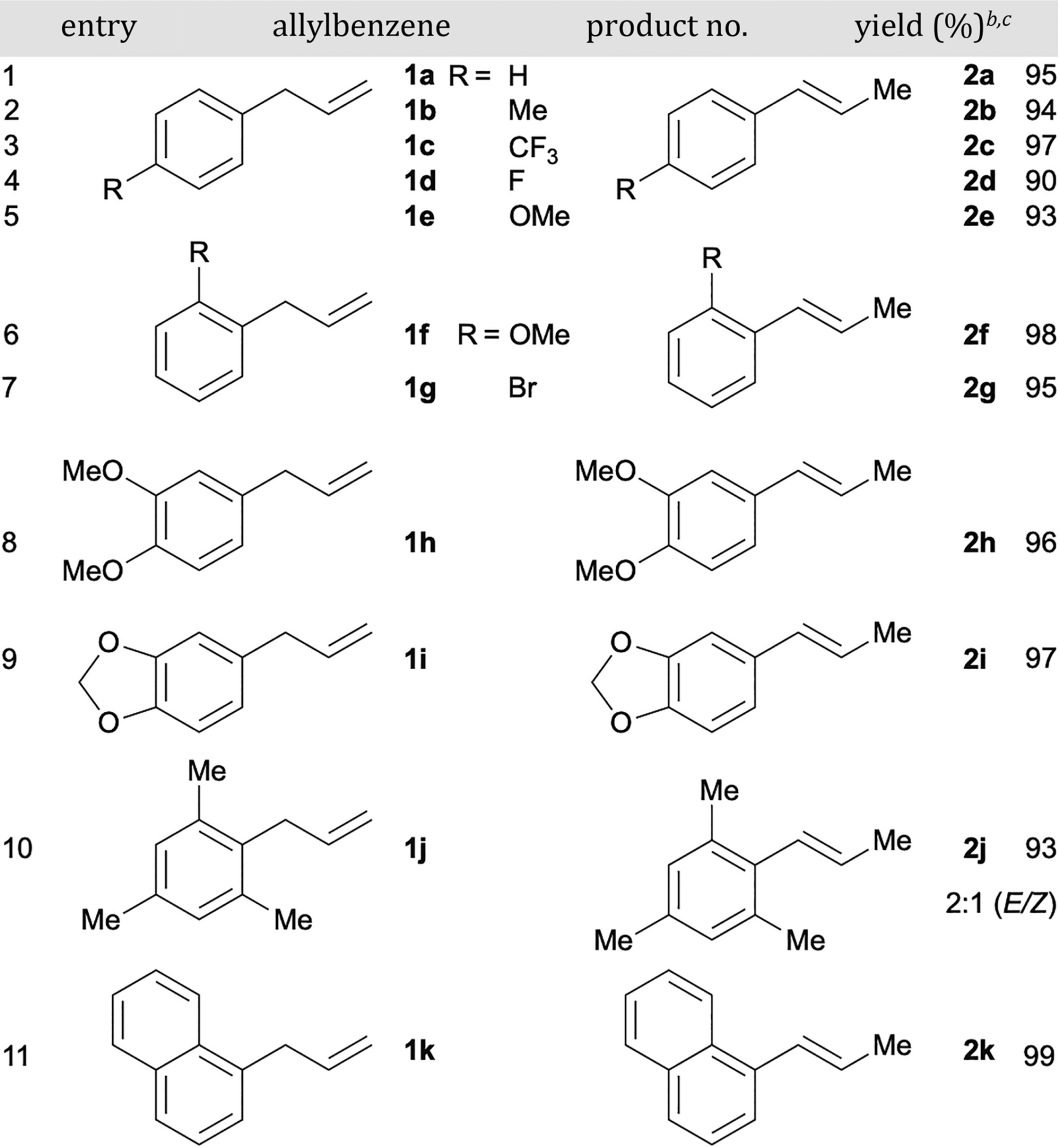
Scope of Allylbenzene Derivatives^a^

a Reactions were carried out with allylbenzene derivative **1a**–**k** (0.4 mmol, 1 equiv), KO^*t*^Bu (0.1 mmol, 0.25 equiv), in 1.0 mL of DMSO at room temperature (∼22 °C). Then, the mixture was stirred for 10 min in open air.

b Isolated yields based on **1**, using silica gel flash chromatography.

c *E*/*Z* > 20/1 unless noted in parentheses.

After the successful production of miscellaneous *trans*-β-methylstyrene derivatives **2** (Table 2), the scope of special allyl substrates was evaluated (Table 3). First, (2-methylpropenyl)­benzene **1l** was reacted under the standard reaction conditions and, to our delight, having an extra substitution on the alkene did not diminish the reactivity, affording adduct **2l** in 92% yield. Then, 3-allylpyridine **1m** was subjected to the same reaction conditions to produce its adduct **2m** in 80% yield. Encouraged by this result, another heterocycle, 1*N*-allylimidazole **1n**, was reacted and the adduct **2n** was obtained in 91% yield and 6:1 *E*/Z ratio.^66^ Next, other allylic systems were investigated; first allylthiother **1o** was studied affording **2o** in 92% but in 1:1 *E*/Z ratio. Furthermore, allyl ether **1p** was studied affording **2p** in 97%, but this time only the *Z* stereoisomer was observed, matching prior reported studies.^67–70^ To further confirm this difference in stereoselectivity, two adducts containing both the allylbenzene and the allyl ethers were reacted, and as expected, adducts **2q** and **2r** were obtained in very high yields (93% and 96%) and excellent stereoselectivity, *E* for the styrene and *Z* for the vinyl ethers moieties, again confirming the trend.

**Table 3:**
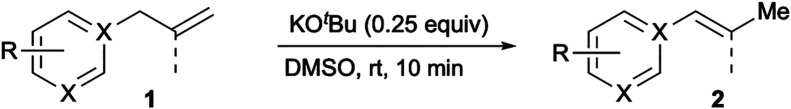

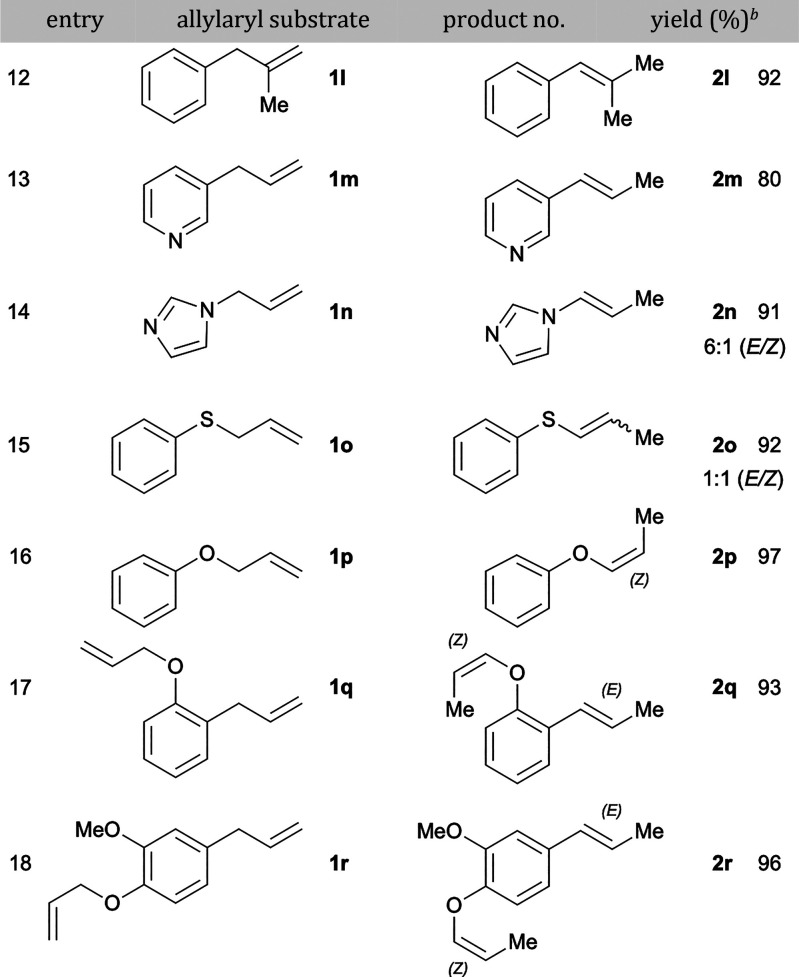
Scope of Special Allylic Derivatives^a^

a Reactions were carried out with allyl derivative **1l**–**r** (0.4 mmol, 1 equiv), KO^*t*^Bu (0.1 mmol, 0.25 equiv), in 1.0 mL of DMSO at room temperature (∼22 °C). Then, the mixture was stirred for 10 min in open air.

b Isolated yields based on **1**, using silica gel flash chromatography.

c *E*/*Z* > 20/1 unless noted in parentheses.

The scope of less or nonactivated alkenes was also evaluated (Table 4). Under the standard reaction conditions, these substrates failed to undergo the isomerization. However, modification of the initial conditions proved to be effective. For example, 4-phenylbutene **1s** was fully converted to *trans*-β-ethylstyrene **2s** using 1 equiv of KO^*t*^Bu and stirred for 2 h at 50 °C. However, since **2s** is a volatile compound, only 65% yield was isolated.^71^ With the modified conditions, 1-hexene produced a mixture of the expected adducts along with multiple inseparable byproducts. At 110 °C, limonene **1u** furnished cymene in 10%; although the yield is low, this result demonstrates the versatility of our method, as it promotes not only isomerization but also aromatization in the presence of air.^72^ Then at 80 °C, 1,5-cyclooctadiene **1v** was efficiently converted to conjugated 1,3-cycloactadiene **2v** in 99% yield. Interestingly, vinyl cyclohexane **1w** was unreactive under the modified conditions, whereas allyl cyclohexane **1x** decomposed. Lastly, 4-methyl-1-pentene **1y** also presented a mixture of adducts, consistent with those previously observed for 1-hexene.

**Table 4:**
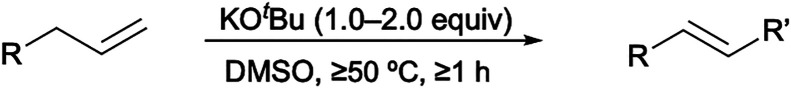

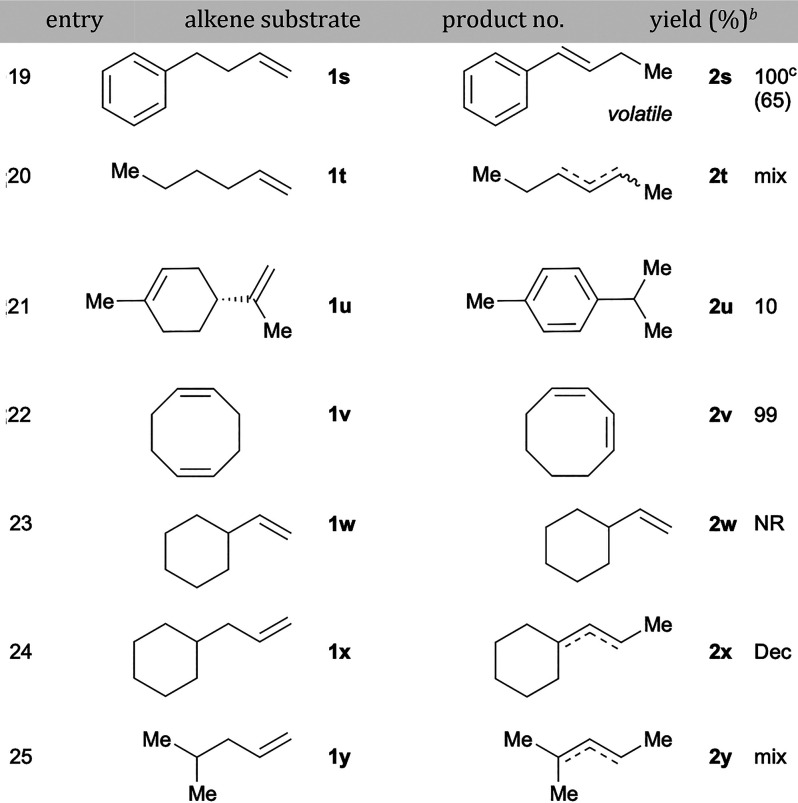
Scope of Alkene Derivatives^a^

a Reactions were carried out with alkene **1s**–**y** (0.4 mmol, 1 equiv), KO^*t*^Bu (0.8 mmol, 2.0 equiv), in 1.0 mL of DMSO at room temperature (50–110 °C). Then, the mixture was stirred for 2 h in open air.

b Isolated yields based on **1**, using silica gel flash chromatography.

c 100% conversion, but the volatility of **2s** diminished the isolated yield to 65%. Dec = decomposed.

Allylphenols and alcohols were also investigated (Table 5). Initially, 2-allyllphenol **1z** was subjected to modified conditions, 2 equiv of KO^*t*^Bu and stirred for 10 min at 50 °C, where 1 equiv of KO^*t*^Bu was used to quench the acidic proton. To our satisfaction, adduct **2z** was obtained in 85% yield, albeit with a 9:1 *E*/*Z* ratio. The acetylated version **1aa** was also reacted and as expected, the acetyl group was removed to give **2z** in 84% yield, with the same 9:1 *E*/*Z* ratio. Then, eugenol was subjected to the same reaction conditions furnishing its adduct **2ab** in 83% yield and 2.5:1 *E*/*Z* ratio. Then, 1-phenylprop-2-en-1-ol **1ac** was reacted to furnish ketone **2ac** in 98% yield via isomerization followed by tautomerization. To accomplish both reactions, this substrate had to be stirred at 80 °C for 2 h employing 2 equiv of KO^*t*^Bu. Similarly, 1-phenylbut-3-en-1-ol **1ad** furnishes ketone **2ad** in 96% yield. Finally, distinct reactivity was observed for the special substrates. For instance, 4-nitro allylphenol **1ae** was unreactive under the standard conditions (Table 1). However, upon increasing the base loading to 2 equiv and stirring the reaction at 50 °C for 30 min, deallylation occurred producing 4-nitrophenol **2ae** in 88% yield, consistent with literature precedents for deallylation of allylic nitrophenols.^73–75^ A similar outcome was observed with *N*-allyl aniline **1af**, which produced aniline **2af** in quantitative yield, but some mass was lost during purification to give 57% isolated yield. Last but not least, allyl sulfones **1ag** and **1ah** were efficiently deallylated even under the standard reaction conditions (Table 1), forming benzenesulfonic acid **2ag** and tolylsulfonic acid **2ah**, in 95% and 96% yield, respectively (Table 5).

**Table 5:**
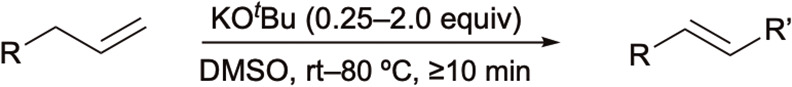

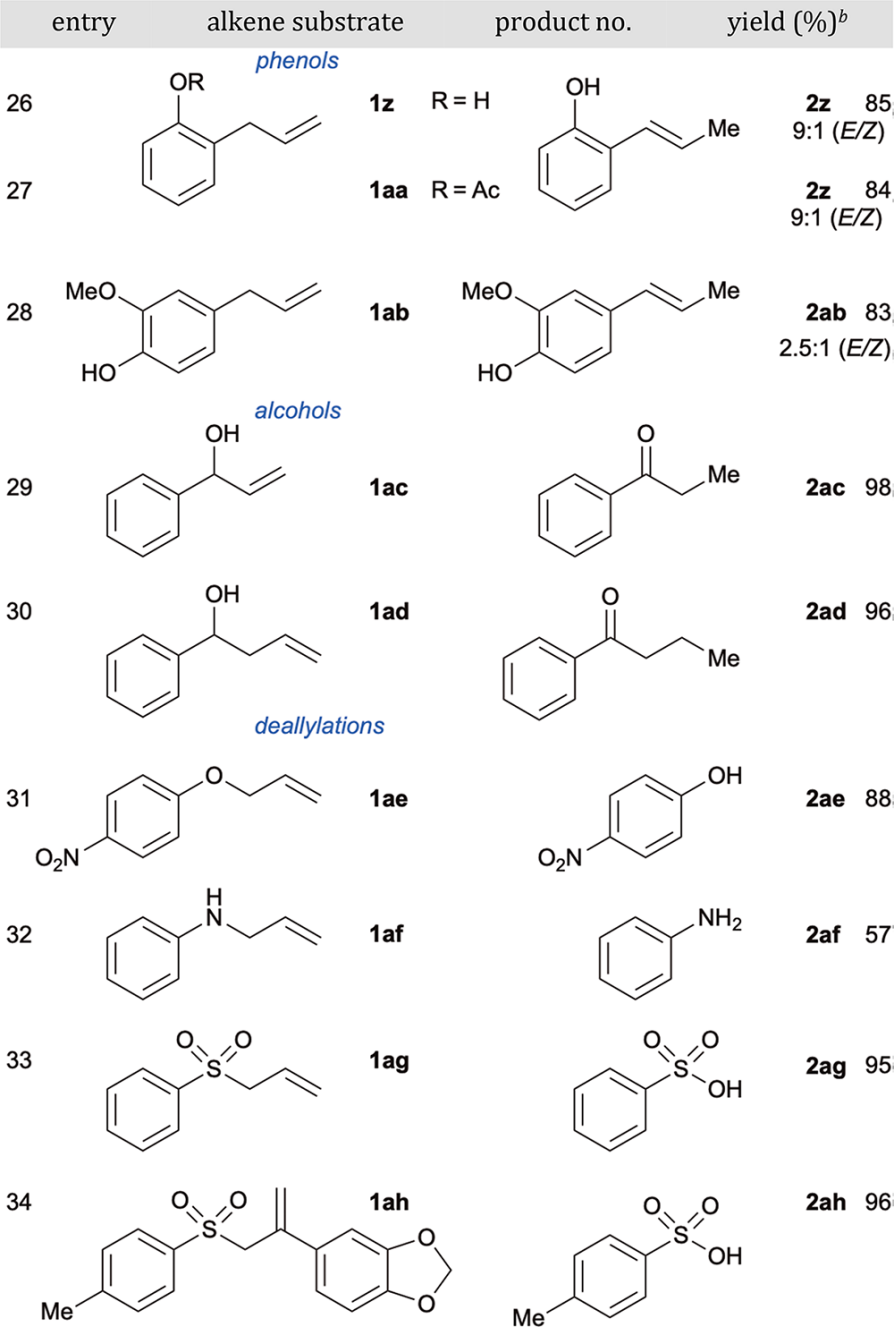
Scope of Allyl Phenols, Alcohols, and Derivatives^a^

a Reactions were carried out with alkene **1z–1ah** (0.4 mmol, 1 equiv), KO^*t*^Bu (0.1–0.8 mmol, 0.25–2.0 equiv), in 1.0 mL of DMSO at ∼22–80 °C. Then, the mixture was stirred for 10–120 min in open air.

b Isolated yields based on **1**, using silica gel flash chromatography.

The mechanism of alkene isomerization depends on the nature of the substrate and reaction conditions, but in general, base-mediated isomerization of allylbenzenes proceeds through an E1cb-like mechanism.^76,77^ This process involves deprotonation at the allylic position, followed by protonation at a different site, resulting in the migration of the double bond. Since our reaction conditions favor thermodynamic control,^78^ the more stable *E*-alkene was observed. An exception was observed for allyl phenyl ether **1p**, where the *Z*-alkene was exclusively obtained, presumably due to the oxygen’s lone pair interaction with the developing carbanion and/or the potassium ion, which stabilizes the transition state conformation during the protonation step.^38^ This observation is consistent with the literature precedents. Notably, proton shuttle was observed when allylbenzene was reacted in DMSO-*d6*, with deuterium incorporation at different positions depending on base equivalents and temperature (not shown).^30,79,80^ In contrast, the reaction of allyl phenyl ether in DMSO-*d6* yielded exclusively the *Z*-isomer, with no detectable deuterium incorporation, further supporting a distinct mechanism and reactivity, potentially involving the formation of a dimsyl anion acting as an electron donor–acceptor complex with benzene, as previously observed by Laulhé during his light-promoted cross-coupling reactions.^81^ Additionally, *N*-allyl aniline **1af** displayed reactivity similar to that of 4-phenylbutene **1s**, undergoing isomerization over two positions to form an imine (observed by crude ^1^H NMR), which then hydrolyzed to deliver deallylated aniline **2af**. On the basis of these observations, additional studies are provided in SI (Tables S3–S7) to further confirm the power of the divergent reactivity patterns and highlight the role of KO^*t*^Bu in DMSO.

## Conclusion

This article reports a straightforward isomerization of miscellaneous alkenes. The method is applicable for a wide range of substrates, including allylic derivatives of benzenes, aromatic and aliphatic systems, heterocycles, ethers, thioethers, amines, and sulfones, while dienes^82^ are also expected to be compatible. When the standard reaction conditions were unproductive, increasing the amount of KO^*t*^Bu, temperature, or reaction time, the adducts were successfully obtained. Furthermore, this methodology provided more than 30 adducts from commercial starting material, all potential building blocks in the synthesis of valuable materials. Therefore, this versatile procedure could be utilized for a myriad of substrates and diverse molecular targets, including ketones and deallylation strategies.

## Supplementary Material



## Notes

Detailed experimental procedures and copies of ^1^H NMR and ^13^C NMR spectra for all of the compounds (PDF)

## †

Department of Forensic Science, Sam Houston State University, Huntsville, TX 77340, United States

## ‡

Department of Chemistry, Florida International University, Miami, FL, 33199, United States

## §

División Académica de Ciencias Básicas, Universidad Juárez Autónoma de Tabasco, Carretera Cunduacán-Jalpa Km. 0.5, Cunduacán 86690, Mexico
