# Supplementary material for: Potassium tert-Butoxide-Mediated Isomerization of Alkenes: A Versatile Protocol for Miscellaneous Substrates
Source: ACS Omega. 2026 Mar 10;11(11):18419–26. doi: 10.1021/acsomega.6c00595 (PMC13019183; doi:10.1021/acsomega.6c00595)
Supplement: Supplementary file 1 [file ao6c00595_si_001.pdf]

Potassium *tert*-Butoxide-Mediated Isomerization of Alkenes: A Versatile Protocol for Miscellaneous Substrates

Héctor Mario Heras Martínez, Sydney M. Hampton, Stephen R. Isbel, Chase N. MacFarlane, Enrique B. Aparicio, Ever A. Blé-González, and Alejandro Bugarin*

** Department of Chemistry and Physics, Florida Gulf Coast University,*
10501 FGCU Boulevard South, Fort Myers, FL 33965
abugarin@fgcu.edu

Table of Contents

General Information…………………………………………………………… S-1

References to Starting Materials……………………………………………… S-2

References to products………………………………………………………… S-3

Protocols for Preparation and Characterization Data………………………… S-4

^{1}H and ^{13}C NMR Spectra ………………………………………………… S-11

Extended optimization studies for selected substates………………………… S-40

1. General Information

All reactions were carried out under air in oven-dried glassware with magnetic stirring at room temperature. Commercially available reagents and solvents were used as received without further purification. Both ACS-grade DMSO and extra-dry DMSO (99.7%) provided comparable results. Purification of reaction products was carried out by flash column chromatography using silica gel 60 (230-400 mesh). TLC visualization was accompanied with UV light. Concentration in vacuo refers to the removal of volatile solvent using a rotary evaporator attached to a dry diaphragm pump (10-15 mm Hg) followed by pumping to a constant weight with an oil pump (<300 mTorr).

^{1}H NMR spectra were recorded at 400 MHz and are reported relative to $CDCl_3$ (δ = 7.26 ppm) or DMSO-d_6 (δ = 2.50 ppm). ^{1}H NMR coupling constants (J) are reported in Hertz (Hz) and multiplicities are indicated as follows: s (singlet), d (doublet), t (triplet), m (multiplet). Proton-decoupled ^{13}C NMR spectra were recorded at 100 MHz and reported relative to $CDCl_3$ (δ = 77.16 ppm) or DMSO-d_6 (δ = 39.50). IR experiments were recorded with neat samples on a Jasco FT/IR-4700 fitted with diamond ATR sample plate. GCMS data was recorded on a Shimadzu GC-2010 plus System (GCMS-QP2010 SE).

2. References to Starting Materials

Table S1: Allylbenzene and Alkenes derivatives

Compound No.	Alkene	Ref.	Compound No.	Alkene	Ref.	Compound No.	Alkene	Ref.
**1a**	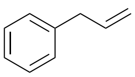	**S1**	**1l**	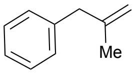	**S1**	**1x**	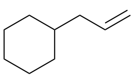	**S1**
**1b**	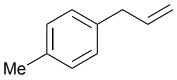	**S1**	**1m**	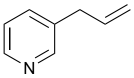	**S4**	**1y**	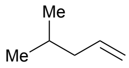	**S1**
**1c**	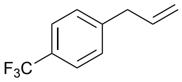	**S2**	**1n**	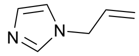	**S1**	**1z**	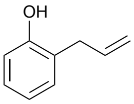	**S7**
**1d**	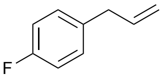	**S2**	**1o**	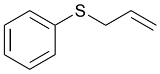	**S1**	**1aa**	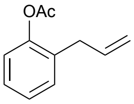	**S2**
**1e**	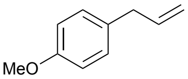	**S3**	**1p**	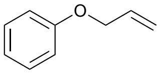	**S5**	**1ab**	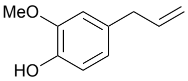	**S2**
**1f**	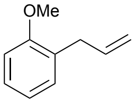	**S2**	**1q**	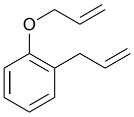	**S6**	**1ac**	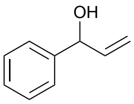	**S2**
**1g**	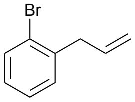	**S2**	**1r**	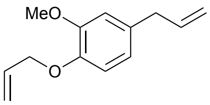	**S6**	**1ad**	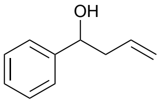	**S2**
**1h**	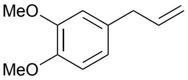	**S2**	**1s**	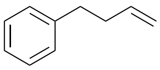	**S1**	**1ae**	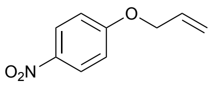	**S2**
**1i**	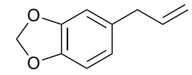	**S1**	**1t**	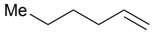	**S1**	**1af**	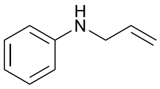	**S1**
**1j**	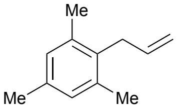	**S1**	**1u**	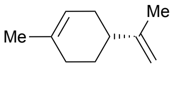	**S1**	**1ag**	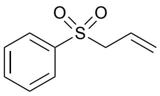	**S1**
**1k**	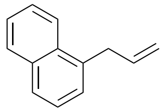	**S1**	**1v**	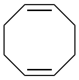	**S1**	**1ah**	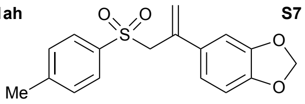	**S7**
			**1w**	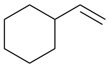	**S1**			

S1) Commercially available (Fisher Scientific), S2) Commercially available (Millipore Sigma), S3) Commercially available (Across Organics), S4) Commercially available (Enamine), S5) Commercially available (Oakwood chemical), S6) The phenol starting material was commercially available (Fisher Scientific), which was easily allylated using allyl bromide, K_2CO_3, in acetone. S7) Obtained from in house project; *New J. Chem*. **2023**, *47*, 17020-17025.

3. **Table S2:** List and References to the Isomerized Products

Compound No.	Alkene	Ref.	Compound No.	Alkene	Ref.	Compound No.	Alkene	Ref.
**2a**	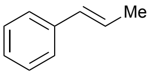	**S8**	**2l**	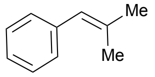	**S10**	**2x**	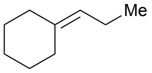	**S8**
**2b**	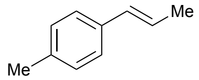	**S8**	**2m**	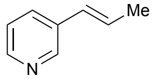	**S8**	**2y**	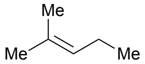	**S9**
**2c**	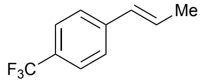	**S9**	**2n**	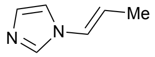	**S13**	**2z**	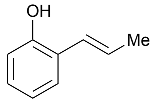	**S12**
**2d**	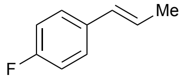	**S8**	**2o**	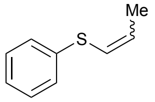	**S14**	**2aa**	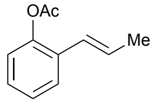	**S12**
**2e**	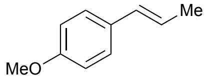	**S8**	**2p**	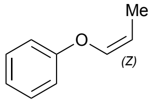	**S15**	**2ab**	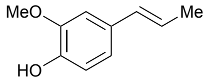	**S12**
**2f**	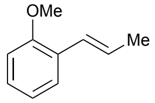	**S10**	**2q**	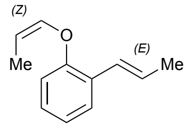	**S12**	**2ac**	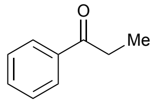	**S19**
**2g**	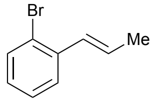	**S11**	**2r**	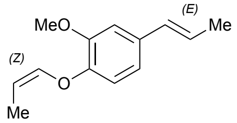	**S16**	**2ad**	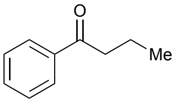	**S19**
**2h**	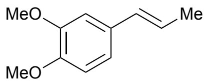	**S8**	**2s**	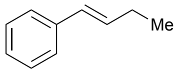	**S9**	**2ae**	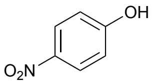	**S20**
**2i**	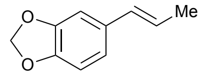	**S12**	**2t**	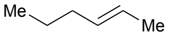	**S17**	**2af**	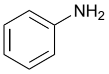	**S11**
**2j**	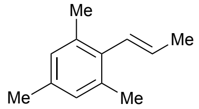	**S9**	**2u**	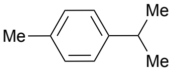	**S18**	**2ag**	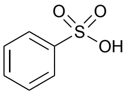	**S21**
**2k**	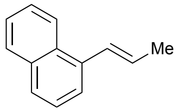	**S9**	**2v**	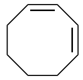	**S8**	**2ah**	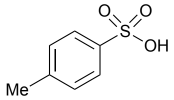	**S22**
			**2w**	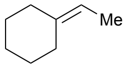	**S8**			

(S8) Mayer, M.; Welther, A.; Jacobi von Wangelin, A. *ChemCatChem* **2011**, *3* (10), 1567–1571.
(S9) Kawamura, K. E.; Chang, A. S. M.; Smith, H. M.; Morris, P. T.; Cook, A. K. *Organometallics* **2022**, *41* (4), 486–496.
(S10) Hogan, A. M. L.; Tricotet, T.; Meek, A.; Khokhar, S. S.; O'Shea, D. F. *J. Org. Chem.* **2008**, *73* (15), 6041–6044.
(S11) Davies, A. M.; Greene, K. H.; Allen, A. R.; Szymczak, N. K.; Stephenson, C. R. J. *J. Org. Chem.* **2024**, *89*, 13, 9647
(S12) Perdriau, S.; Chang, M. C.; Otten, E.; Heeres, H. J.; De Vries, J. G. *Chem. Eur. J.***2014**, *20* (47), 15434–15442.
(S13) Motoyama, Y.; Abe, M.; Kamo, K.; Kosako, Y.; Nagashima, H. *Chem. Commun.* **2008**, *42*, 5321–5323.
(S14) Yu, Z.; Yan, S.; Zhang, G.; He, W.; Wang, L.; Li, Y.; Zeng, F. *Adv. Synth. Catal.* **2006**, *348* (1–2), 111–117.
(S15) Shi, M.; Wang, L.; Chen, Q.; He, M.; Shen, M.; Zhang, Z. H. *Tetrahedron Lett.* **2020**, *61* (36), 152278.
(S16) Sanz-Navarro, S.; Mon, M.; Doménech-Carbó, A.; Greco, R.; et al. *Nat. Commun.* **2022**, *13* (1), 2831.
(S17) Witt, T.; Häußler, M.; Kulpa, S.; Mecking, S. *Angew. Chem.* **2017**, *129* (26), 7697–7702.
(S18) Cherney, A. H.; Hedley, S. J.; Mennen, S. M.; Tedrow, J. S. *Organometallics* **2019**, *38* (1), 97–102.
(S19) Mameda, N.; Peraka, S.; Marri, M. R.; Kodumuri, S.; Chevella, D.; Gutta, N. *Appl. Catal. A Gen.* **2015**, *505*, 213–216.
(S20) Shukla, A.; Singha, R. K.; Sengupta, M.; Sasaki, T.; Pendem, C.; Bal, R. *ChemistrySelect* **2018**, *3* (4), 1129–1141.
(S21) Wu, Y.; Wu, L. *Monatsh. Chem.* **2017**, *148* (4), 675–682.
(S22) Parida, K. N.; Chandra, A.; Moorthy, J. N. *ChemistrySelect* **2016**, *1* (3), 490–494.

4. General Procedures and Characterization Data

Procedure A:

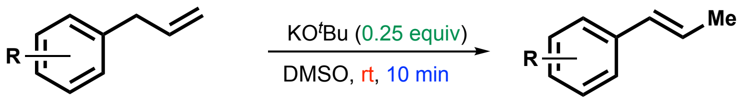

To a 5 mL round-bottom flask equipped with a magnetic stir bar were added the olefin substrate (0.40 mmol, 1.0 equiv.), KO^{t}Bu (11.22 mg, 0.1 mmol, 0.25 equiv.), and the solvent DMSO (1 mL) at room temperature. The reaction mixture was stirred open to air for 10 minutes. For purification, the crude mixture was directly subjected to a flash column chromatography on silica gel using gradient of Hexanes/EtOAc (from 1:0 to 4:1) to afford the pure product. *Note*: Using 1% v/v of Et_3N to neutralize the silica gel during purification resulted in cleaner isolated adducts.

Note: Lower catalyst loadings (e.g., 5 mol%) has been shown to be effective at higher temperatures (50–80 °C). However, for some substrates, these conditions may result in incomplete conversion, formation of side products, incomplete reactions, and altered stereoselectivity.

Procedure B:

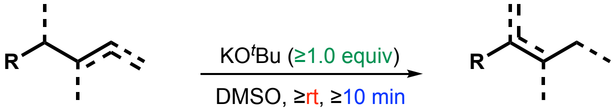

To a 5 mL round-bottom flask equipped with a magnetic stir bar were added the alkene substrate (0.40 mmol, 1.0 equiv.), KO^{t}Bu (89.76 mg, 0.8 mmol, 2 equiv. or otherwise as noted below), and DMSO (1 mL). The reaction mixture was stirred open to air and heated at 50 °C (or higher as noted below) for 10 min or until completion. The crude product was purified by flash column chromatography on silica gel using 4:1 mixtures Hexanes/EtOAc to afford the pure product (adding 1% v/v of Et_3N is recommended). *Note*: Refer below for specific base equivalents, temperature, and reaction time.

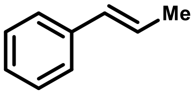

(*E*)-prop-1-en-1-ylbenzene CAS No. 873-66-5 (**2a**).
Prepared from allylbenzene (47 mg, 0.4 mmol) according to procedure **A**. Colorless liquid (45 mg, 95%) as inseparable mixture of isomers with 56:1 (*E/Z*) ratio.
IR (neat, cm^{-1}): 3022, 2920, 1593, 1500, 980.
^{1}H NMR (400 MHz, $CDCl_3$) δ 7.33 (dt, J = 15.2, 7.4 Hz, 3H), 7.26 – 7.21 (m, 1H), 6.43 (dq, J = 15.6, 1.7 Hz, 1H), 6.27 (dq, J = 15.7, 6.5 Hz, 1H), 1.91 (dd, J = 6.5, 1.7 Hz, 3H).
^{13}C NMR (101 MHz, $CDCl_3$) δ 138.03, 131.11, 128.60, 126.86, 125.93, 125.83, 18.65.
LRMS (EI) Calcd for C_9H_{10} [M^+] *m/z*: 118.08 Found: 118 (M).
The spectroscopic data agrees with those reported in the literature (S8)

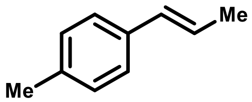

(*E*)-1-methyl-4-(prop-1-en-1-yl)benzene CAS No. 2698-14-8 (**2b**).
Prepared from 1-allyl-4-methylbenzene (52 mg, 0.4 mmol) according to procedure **A**. Colorless liquid (49 mg, 94%) as inseparable mixture of isomers with 60:1 (*E/Z*) ratio.
IR (neat, cm^{-1}): 3022, 2920, 1593, 1500, 1295, 1249, 1141, 980.
^{1}H NMR (400 MHz, $CDCl_3$) δ 7.25 (d, J = 7.8 Hz, 2H), 7.12 (d, J = 6.3 Hz, 2H), 6.39 (d, J = 15.8 Hz, 1H), 6.25 – 6.16 (m, 1H), 2.35 (s, 3H), 1.89 (d, J = 6.6 Hz, 3H).
^{13}C NMR (101 MHz, $CDCl_3$) δ 136.52, 135.26, 130.93, 129.29, 125.82, 124.76, 21.26, 18.62.
LRMS (EI) Calcd for $C_{10}H_{12}$ [M^+] *m/z*: 132.09 Found: 132 (M).
The spectroscopic data agrees with those reported in the literature (S8)

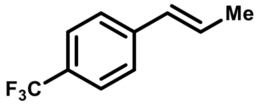

(*E*)-1-(prop-1-en-1-yl)-4-(trifluoromethyl)benzene CAS No. 42006-43-9 (**2c**).
Prepared from 1-allyl-4-(trifluoromethyl)benzene (74 mg, 0.4 mmol) according to procedure **A**. Colorless liquid (72 mg, 97%).
IR (neat, cm^{-1}): 3028, 2918, 1918, 1614, 1321, 852

^{1}H NMR (400 MHz, $CDCl_3$) δ 7.52 (d, J = 8.2 Hz, 4H), 7.40 (d, J = 8.1 Hz, 4H), 6.46 – 6.35 (m, 3H), 6.35 – 6.27 (m, 1H), 2.04 (s, 1H), 1.90 (dd, J = 1.2, 6.2 Hz, 6H).
^{13}C NMR (101 MHz, $CDCl_3$) δ 141.01, 129.54, 128.31, 126.25, 125.15, 18.26.
LRMS (EI) Calcd for $C_{13}H_{16}O_2$ [M^+] *m/z*: 186.18 Found: 186(M).
The spectroscopic data agrees with those reported in the literature (S9)

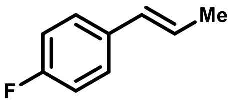

(*E*)-1-fluoro-4-(prop-1-en-1-yl)benzene CAS No. 100921-53-7 (**2d**).
Prepared from 1-allyl-4-fluorobenzene (54 mg, 0.4 mmol) according to procedure **A**. Colorless liquid (49 mg, 90%).
^{1}H NMR (400 MHz, $CDCl_3$) δ 7.53 (d, *J* = 8.2 Hz, 2H), 7.41 (d, *J* = 8.1 Hz, 2H), 6.43 (d, *J* = 17.3 Hz, 1H), 6.39 – 6.30 (m, 1H), 1.91 (d, *J* = 4.8 Hz, 3H).
^{13}C NMR (101 MHz, $CDCl_3$) δ 163.16, 160.72, 134.19, 134.16, 129.94, 127.34, 127.27, 125.53, 125.52, 115.52, 115.31, 18.55.
LRMS (EI) Calcd for $C_9H_9F_3$ [M^+] *m/z*: 136.17 Found: 136 (M).
The spectroscopic data agrees with those reported in the literature (S8).

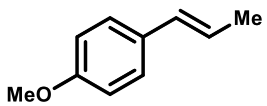

(*E*)-1-methoxy-4-(prop-1-en-1-yl)benzene CAS No. 4180-23-8 (**2e**).
Prepared from 1-allyl-4-methoxybenzene (59 mg, 0.4 mmol) following procedure **A**. Colorless liquid (55 mg, 93%) as inseparable mixture of isomers with 23:1 (*E/Z*) ratio.
^{1}H NMR (400 MHz, $CDCl_3$) δ 7.27 (d, *J* = 8.7 Hz, 2H), 6.84 (d, *J* = 8.7 Hz, 2H), 6.35 (dd, *J* = 15.7, 1.8 Hz, 1H), 6.10 (dq, *J* = 15.7, 6.6 Hz, 1H), 3.80 (s, 3H), 1.86 (dd, *J* = 6.6, 1.7 Hz, 3H).
^{13}C NMR (101 MHz, $CDCl_3$) δ 158.65, 130.90, 130.41, 126.99, 123.63, 114.00, 55.40, 18.59.
LRMS (EI) Calcd for $C_{10}H_{12}O$ [M^+] *m/z*: 148.18 Found: 148 (M).
The spectroscopic data agrees with those reported in the literature (S8).

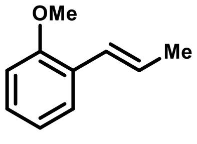

(*E*)-1-methoxy-2-(prop-1-en-1-yl)benzene CAS No. 2077-36-3 (**2f**). Prepared from 1-allyl-2-methoxybenzene (59 mg, 0.4 mmol) according to procedure **A**. Colorless liquid (58 mg, 98%).
^{1}H NMR (400 MHz, $CDCl_3$) δ 7.43 (d, *J* = 7.7 Hz, 1H), 7.21 (t, *J* = 7.8 Hz, 1H), 6.94 (t, *J* = 7.5 Hz, 1H), 6.88 (d, *J* = 8.2 Hz, 1H), 6.76 (d, *J* = 15.8 Hz, 1H), 6.30 – 6.21 (m, 1H), 3.86 (s, 3H), 1.93 (d, *J* = 6.7 Hz, 3H).
^{13}C NMR (101 MHz, $CDCl_3$) δ 156.25, 127.86, 127.12, 126.68, 126.53, 125.69, 120.74, 110.77, 55.51, 19.10.
LRMS (EI) Calcd for $C_{10}H_{12}O$ [M^+] *m/z*: 148.09 Found: 148(M).
The spectroscopic data agrees with those reported in the literature (S10).

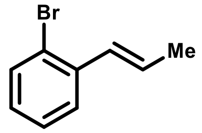

(*E*)-1-bromo-2-(prop-1-en-1-yl)benzene CAS No. 70968-46-6 (**2g**).
Prepared from 1-allyl-2-bromobenzene (79 mg, 0.4 mmol) according to procedure **A**. Colorless liquid (75 mg, 95%) as inseparable mixture of isomers with 20:1 (*E/Z*) ratio.
^{1}H NMR (400 MHz, $CDCl_3$) δ 7.52 (dt, *J* = 8.0, 1.3 Hz, 1H), 7.47 (dd, *J* = 7.7, 1.7 Hz, 1H), 7.24 (t, *J* = 7.6 Hz, 1H), 7.06 (t, *J* = 7.7 Hz, 1H), 6.74 (d, *J* = 15.6 Hz, 1H), 6.24 – 6.15 (m, 1H), 1.94 (dt, *J* = 6.7, 1.4 Hz, 3H).
^{13}C NMR (101 MHz, $CDCl_3$) δ 137.77, 132.91, 129.97, 129.02, 128.21, 127.51, 126.91, 123.09, 18.84.
LRMS (EI) Calcd for C_9H_9Br [M^+] *m/z*: 195.99 Found: 196 (M) 198 (M+2).
The spectroscopic data agrees with those reported in the literature (S11).

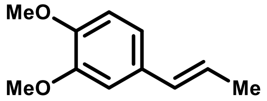

(*E*)-1,2-dimethoxy-4-(prop-1-en-1-yl)benzene CAS No. 6379-72-2 (**2h**).
Prepared from 4-allyl-1,2-dimethoxybenzene (71 mg, 0.4 mmol) according to procedure **A**. Colorless liquid (68 mg, 96%) as inseparable mixture of isomers with 56:1 (*E/Z*) ratio.
^{1}H NMR (400 MHz, $CDCl_3$) δ 6.89 (d, *J* = 2.0 Hz, 1H), 6.85 (dd, *J* = 8.2, 2.0 Hz, 1H), 6.79 (d, *J* = 8.2 Hz, 1H), 6.33 (dd, *J* = 15.7, 1.8 Hz, 1H), 6.10 (dq, *J* = 15.7, 6.6 Hz, 1H), 3.89 (s, 3H), 3.87 (s, 3H), 1.86 (dd, *J* = 6.6, 1.7 Hz, 3H).
^{13}C NMR (101 MHz, $CDCl_3$) δ 149.02, 148.20, 131.21, 130.67, 123.94, 118.74, 111.18, 108.42, 56.01, 55.86, 18.53.
LRMS (EI) Calcd for $C_{11}H_{14}O_2$ [M^+] *m/z*: 178.23 Found: 178(M).
The spectroscopic data agrees with those reported in the literature (S8).

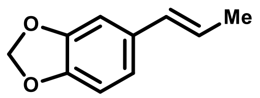

(*E*)-5-(prop-1-en-1-yl)benzo[*d*][1,3]dioxole CAS No. 4043-71-4 (**2i**).
Prepared from 5-allylbenzo[*d*][1,3]dioxole (64 mg, 0.4 mmol) according to procedure **A**. Colorless liquid (62 mg, 97%).
^{1}H NMR (400 MHz, $CDCl_3$) δ 6.89 (s, 1H), 6.74 (s, 2H), 6.32 (dq, *J* = 15.6, 1.7 Hz, 1H), 6.07 (dq, *J* = 15.7, 6.6 Hz, 1H), 5.93 (s, 2H), 1.86 (dd, *J* = 6.6, 1.7 Hz, 3H).
^{13}C NMR (101 MHz, $CDCl_3$) δ 148.01, 146.59, 132.58, 130.63, 124.06, 120.18, 108.32, 105.42, 101.02, 18.49.
LRMS (EI) Calcd for $C_{10}H_{10}O_2$ [M^+] *m/z*: 162.19 Found: 162(M).
The spectroscopic data agrees with those reported in the literature (S12).

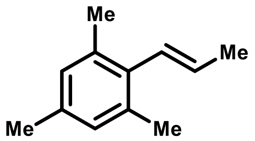

(*E*)-1,3,5-trimethyl-2-(prop-1-en-1-yl)benzene CAS No. 2077-41-0 (**2j**).
Prepared from 2-allyl-1,3,5-trimethylbenzene (64 mg, 0.4 mmol) according to procedure **A**. Colorless liquid (60 mg, 93%) as inseparable mixture of isomers with 2:1 (*E/Z*) ratio.
IR (neat, cm^{-1}): 3003, 2915, 1999, 1549, 850.
^{1}H NMR (400 MHz, $CDCl_3$) *Major* (*E*): δ 6.89 (s, 2H), 6.36 (d, *J* = 11.5 Hz, 1H), 5.74 – 5.65 (m, 1H), 2.30 (s, 9H), 1.94 (dd, *J* = 1.8, 6.5 Hz, 3H,); *Minor* (*Z*): δ 6.90 (s, 2H), 6.33 (m, 1H), 5.89 – 5.81 (m, 1H), 2.27 (s, 3H), 2.20 (s, 6H), 1.50 (dd, *J* = 1.8, 6.9 Hz, 3H).
^{13}C NMR (101 MHz, $CDCl_3$) *Major* (*E*) δ 136.0, 135.69, 134.88, 130.18, 128.51, 127.91, 21.02, 20.29, 19.00. *Minor* (*Z*): δ 136.14, 135.95, 133.68, 128.47, 128.44, 127.42, 21.02, 20.29, 19.00, 14.44.
LRMS (EI) Calcd for $C_{12}H_{16}$ [M^+] *m/z*: 160.26 Found: 160(M).
The spectroscopic data agrees with those reported in the literature (S9).

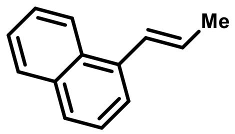

(*E*)-1-(prop-1-en-1-yl)naphthalene CAS No. 53269-01-5 (**2k**).
Prepared from 1-allylnaphthalene (67 mg, 0.4 mmol) according to procedure **A**. Colorless liquid (67 mg, 99%) as inseparable mixture of isomers with 24:1 (*E/Z*) ratio.
^{1}H NMR (400 MHz, $CDCl_3$) δ 8.18 (ddd, *J* = 8.8, 5.1, 2.4 Hz, 1H), 7.88 (dp, *J* = 7.2, 1.9 Hz, 1H), 7.78 (dd, *J* = 8.3, 3.4 Hz, 1H), 7.59 – 7.45 (m, 4H), 7.18 (dq, *J* = 15.5, 2.0 Hz, 1H), 6.29 (ddt, *J* = 12.7, 6.8, 2.2 Hz, 1H), 2.04 (ddd, *J* = 6.8, 3.5, 1.5 Hz, 3H).
^{13}C NMR (101 MHz, $CDCl_3$) δ 135.85, 133.71, 131.18, 129.10, 128.57, 128.31, 127.31, 125.89, 125.81, 125.74, 124.08, 123.59, 19.14.
LRMS (EI) Calcd for $C_{13}H_{12}$ [M^+] *m/z*: 168.24 Found: 168(M).
The spectroscopic data agrees with those reported in the literature (S9).

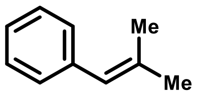

(2-methylprop-1-en-1-yl)benzene CAS No. 768-49-0 (**2l**).
Prepared from (2-methylallyl)benzene (52 mg, 0.4 mmol) according to procedure **A**. Colorless liquid (48 mg, 92%).
^{1}H NMR (400 MHz, $CDCl_3$) δ 7.33 (t, *J* = 7.8 Hz, 2H), 7.24 (d, *J* = 8.1 Hz, 2H), 7.20 (t, *J* = 7.3 Hz, 1H), 6.29 (s, 1H), 1.93 (s, 3H), 188 (s, 3H).
^{13}C NMR (101 MHz, $CDCl_3$) δ 138.80, 135.63, 128.85, 128.15, 125.89, 125.21, 27.03, 19.52.
LRMS (EI) Calcd for $C_{10}H_{12}$ [M^+] *m/z*: 132.09 Found: 132(M).
The spectroscopic data agrees with those reported in the literature (S10).

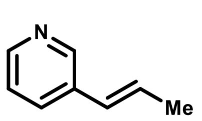

***(E)*-3-(prop-1-en-1-yl)pyridine CAS No. 15376-62-2 (2m)**.
Prepared from 3-Allylpyridine (47 mg, 0.4 mmol) according to procedure A. Colorless liquid (38 mg, 80%).
^{1}H NMR (400 MHz, $CDCl_3$) δ 8.51 (s, 1H), 8.38 (s, 1H), 7.65 – 7.57 (m, 1H), 7.18 (dd, J = 4.5, 7.8 Hz, 1H), 6.38 – 6.21 (m, 2H), 1.91 – 1.84 (m, 3H).
^{13}C NMR (101 MHz, $CDCl_3$) δ 147.27, 133.47, 132.48, 128.42, 127.32, 123.61, 18.29.
LRMS (EI) Calcd for C_8H_9N [M^+] *m/z*: 119.07 Found: 119(M).
The spectroscopic data agrees with those reported in the literature (S8).

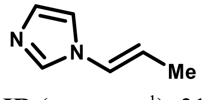

(*E*)-1-(prop-1-en-1-yl)-1H-imidazole CAS No. 157370-55-3 (**2n**).
Prepared from 1-allylimidazole (43 mg, 0.4 mmol) according to procedure **A**. Colorless liquid (39.1 mg, 91%) as inseparable mixture of isomers with 6:1 (*E/Z*) ratio.
IR (neat, cm^{-1}): 3114, 2921, 1678, 1495, 656.

^{1}**H NMR** (400 MHz, $CDCl_3$) *Major* (*E*): δ 7.56 (s, 1H), 7.05 (d, J = 11.1 Hz, 2H), 6.72 – 6.64 (m, 1H), 5.81 (dq, J = 6.8, 13.8 Hz, 1H), 1.80 (dd, J = 1.8, 6.9 Hz, 3H). *Minor* (*Z*): δ 7.25 (s, 1H), 7.08 (d, J = 1.6 Hz, 1H), 7.00 (s, 1H), 6.60 (dq, J = 1.9, 8.7 Hz, 1H), 5.58 – 5.45 (m, 1H), 1.75 (dd, J = 1.73, 6.9 Hz, 3H).
^{13}**C NMR** (101 MHz, $CDCl_3$) *Major* (*E*): δ 136.01, 130.15, 124.42, 115.50, 15.21. *Minor* (*Z*): δ 135.83, 129.97, 129.48, 120.20, 116.62, 12.92.
LRMS (EI) Calcd for $C_6H_8N_2$ [M^+] *m/z*: 108.14 Found: 108(M).
The spectroscopic data agrees with those reported in the literature (S13).

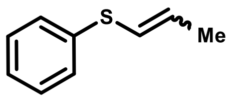

(*E/Z*)-phenyl(prop-1-en-1-yl)sulfane CAS No. 22103-05-5 (**2o**).
Prepared from allylphenyl sulfide (60.0 mg, 0.4 mmol) according to procedure **A**. Colorless liquid (57.1 mg, 95%) as inseparable mixture of isomers with 1:1 (*E/Z*) ratio.
IR (neat, cm^{-1}): 3057, 2911, 1719, 1583, 736.
^{1}**H NMR** (400 MHz, $CDCl_3$) (*E*)-isomer: δ 7.36 – 7.13 (m, 4H), 7.20 – 7.18 (m, 1H), 6.25 – 6.18 (m, 1H), 6.04 – 5.94 (m, 1H), 1.86 – 1.84 (m, 3H). (*Z*)-isomer*:* δ 7.38 – 7.23 (m, 4H), 7.20 – 7.18 (m, 1H), 6.17 – 6.13 (dq, J = 1.4, 14.8 Hz, 1H), 5.90 – 5.86 (dq, J = 6.8, 9.2 Hz, 1H), 1.83 (d, J = 2.4 Hz, 3H).
^{13}**C NMR** (*E*,*Z*) (101 MHz, $CDCl_3$) δ 136.56, 132.54, 128.89, 128.70, 127.93, 126.07, 123.50, 121.57, 18.68 (*E*), 136.31, 14.83 (*Z*).
LRMS (EI) Calcd for $C_9H_{10}S$ [M^+] *m/z*: 150.05 Found: 150(M).
The spectroscopic data agrees with those reported in the literature (S14).

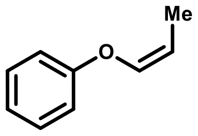

(*Z*)-(prop-1-en-1-yloxy)benzene CAS No. 4696-24-6 (**2p**).
Prepared from allyl phenyl ether (59.28 mg, 0.4 mmol) according to procedure **A**. Colorless liquid (58.09 mg, 98%).
^{1}**H NMR** (400 MHz, $CDCl_3$) δ 7.35 – 7.26 (m, 2H), 7.07 – 6.96 (m, 3H), 6.38 (dq, J = 1.8, 6.1 Hz, 1H), 5.01 – 4.80 (m, 1H), 1.72 (dd, J = 1.7, 6.9 Hz, 3H).
^{13}**C NMR** (101 MHz, $CDCl_3$) δ 157.17, 140.50, 129.21, 121.99, 115.81, 107.16, 9.05.
LRMS (EI) Calcd for $C_9H_{10}O$ [M^+] *m/z*: 134.18 Found: 134(M).
The spectroscopic data agrees with those reported in the literature (S15).

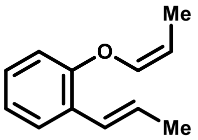

1-[(*E*)-prop-1-en-1-yl]-2-([(*Z*)-prop-1-en-1-yl]oxy)benzene CAS No. 1229611-01-1 (**2q**).
Prepared from 1-allyl-2-(allyloxy)benzene (69 mg, 0.4 mmol) according to procedure **A**. Colorless liquid (64 mg, 93%).
^{1}**H NMR** (400 MHz, $CDCl_3$) δ 7.46 (dd, *J* = 7.7, 1.7 Hz, 1H), 7.18 (td, *J* = 7.7, 1.7 Hz, 1H), 7.01 (td, *J* = 7.5, 1.2 Hz, 1H), 6.93 (dd, *J* = 8.1, 1.2 Hz, 1H), 6.76 (dd, *J* = 15.9, 1.9 Hz, 1H), 6.36 (dt, *J* = 6.0, 1.7 Hz, 1H), 6.30 (dt, *J* = 15.8, 6.6 Hz, 1H), 4.94 – 4.87 (m, 1H), 1.94 (dd, *J* = 6.7, 1.8 Hz, 3H), 1.78 (dd, *J* = 6.9, 1.7 Hz, 3H).
^{13}**C NMR** (101 MHz, $CDCl_3$) δ 154.25, 141.53, 127.89, 127.79, 127.08, 126.53, 125.21, 122.67, 115.52, 107.26, 19.11, 9.59, 9.57.
LRMS (EI) Calcd for $C_{12}H_{14}O$ [M], 174.11 Found: 174(M).
The spectroscopic data agrees with those reported in the literature (S12).

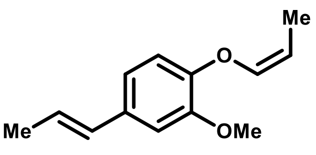

2-methoxy-4-((*E*)-prop-1-en-1-yl)-1-(((*Z*)-prop-1-en-1-yl)oxy)benzene CAS No. 2823403-83-2 (**2r**).
Prepared from 4-allyl-1-(allyloxy)-2-methoxybenzene (81 mg, 0.4 mmol) according to procedure **A**. Colorless liquid (78 mg, 96%). Inseparable mixture of isomers with 25:1 (*E/Z*) ratio (styrene).
^{1}**H NMR** (400 MHz, $CDCl_3$) δ 6.92 (d, *J* = 1.9 Hz, 1H), 6.87 (d, *J* = 8.2 Hz, 1H), 6.85 (dd, *J* = 8.2, 1.9 Hz, 1H), 6.32 (dd, *J* = 15.7, 1.7 Hz, 1H), 6.27 (dd, *J* = 6.0, 1.8 Hz, 1H), 6.13 (dq, *J* = 15.7, 6.6 Hz, 1H), 4.92 – 4.85 (m, 1H), 3.89 (s, 3H), 1.86 (dd, *J* = 6.6, 1.7 Hz, 3H), 1.73 (dd, *J* = 6.9, 1.6 Hz, 3H).
^{13}**C NMR** (101 MHz, $CDCl_3$) δ 149.64, 146.10, 141.88, 133.34, 130.56, 124.78, 118.64, 116.54, 109.60, 107.67, 56.11, 18.54, 9.60.
LRMS (EI) Calcd for $C_{11}H_{14}O_2$ [M^+] *m/z*: 204.27 Found: 204(M).
The spectroscopic data agrees with those reported in the literature (S16).

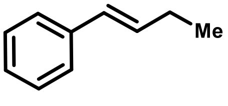

(*E*)-but-1-en-1-ylbenzene CAS No. 1005-64-7 (**2s**).
Prepared from 4-phenyl-1-butene (52.88 mg, 0.4 mmol) according to procedure **B** at 50 °C with 1 equivalent of KO^{t}Bu and stirred for 2 h. Colorless and volatile liquid (100% by ^{1}H NMR using mesitylene as internal standard, and 34.3 mg, 65% isolated yield).
^{1}H NMR (400 MHz, CDCl3) δ 7.49 – 7.26 (m, 5H), 7.20 – 7.16 (m, 1H), 6.37 (dt, J = 1.4, 15.7 Hz, 1H), 6.26 (dt, J = 6.3, 15.8 Hz, 1H), 2.26 – 2.19 (m, 2H), 1.09 (t, J = 7.5 Hz, 3H).
^{13}C NMR (101 MHz, CDCl3) δ: 138.03, 132.76, 128.59, 128.32, 126.86, 126.01, 26.19, 13.77.
LRMS (EI) Calcd for $C_{10}H_{12}$ [M$^+$] *m/z*: 132.09 Found: 132(M).
The spectroscopic data agrees with those reported in the literature (S9).

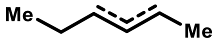

2-hexene or **3-hexene** CAS No. 592-43-8 (**2t**).
Aimed from 1-hexene (47 mg, 0.4 mmol) according to procedure **B** for 2 h.
Try 1: rt & 2 equiv. of base = no reaction. Try 2: 50 °C & 2 equiv. of base = no reaction. Try 3: 50 °C & 3 equiv. of base = no reaction. Try 4: 80 °C & 2 equiv. of base = mixture of products. Crude ^{1}H NMR analysis showed signals consistent with one- and two-position alkene isomerization. The starting material signals were significantly diminished, indicating high conversion; however, the formation of the expected product was low. The crude ^{1}H NMR data agrees with the reported in the literature (S17) for 2-hexene and 3-hexene.

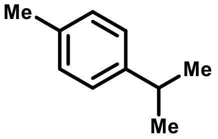

***p*-cymene** CAS No. 99-87-6 (**2u**).
Prepared from 1-methyl-4-(prop-1-en-2-yl)cyclohex-1-ene (54 mg, 0.4 mmol) according to procedure **B** at 110 °C with 1 equivalents of KO^{t}Bu and stirred for 60 min. Colorless liquid (5.4 mg, 10%).
IR (neat, cm^{-1}): 2962, 1518, 1448, 1107, 938.
^{1}H NMR (400 MHz, CDCl3) δ 7.17 – 7.09 (m, 4H), 2.88 (hept, J = 6.9 Hz, 1H), 2.33 (s, 3H), 1.24 (d, J = 6.9 Hz, 6H).
^{13}C NMR (101 MHz, CDCl3) δ 145.84, 135.13, 128.94, 126.29, 33.66, 24.11, 20.93.
LRMS (EI) Calcd for $C_{10}H_{14}$ [M$^+$] *m/z*: 134.11 Found: 134(M).
The spectroscopic data agrees with those reported in the literature (S18).

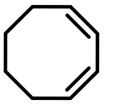

(1*Z*,3*Z*)-cycloocta-1,3-diene CAS No. 1700-10-3 (**2v**).
Prepared from 1.5-cyclooctadiene (43.27 mg, 0.4 mmol) according to procedure **B** at 80 °C with 2 equivalents of KO^{t}Bu and stirred for 2 h. Colorless liquid (43.2 mg, 99%).
IR (neat, cm^{-1}): 3005, 2923, 1446, 668.
^{1}H NMR (400 MHz, CDCl3) δ 5.80 (dd, J = 2.8, 10.6 Hz, 2H), 5.69 – 5.52 (m, 2H), 2.18 (d, J = 3.8 Hz, 4H), 1.52 (d, J = 2.8 Hz, 4H). (m, 4H).
^{13}C NMR (101 MHz, CDCl3) δ 131.13, 125.89, 28.02, 23.09.
LRMS (EI) Calcd for C_8H_{12} [M$^+$] *m/z*: 108.09 Found: 108(M).
The spectroscopic data agrees with those reported in the literature (S8).

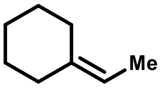

Ethylidenecyclohexane CAS No. 1003-64-1 (**2w**).
Aimed from vinylcyclohexane (33 mg, 0.4 mmol) according to procedure **B** for 2 h. Trials: Try 1: rt & 2 equiv. of base = no reaction. Try 2: 50 °C & 2 equiv. of base = no reaction. Try 3: 80 °C & 2 equiv. of base = no reaction. Try 4: 80 °C & 3 equiv. of base = no reaction.

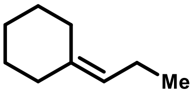

Propylidenecyclohexane CAS No. 5364-83-0 (**2x**).
Aimed from allylcyclohexane (47 mg, 0.4 mmol) according to procedure **B** for 2 h. Try 1: rt & 2 equiv. of base = no reaction. Try 2: 50 °C & 2 equiv. of base = no reaction. Try 3: 80 °C & 2 equiv. of base = no reaction. Try 4: 80 °C & 3 equiv. of base = no reaction. Try 5: At 110 °C with 2 equivalents of base, the reaction produced a mixture of products, as indicated by new ^{1}H NMR signals at 5.36, 5.20, and 4.90 ppm. Although the starting material signals were significantly diminished—suggesting high conversion—the formation of the expected product was low.

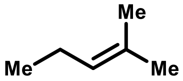

2-methylpent-2-ene CAS No. 625-27-4 (**2y**).
Aimed from 4-methylpent-1-ene (47 mg, 0.4 mmol) according to procedure **B** for 2 h.
Try 1: rt & 2 equiv. of base = no reaction. Try 2: 50 °C & 2 equiv. of base = no reaction. Try 3: 50 °C & 3 equiv. of base = no reaction. Try 4: 80 °C & 2 equiv. of base = mixture of products. The

crude ^{1}H NMR signals for the one and two alkene isomerization were observed. The starting material signals were significantly diminished—suggesting high conversion—but formation of the expected product was low. The crude ^{1}H NMR data agrees with the reported in the literature (S9) for 2-methylpent-2-ene and 3-methylpent-2-ene.

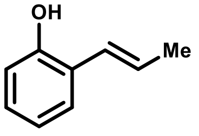

(*E*)-2-(prop-1-en-1-yl)phenol CAS No. 23619-59-2 (**2z**).
Prepared from 2-allylphenol (53.6 mg, 0.4 mmol) according to procedure **B** at 50 °C with 2 equivalents of KO^{t}Bu and stirred for 10 minutes. Pale yellow liquid (46 mg, 85%) as inseparable mixture of isomers with 9:1 (*E/Z*) ratio.
Prepared from 2-allylphenylacetate (70.4 mg, 0.4 mmol) according to procedure **B** at 50 °C with 2 equivalents of base and stirred for 30 minutes. Pale yellow liquid (45 mg, 84%), inseparable mixture of isomers with 9:1 (*E/Z*) ratio.
^{1}H NMR (400 MHz, $CDCl_3$) *Major* (*E*): δ 7.30 (dd, J = 7.7, 1.7 Hz, 1H), 7.09 (td, J = 7.7, 1.7 Hz, 1H), 6.88 (td, J = 7.5, 1.2 Hz, 1H), 6.78 (dd, J = 8.1, 1.2 Hz, 1H), 6.58 (dd, J = 15.9, 1.8 Hz, 1H), 6.20 (dq, J = 15.8, 6.6 Hz, 1H). 1.91 (dd, J = 6.6, 1.8 Hz, 3H). *Minor* (*Z*): δ 7.20 – 7.14 (m, 1H), 6.94 – 6.90 (m, 1H), 6.40 (d, J = 12.9 Hz, 1H), 6.06 – 5.96 (m, 1H), 1.71 (d, J = 1.8 Hz, 3H).
^{13}C NMR (101 MHz, $CDCl_3$) δ 151.98, 127.60, 127.02, 124.91, 124.69, 120.51, 115.27, 18.61.
LRMS (EI) Calcd for $C_9H_{10}O$ [M$^+$] *m/z*: Found: 134(M).
The spectroscopic data agrees with those reported in the literature (S12).

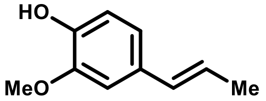

(*E*)-2-methoxy-4-(prop-1-en-1-yl)phenol CAS No. 5932-68-3 (**2ab**).
Prepared from 4-allyl-2-methoxyphenol (65 mg, 0.4 mmol) according to procedure **B** at 50 °C with 2 equivalents of KO^{t}Bu and stirred for 10 minutes. Colorless liquid (54 mg, 83%) as inseparable mixture of isomers with 2.5:1 (*E/Z*) ratio.
^{1}H NMR (400 MHz, $CDCl_3$) δ 6.87 – 6.78 (m, 3H), 6.31 (dd, J = 15.7, 1.7 Hz, 1H), 6.07 (dq, J = 15.7, 6.6 Hz, 1H), 3.88 (s, J = 1.9 Hz, 3H), 1.85 (dd, J = 6.6, 1.7 Hz, 3H).
^{13}C NMR (101 MHz, $CDCl_3$) δ 146.62, 144.80, 130.79, 130.72, 130.15, 129.70, 125.28, 123.55, 122.16, 119.38, 114.42, 114.16, 111.46, 107.89, 77.46, 77.14, 76.82, 55.95, 55.92, 18.48, 14.77.
LRMS (EI) Calcd for $C_{10}H_{12}O_2$ [M$^+$] *m/z*: 164.20 Found: 164(M).
The spectroscopic data agrees with those reported in the literature (S12).

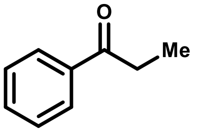

Propiophenone CAS No. 93-55-0 (**2ac**).
Prepared from 1-phenylprop-2-en-1-ol (53.67 mg, 0.4 mmol) according to procedure **B** at 80 °C with 2 equivalents of KO^{t}Bu and stirred for 2 h. Colorless liquid (53.1 mg, 98%).
IR (neat, cm^{-1}): 3063, 2977, 1682, 1217, 688.
^{1}H NMR (400 MHz, $CDCl_3$) δ 7.98 – 7.92 (m, 2H), 7.56 – 7.51 (m, 1H), 7.47 – 7.40 (m, 2H), 2.99 (q, J = 7.3 Hz, 2H), 1.21 (t, J = 7.2 Hz, 3H).
^{13}C NMR (101 MHz, $CDCl_3$) δ 200.46, 136.46, 132.50, 128.15, 128.12, 31.33, 7.82.
LRMS (EI) Calcd for $C_9H_{10}O$ [M$^+$] *m/z*: 134.07 Found: 134(M).
The spectroscopic data agrees with those reported in the literature (S19).

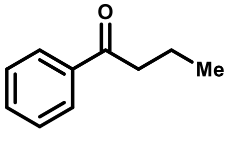

1-phenylbutan-1-one CAS No. 495-40-9 (**2ad**).
Prepared from 1-phenylbut-3-en-1-ol (59.28 mg, 0.4 mmol) according to procedure **B** at 80 °C with 2 equivalents of KO^{t}Bu and stirred for 2 h. Colorless liquid (57.1 mg, 96%).
IR (neat, cm^{-1}): 3066, 2962, 1681, 1212, 688.
^{1}H NMR (400 MHz, $CDCl_3$) δ 7.98 – 7.91 (m, 2H), 7.57 – 7.48 (m, 1H), 7.48 – 7.39 (m, 2H), 2.97 – 2.89 (m, 2H), 1.75 (h, J = 7.4 Hz, 2H), 0.99 (t, J = 7.4 Hz, 3H).
^{13}C NMR (101 MHz, $CDCl_3$) δ 200.07, 136.66, 132.50, 128.16, 127.64, 127.62, 40.11, 17.35, 13.50.
LRMS (EI) Calcd for $C_{10}H_{12}O$ [M$^+$] *m/z*: 148.09 Found: 148(M).
The spectroscopic data agrees with those reported in the literature (S19).

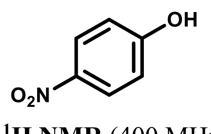

4-nitrophenol CAS No. 100-02-7 (**2ae**).
Prepared from 1-(allyloxy)-4-nitrobenzene (71 mg, 0.4 mmol) according to procedure **B** at 50 °C with 2 equivalents of KO^{t}Bu and stirred for 30 min. White solid (53.3 mg, 88%).
IR (neat, cm^{-1}): 3325, 1608, 1511, 1402, 1258, 522.
^{1}H NMR (400 MHz, DMSO-d_6) δ 10.97 (s, 1H), 8.09 – 8.02 (m, 2H), 6.92 – 6.84 (m, 2H).

^{13}C NMR (101 MHz, DMSO-d_6) δ 163.95, 139.63, 126.24, 115.83.
LRMS (EI) Calcd for $C_{13}H_{16}O_2$ [M^+] *m/z*: 139.03 Found: 139(M).
The spectroscopic data agrees with those reported in the literature (S20).

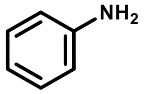

Aniline CAS No. 62-53-3 (**2af**).
Prepared from *N*-allylaniline (53 mg, 0.4 mmol) according to procedure **B** at 50 °C with 1 equivalent of KO^{t}Bu and stirred for 2 h. Pale yellow oil (100% by ^{1}H NMR using mesitylene as internal standard, and 21.2 mg, 57% isolated yield).
IR (neat, cm^{-1}): 3442, 3362, 3050, 1619, 1278, 764.
^{1}H NMR (400 MHz, DMSO-d_6) δ 7.02 – 6.92 (m, 2H), 6.56 – 6.50 (m, 2H), 6.45 (tt, J = 1.1, 7.2 Hz, 1H), 4.97 (s, 2H).
^{13}C NMR (101 MHz, DMSO-d_6) δ 148.64, 129.02, 115.78, 113.98.
LRMS (EI) Calcd for C_6H_7 [M^+] *m/z*: 93.13 Found: 93(M).
The spectroscopic data agrees with those reported in the literature (S11).

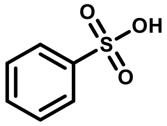

Benzenesulfonic acid CAS No. 98-11-3 (2ag).
Prepared from (allylsulfonyl)benzene (79 mg, 0.4 mmol) according to procedure **A**. White solid (60 mg, 95%).
^{1}H NMR (400 MHz, DMSO-d6) 7.57 – 7.55 (m, 2H), 7.32 – 7.28 (d, 3H).
^{13}C NMR (101 MHz, DMSO-d6) δ 147.48, 129.28, 128.16, 125.76.
LRMS (EI) Calcd for $C_6H_6O_3S$ [M^+] *m/z*: 158 Found: 158(M).
The spectroscopic data agrees with those reported in the literature (S21).

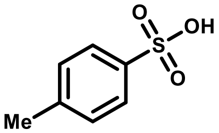

4-methylbenzenesulfonic acid CAS No. 104-15-4 (2ah).
Prepared from 5-(2-(tosylmethyl)allyl)benzo[d][1,3]dioxole (132 mg, 0.4 mmol) according to procedure **A**. White solid (65.8 mg, 96%).
^{1}H NMR (400 MHz, DMSO-d6) δ 7.45 – 7.41 (m, 2H), 7.07 (d, J = 7.9 Hz, 2H), 2.25 (s, 3H).
^{13}C NMR (101 MHz, DMSO-d6) δ 144.98, 138.26, 128.34, 125.61, 20.92.
LRMS (EI) Calcd for $C_7H_8O_3S$ [M^+] *m/z*: 172.20 Found: 172(M).
The spectroscopic data agrees with those reported in the literature (S22).

5. ^{1}H and ^{13}C NMR Spectra of the Observed Adducts

Figure S1. ^{1}H NMR spectrum of (*E*)-prop-1-en-1-ylbenzene **(2a)** in $CDCl_3$

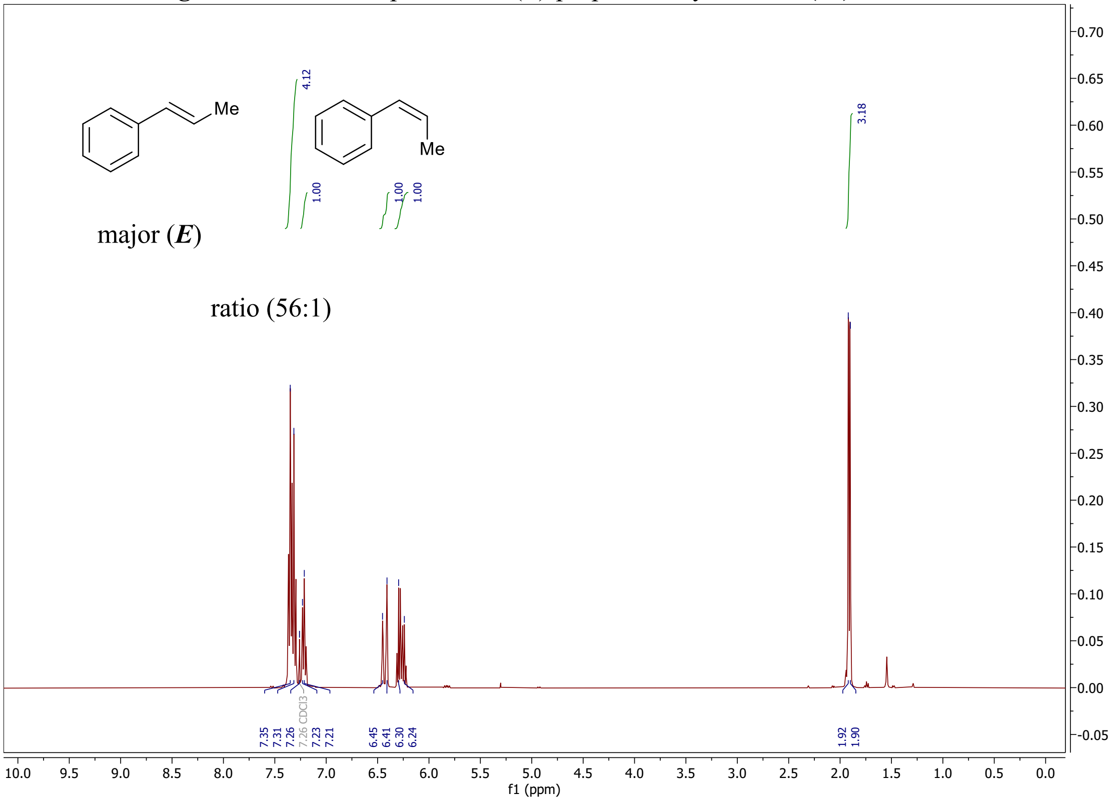

Figure S2. ^{13}C NMR spectrum of (*E*)-prop-1-en-1-ylbenzene **(2a)** in $CDCl_3$

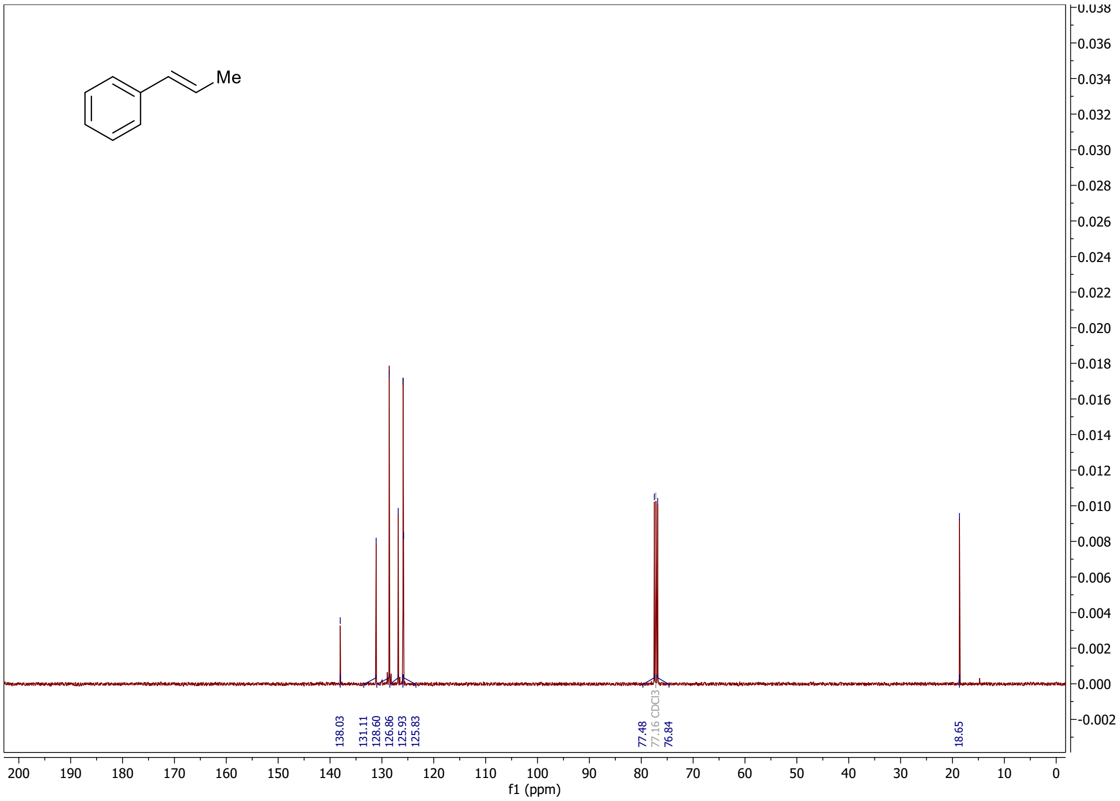

Figure S3. ^{1}H NMR spectrum of (*E*)-1-methyl-4-(prop-1-en-1-yl)benzene **(2b)** in $CDCl_3$

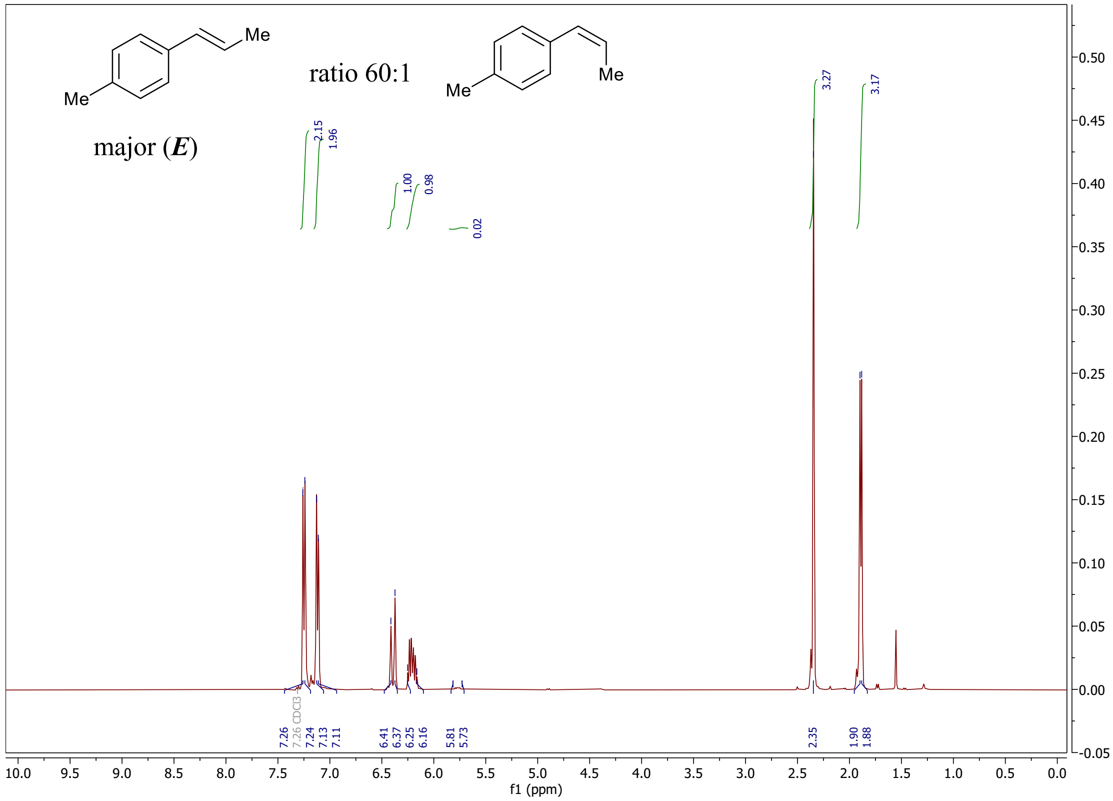

Figure S4. ^{13}C NMR spectrum of (*E*)-1-methyl-4-(prop-1-en-1-yl)benzene **(2b)** in $CDCl_3$

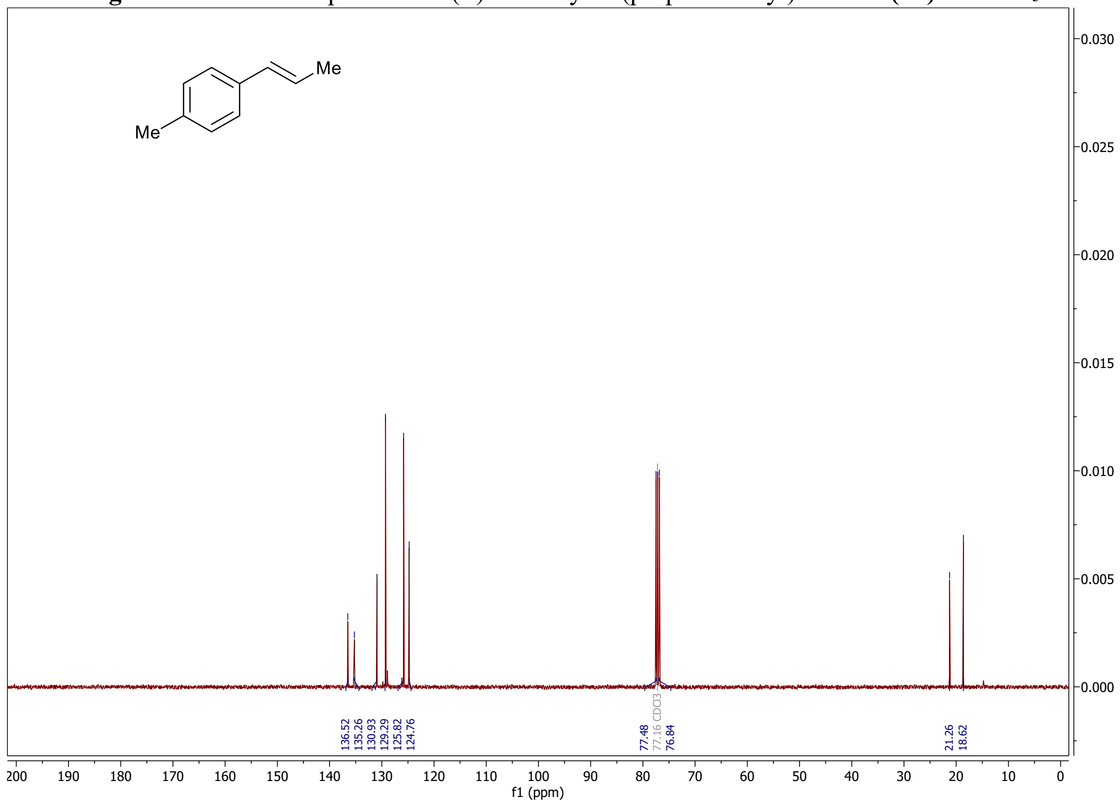

Figure S5. ^{1}H NMR spectrum of (*E*)-1-(prop-1-en-1-yl)-4-(trifluoromethyl)benzene **(2c)** in $CDCl_3$

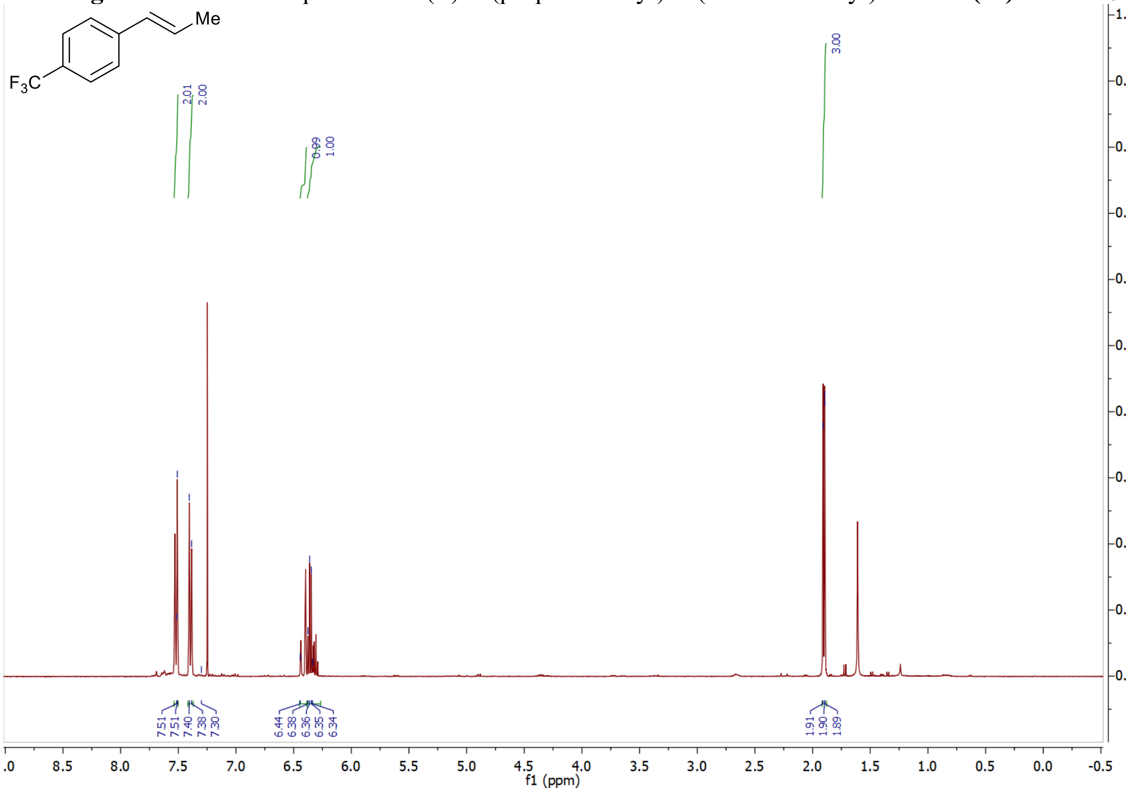

Figure S6. ^{13}C NMR spectrum of (*E*)-1-(prop-1-en-1-yl)-4-(trifluoromethyl)benzene **(2c)** in $CDCl_3$

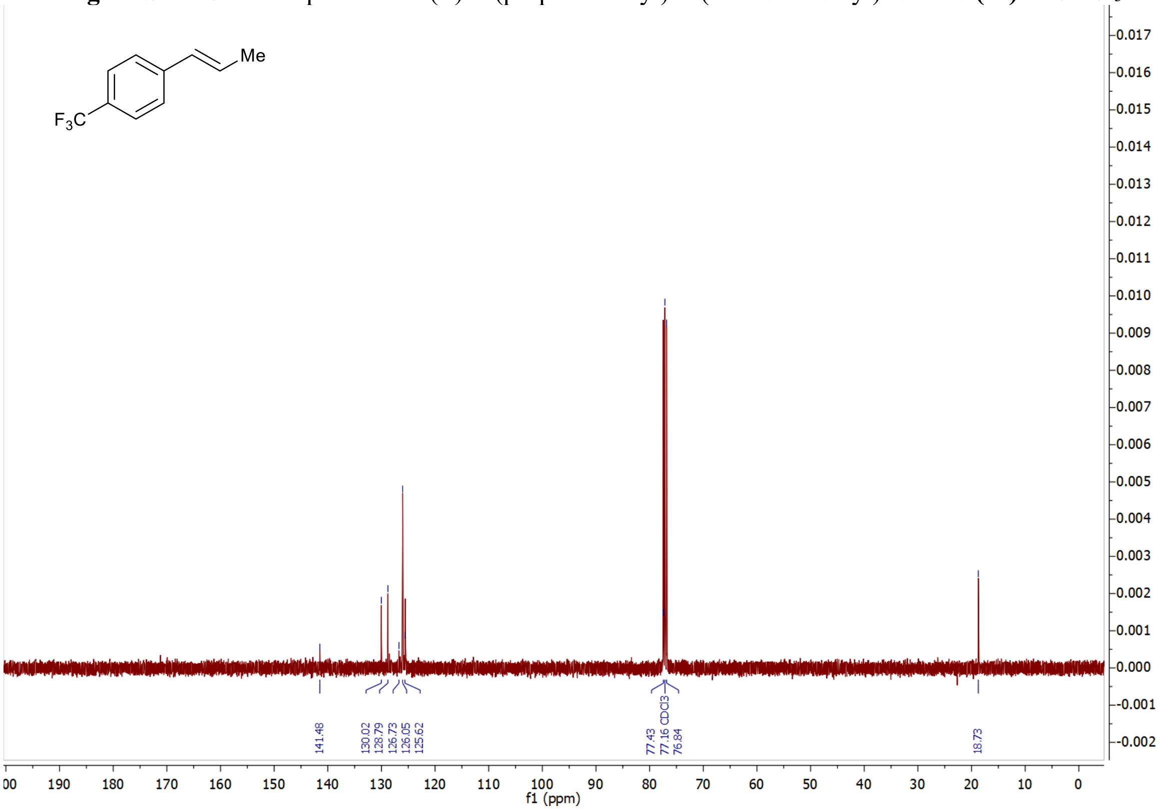

Figure S7. ^{1}H NMR spectrum of (*E*)-1-fluoro-4-(prop-1-en-1-yl)benzene **(2d)** in CDCl3

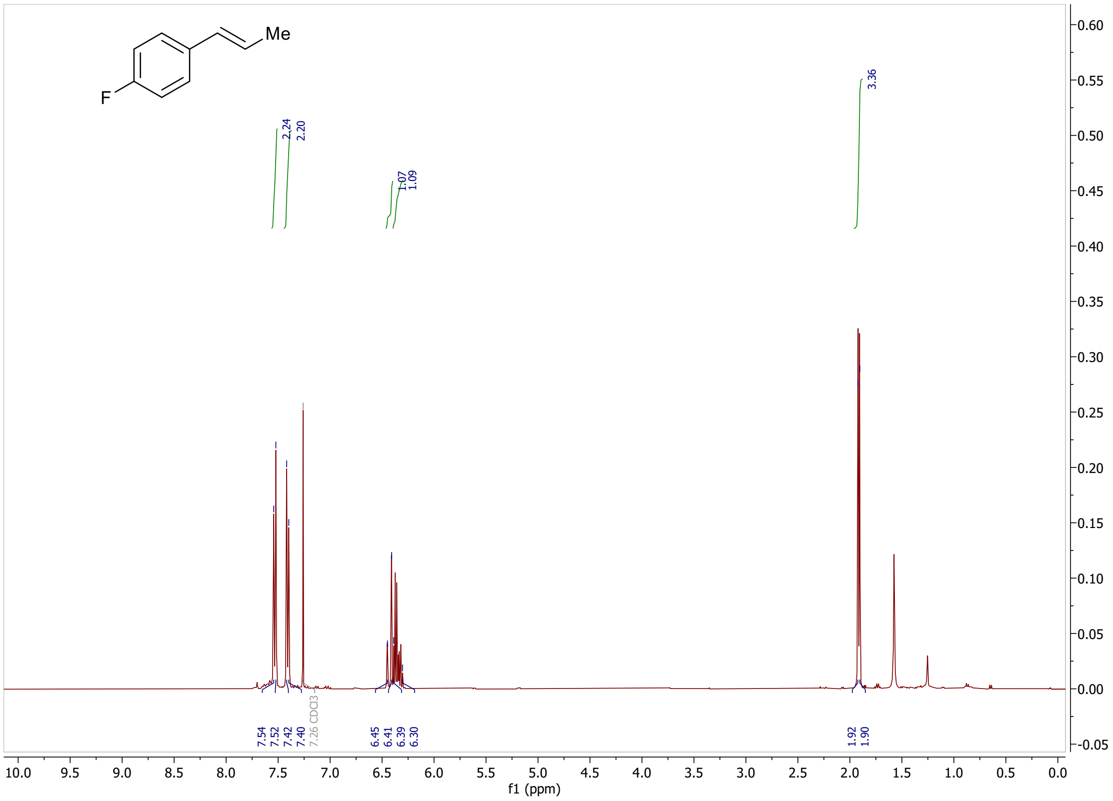

Figure S8. ^{13}C NMR spectrum of (*E*)-1-fluoro-4-(prop-1-en-1-yl)benzene **(2d)** in $CDCl_3$

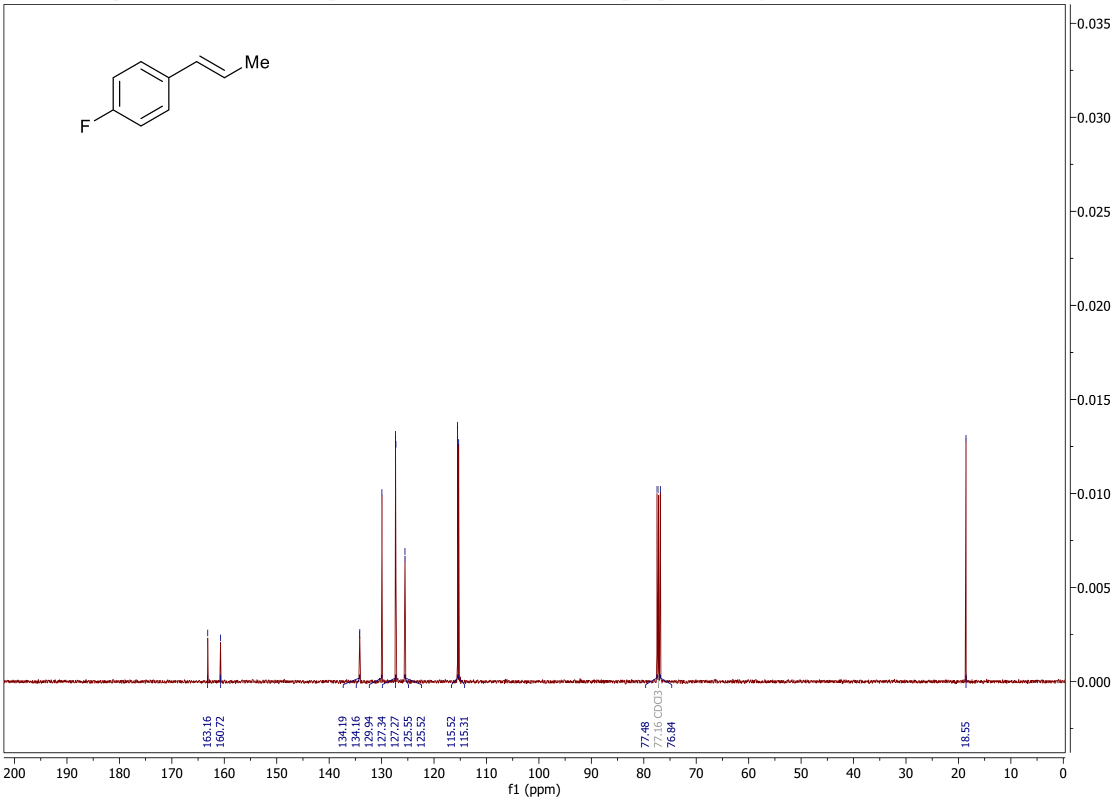

Figure S9. ^{1}H NMR spectrum of (*E*)-1-methoxy-4-(prop-1-en-1-yl)benzene **(2e)** in $CDCl_3$

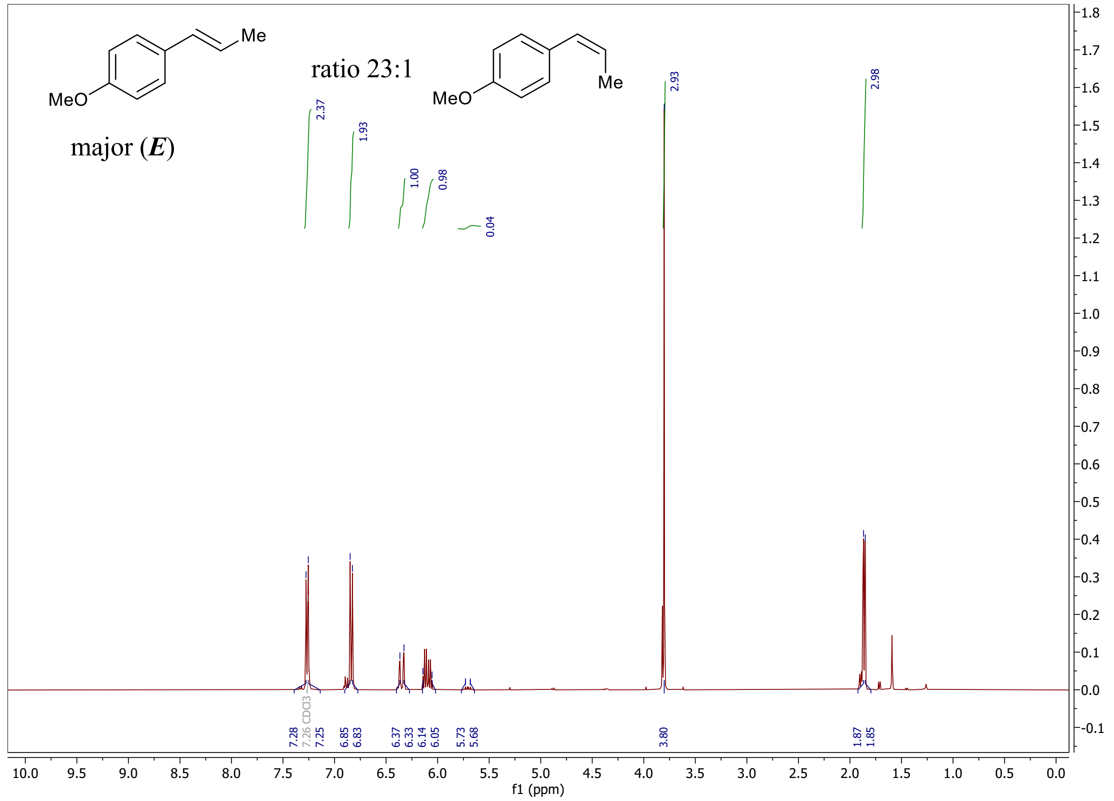

Figure S10. ^{13}C NMR spectrum of (*E*)-1-methoxy-4-(prop-1-en-1-yl)benzene **(2e)** in $CDCl_3$

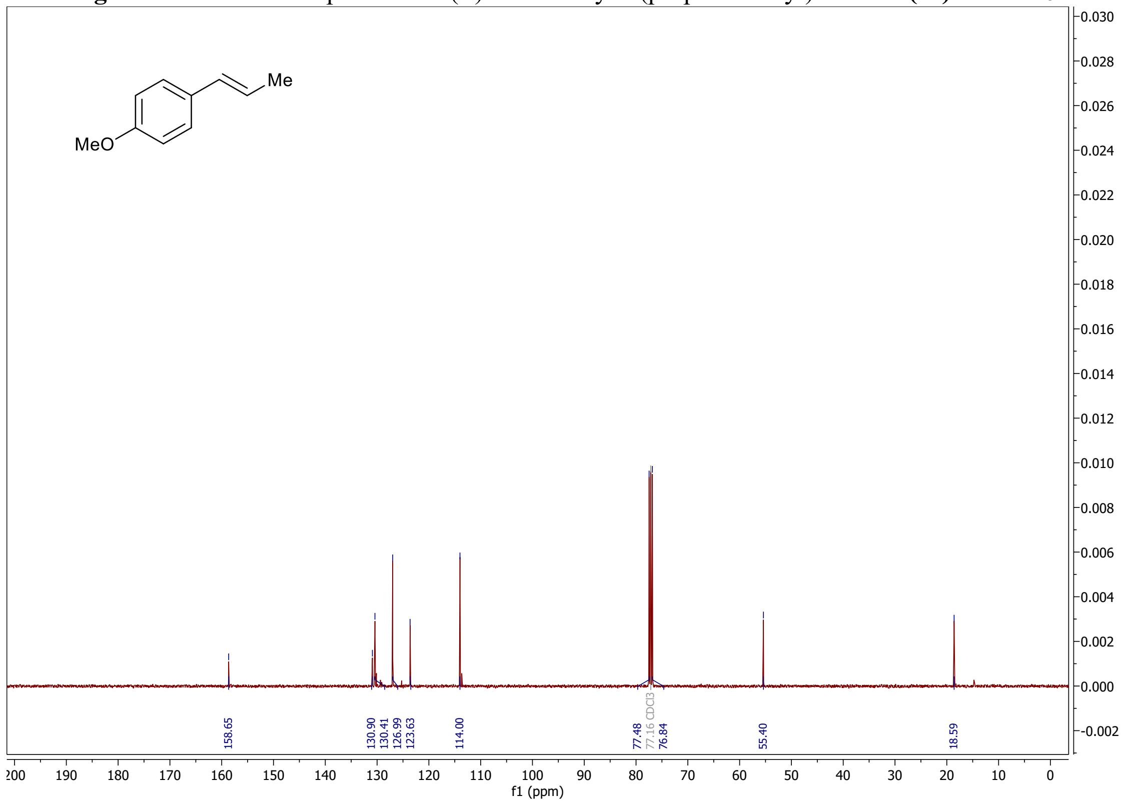

Figure S11. ^{1}H NMR spectrum of (*E*)-1-methoxy-2-(prop-1-en-1-yl)benzene **(2f)** in $CDCl_3$

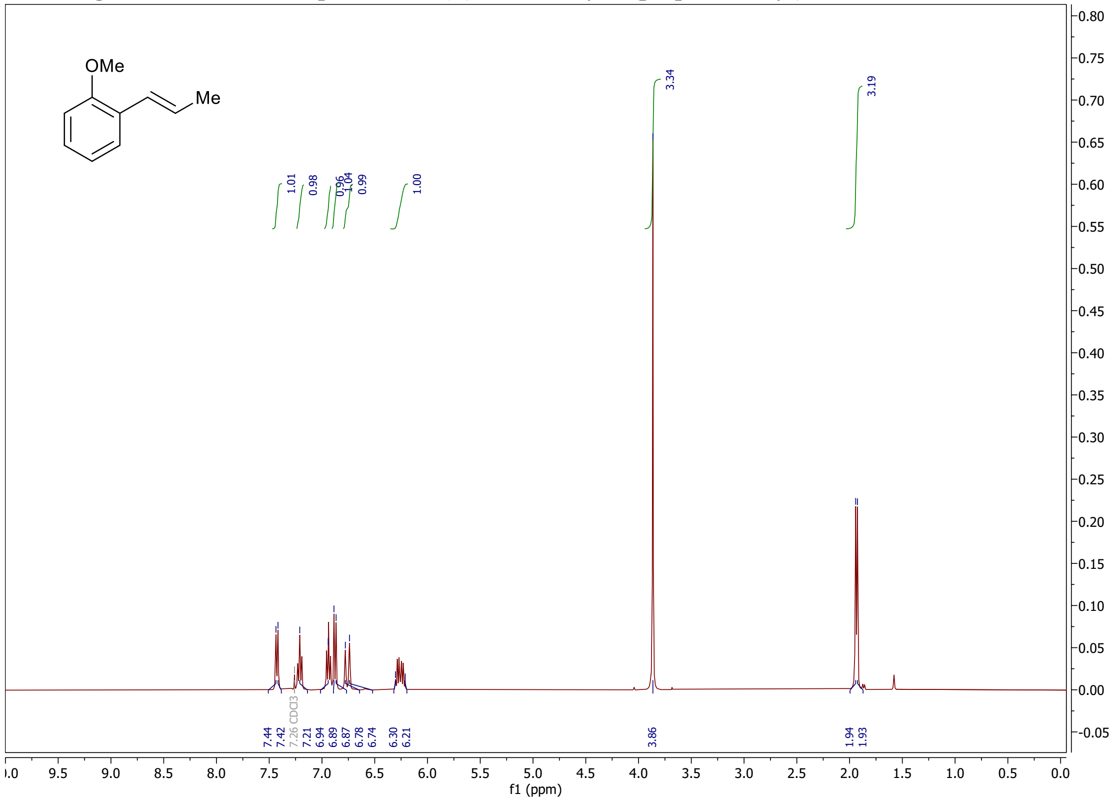

Figure S12. ^{13}C NMR spectrum of (*E*)-1-methoxy-2-(prop-1-en-1-yl)benzene **(2f)** in $CDCl_3$

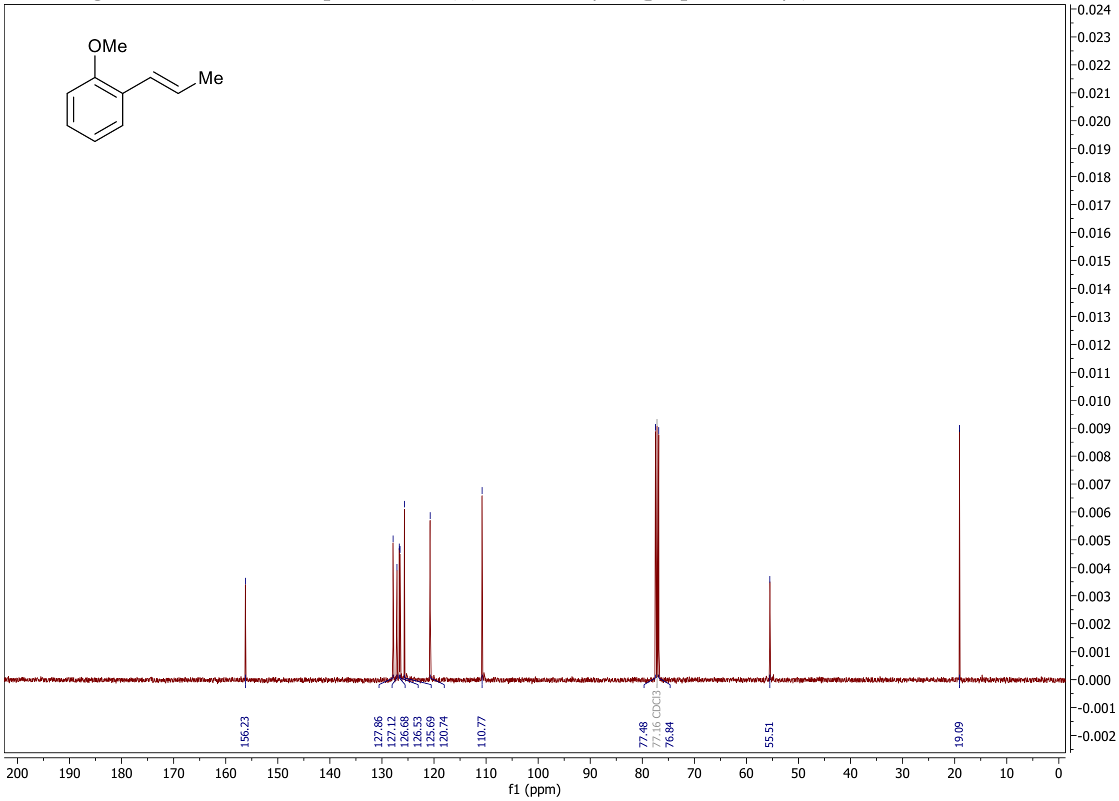

Figure S13. ^{1}H NMR spectrum of (*E*)-1-bromo-2-(prop-1-en-1-yl)benzene **(2g)** in $CDCl_3$

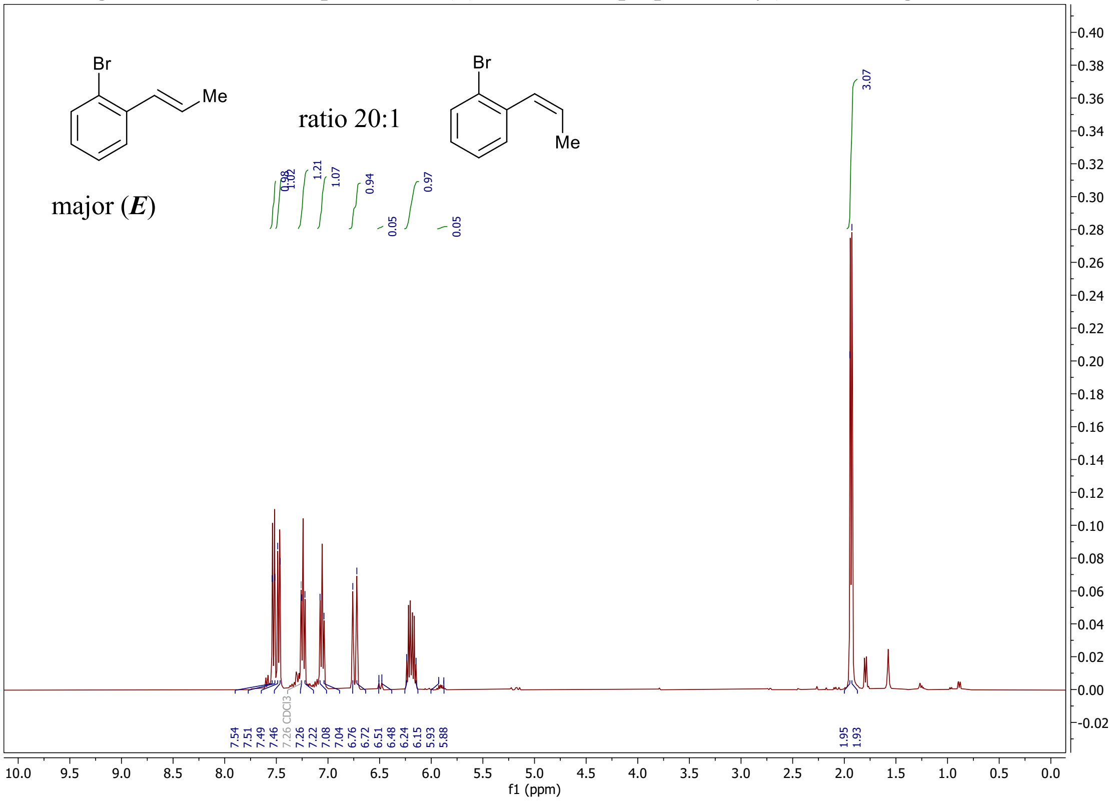

Figure S14. ^{13}C NMR spectrum of (*E*)-1-bromo-2-(prop-1-en-1-yl)benzene **(2g)** in $CDCl_3$

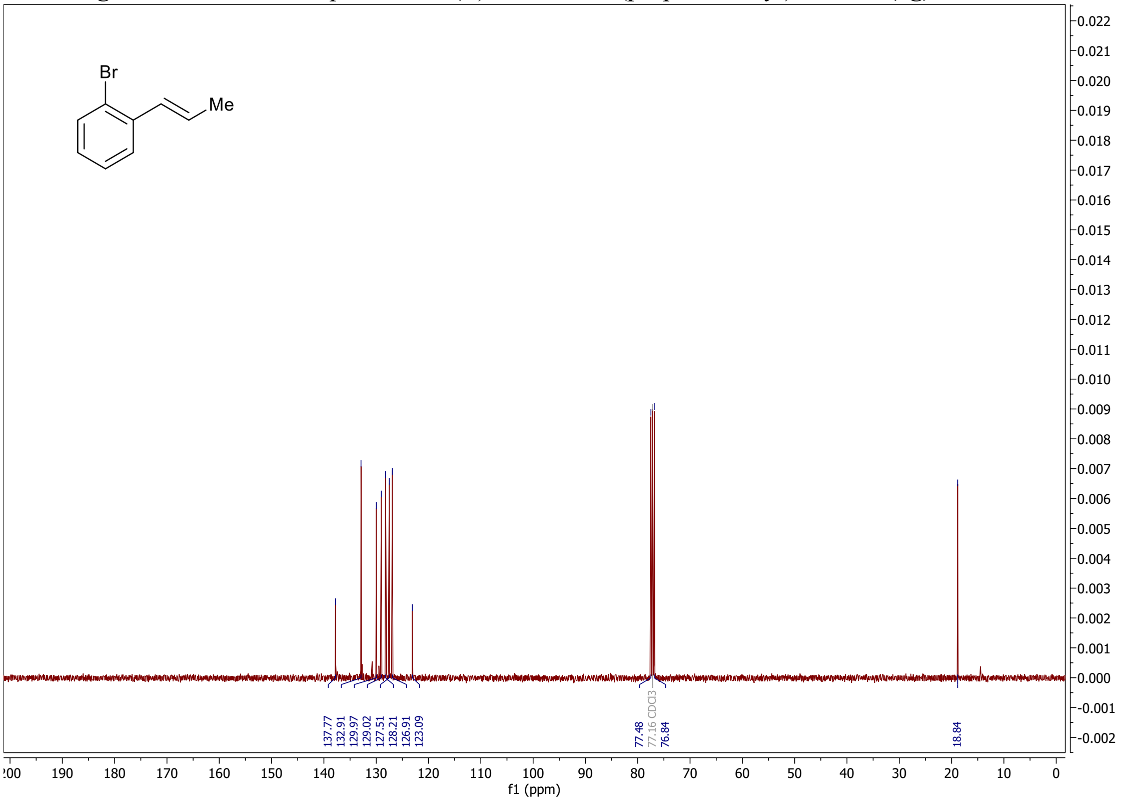

Figure S15. ^{1}H NMR spectrum of (*E*)-1,2-dimethoxy-4-(prop-1-en-1-yl)benzene **(2h)** in $CDCl_3$

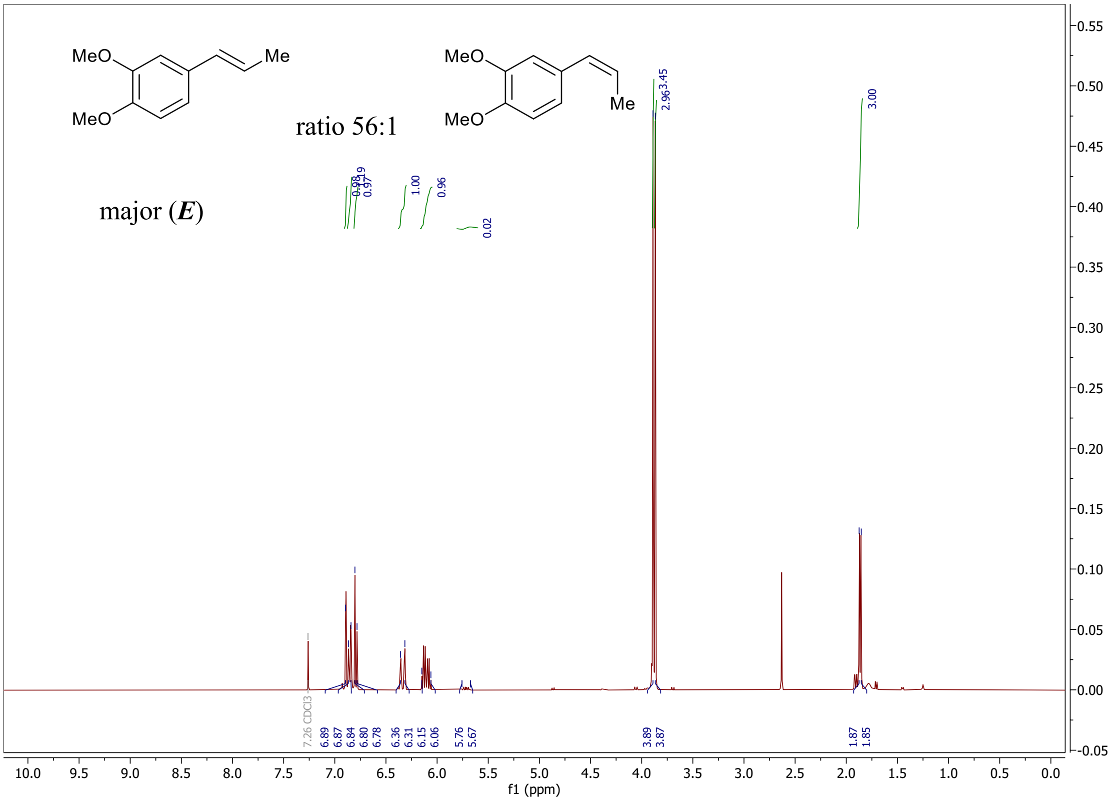

Figure S16. ^{13}C NMR spectrum of (*E*)-1,2-dimethoxy-4-(prop-1-en-1-yl)benzene **(2h)** in $CDCl_3$

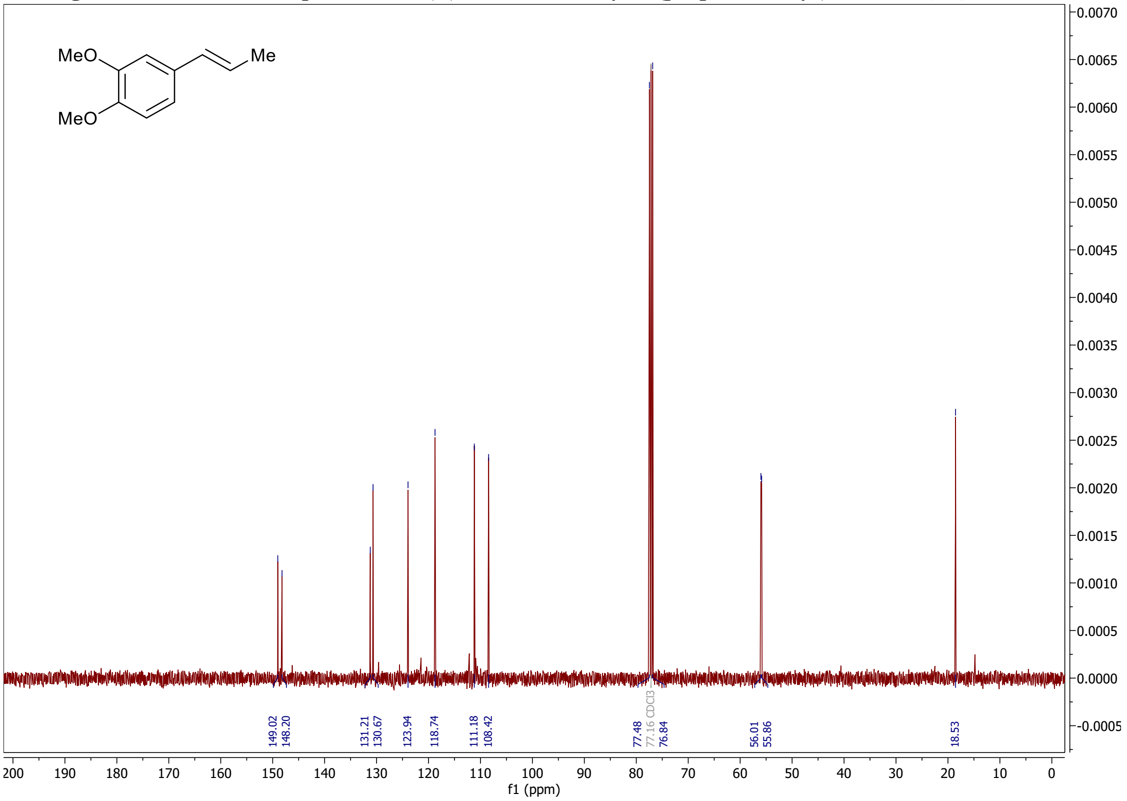

Figure S17. ^{1}H NMR spectrum of (*E*)-5-(prop-1-en-1-yl)benzo[*d*][1,3]dioxole **(2i)** in $CDCl_3$

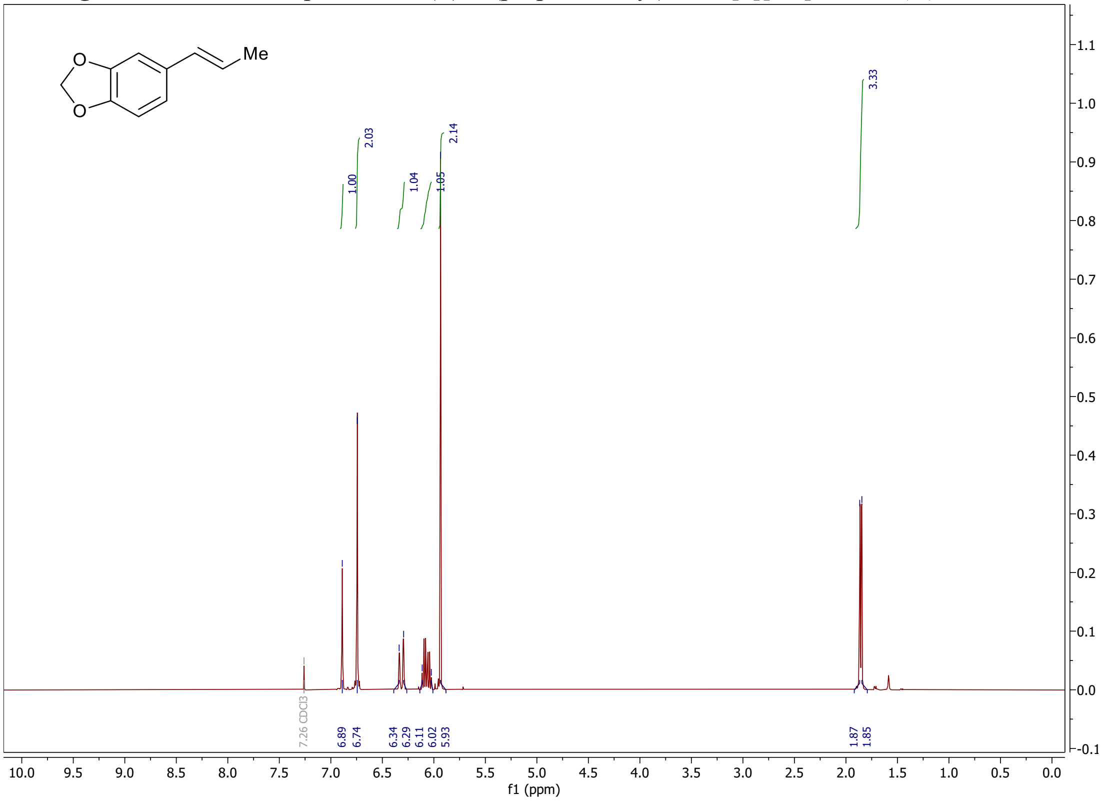

Figure S18. ^{13}C NMR spectrum of (*E*)-5-(prop-1-en-1-yl)benzo[*d*][1,3]dioxole **(2i)** in $CDCl_3$

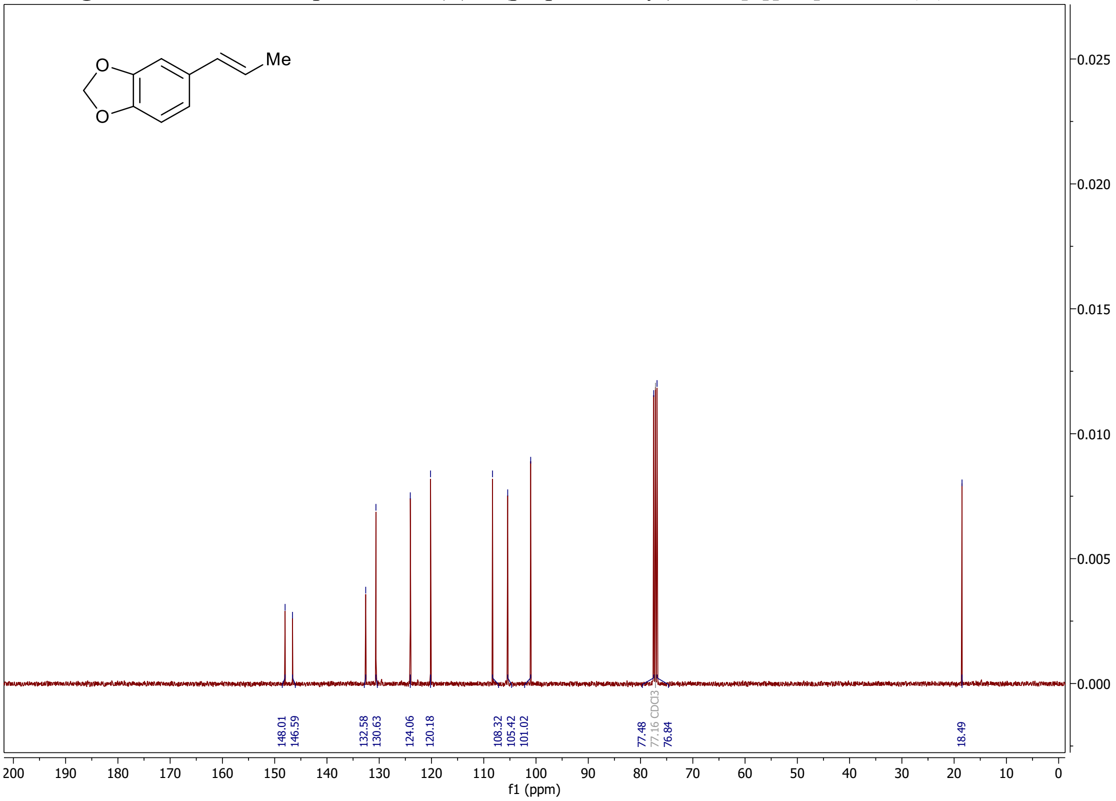

Figure S19. ^{1}H NMR spectrum of (*E*)-1,3,5-trimethyl-2-(prop-1-en-1-yl)benzene **(2j)** in $CDCl_3$

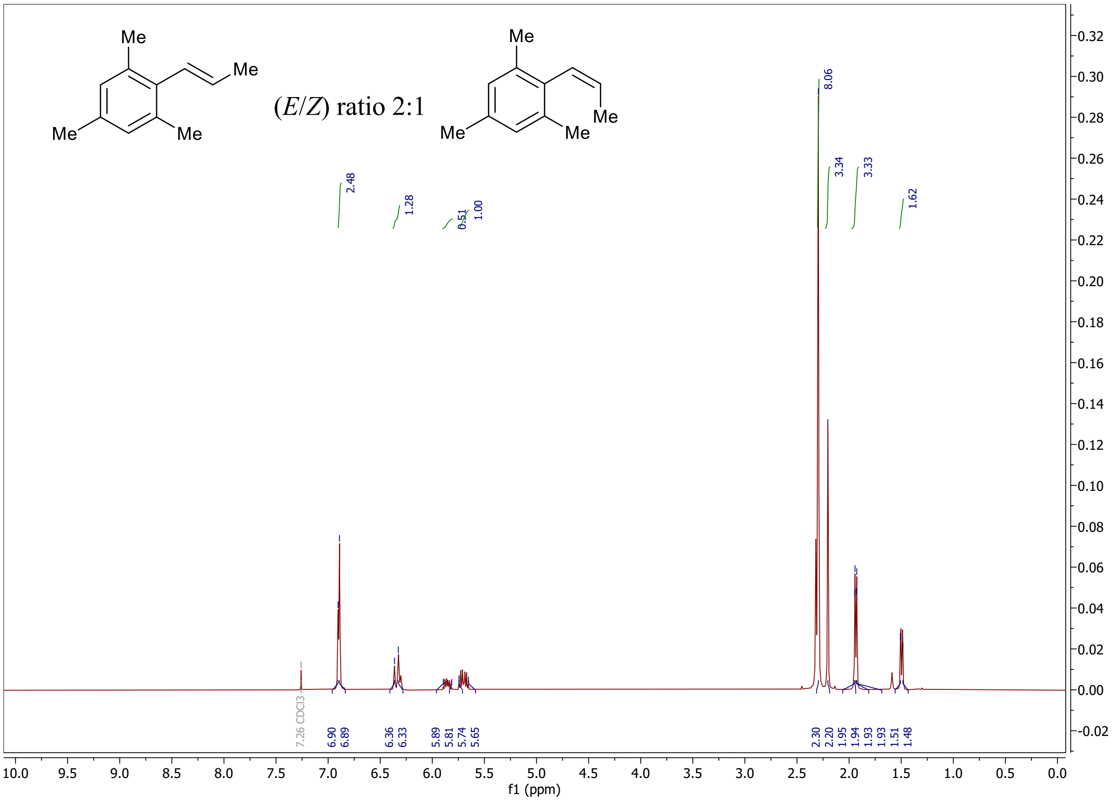

Figure S20. ^{13}C NMR spectrum of (*E*)-1,3,5-trimethyl-2-(prop-1-en-1-yl)benzene **(2j)** in $CDCl_3$

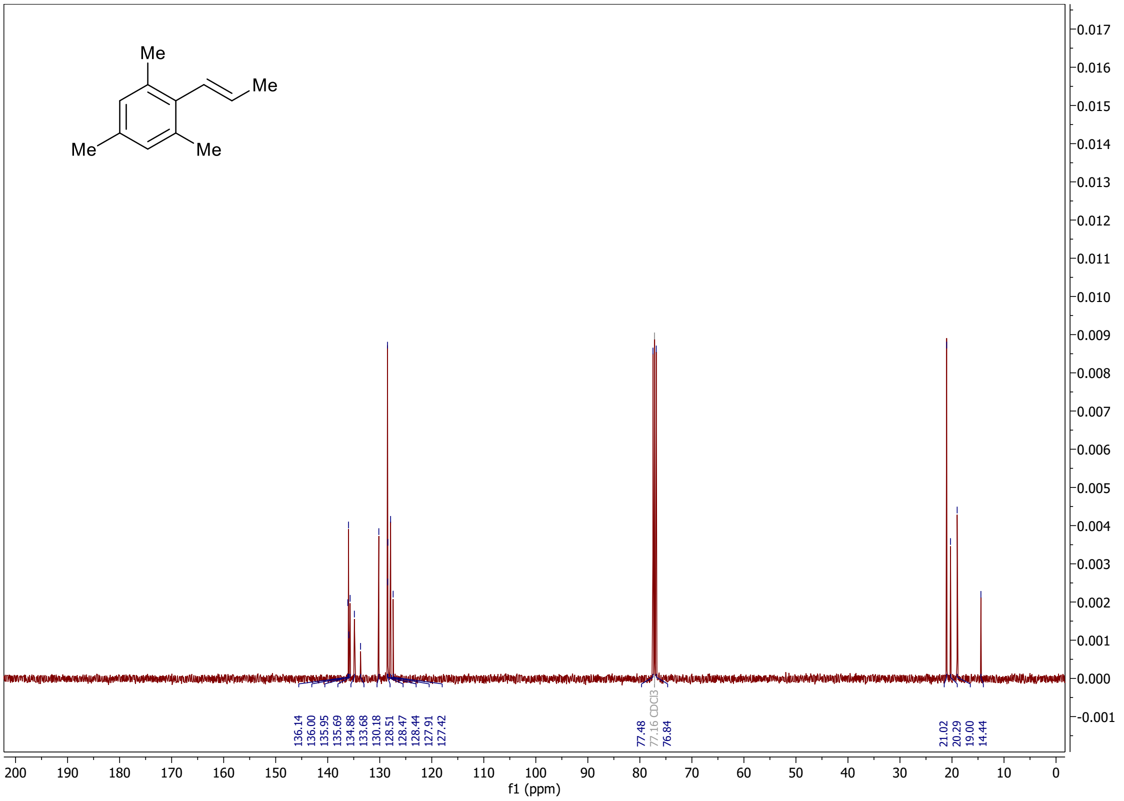

Figure S21. ^{1}H NMR spectrum of (*E*)-1-(prop-1-en-1-yl)naphthalene **(2k)** in $CDCl_3$

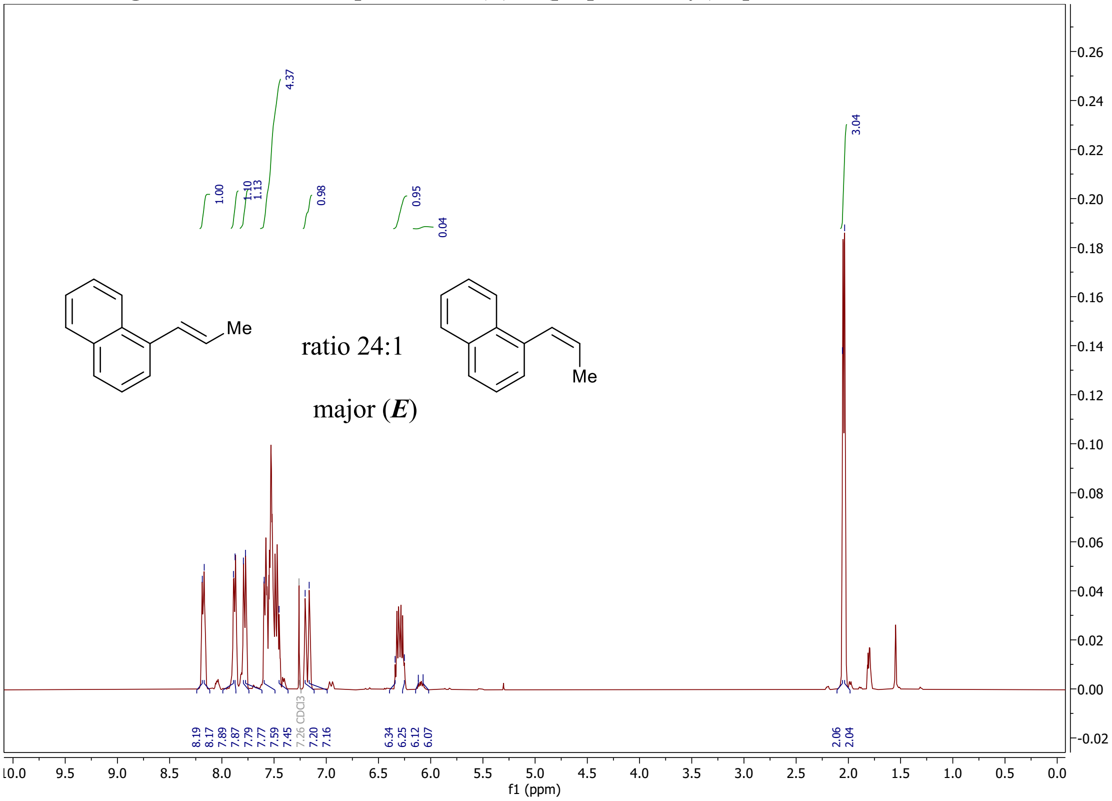

Figure S22. ^{13}C NMR spectrum of (*E*)-1-(prop-1-en-1-yl)naphthalene **(2k)** in $CDCl_3$

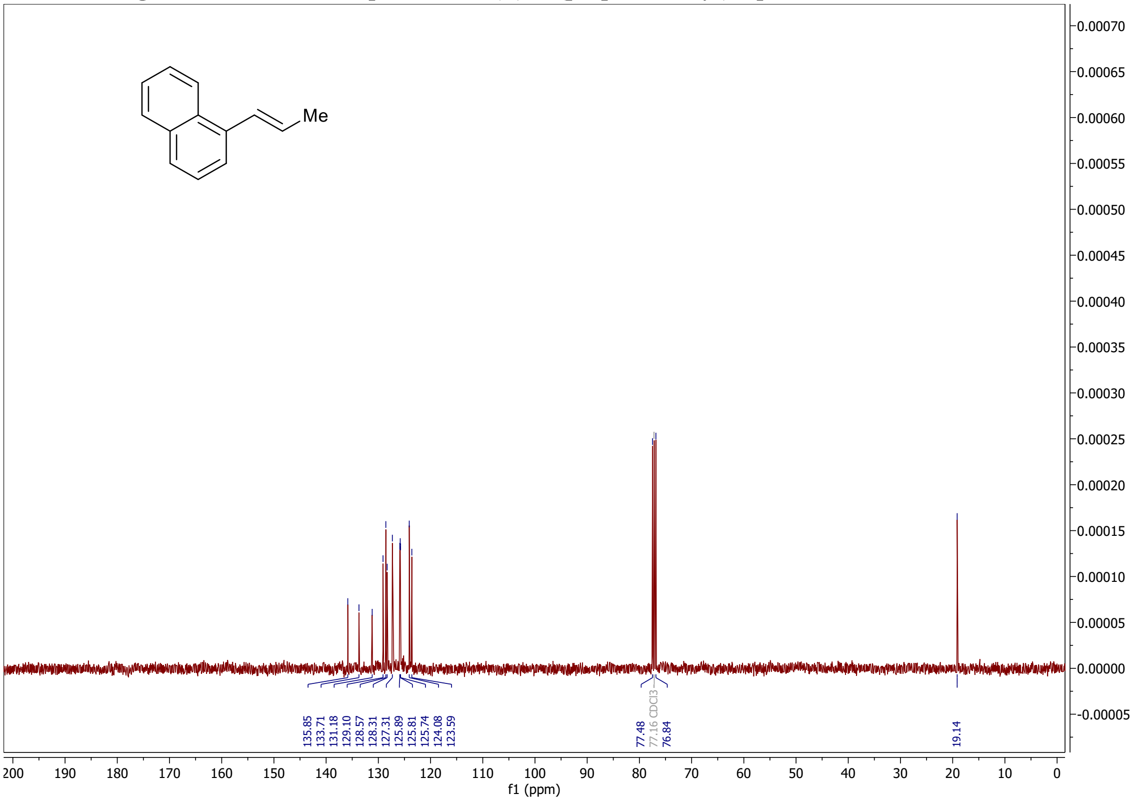

Figure S23. ^{1}H NMR spectrum of (2-methylprop-1-en-1-yl)benzene **(2l)** in $CDCl_3$

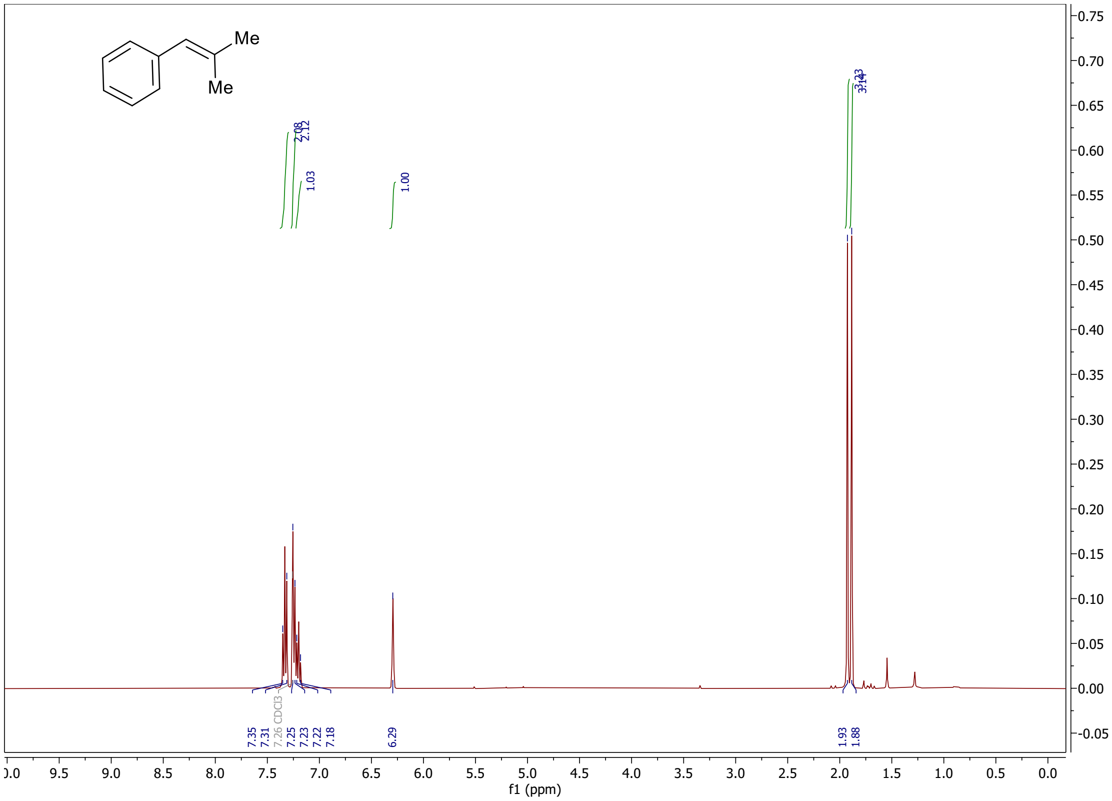

Figure S24. ^{13}C NMR spectrum of (2-methylprop-1-en-1-yl)benzene **(2l)** in $CDCl_3$

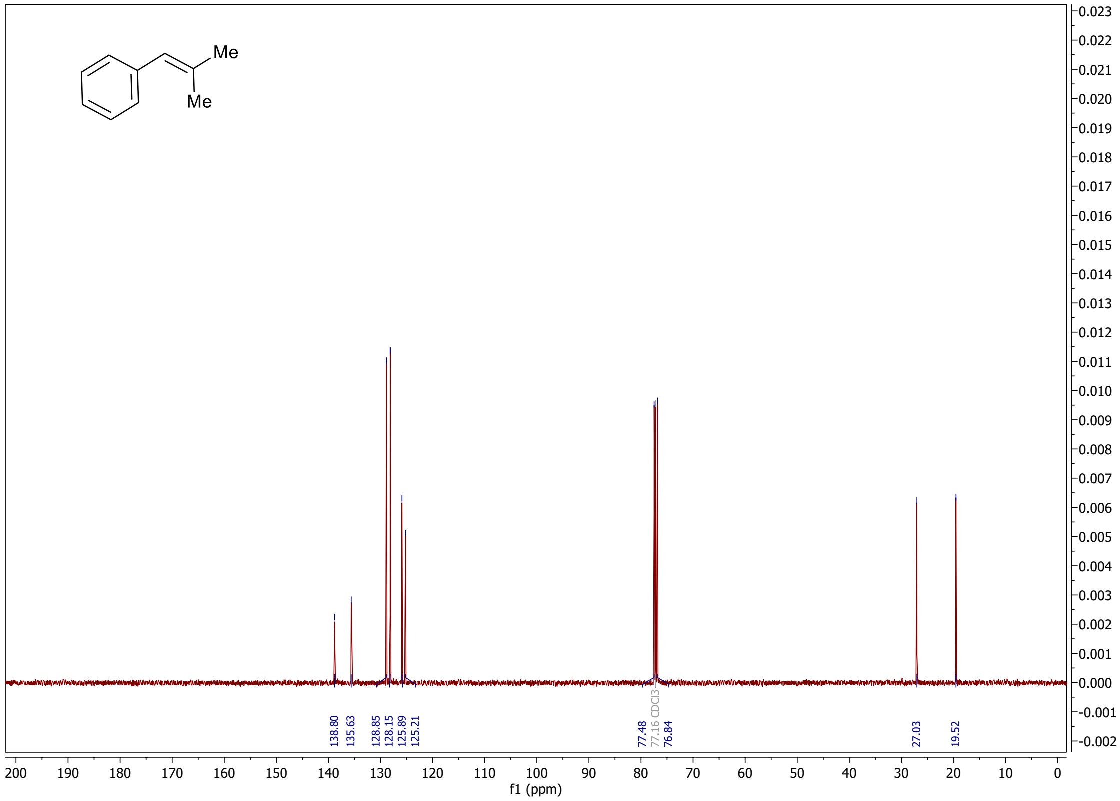

Figure S25. ^{1}H NMR spectrum of *(E)*-3-(prop-1-en-1-yl)pyridine **(2m)** in $CDCl_3$

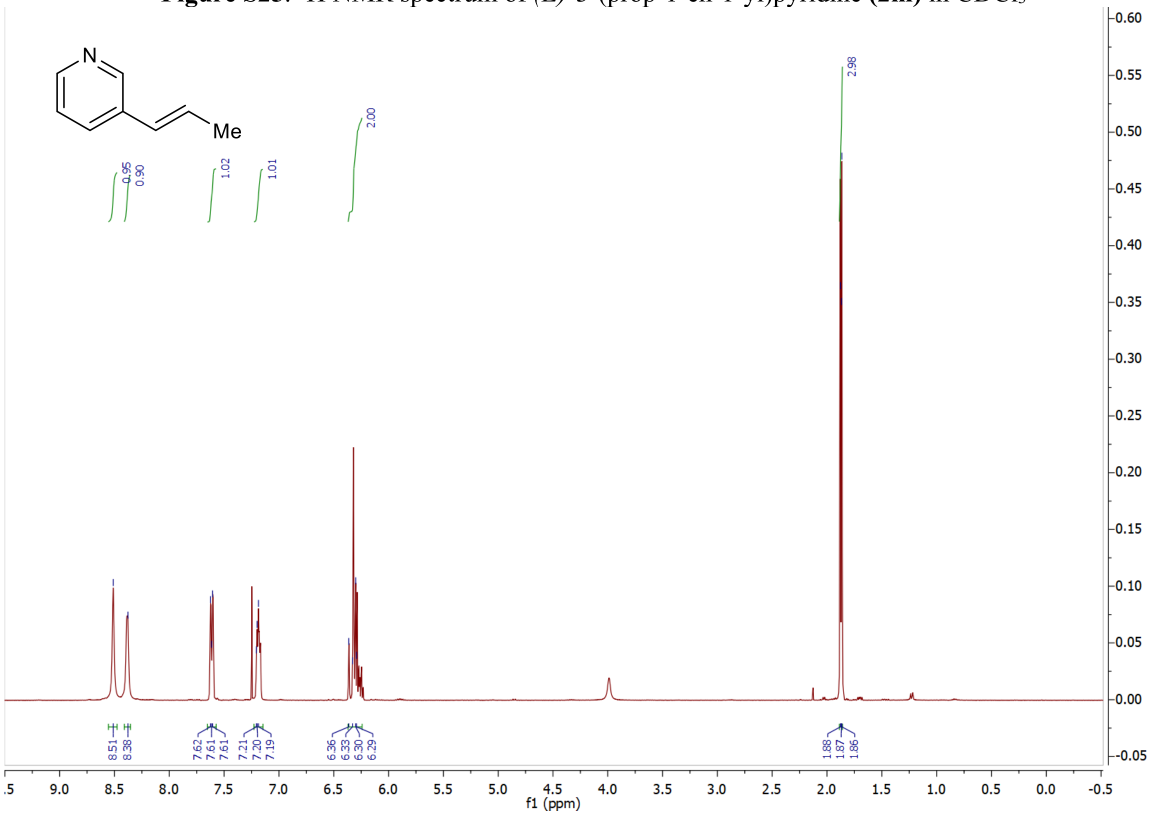

Figure S26. ^{13}C NMR spectrum of *(E)*-3-(prop-1-en-1-yl)pyridine **(2m)** in $CDCl_3$

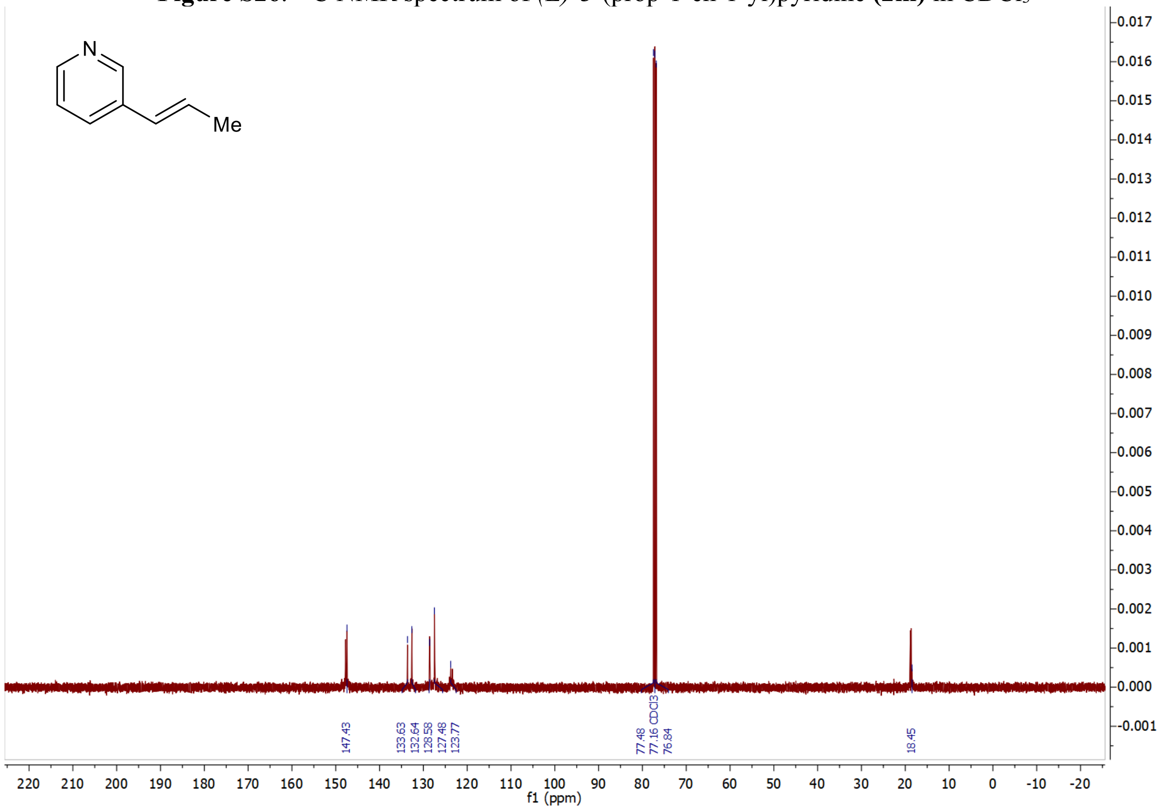

Figure S27. ^{1}H NMR spectrum of (*E*)-1-(prop-1-en-1-yl)-1H-imidazole **(2n)** in $CDCl_3$

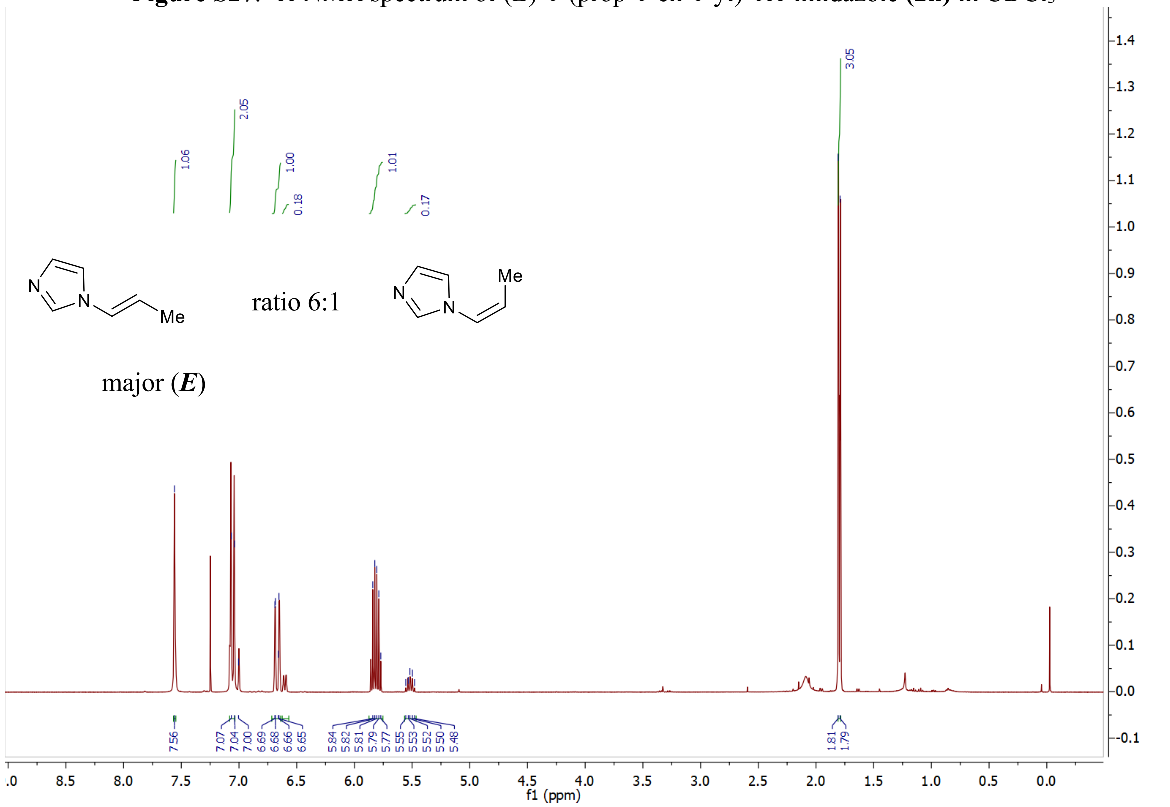

Figure S28. ^{13}C NMR spectrum of (*E*)-1-(prop-1-en-1-yl)-1H-imidazole **(2n)** in $CDCl_3$

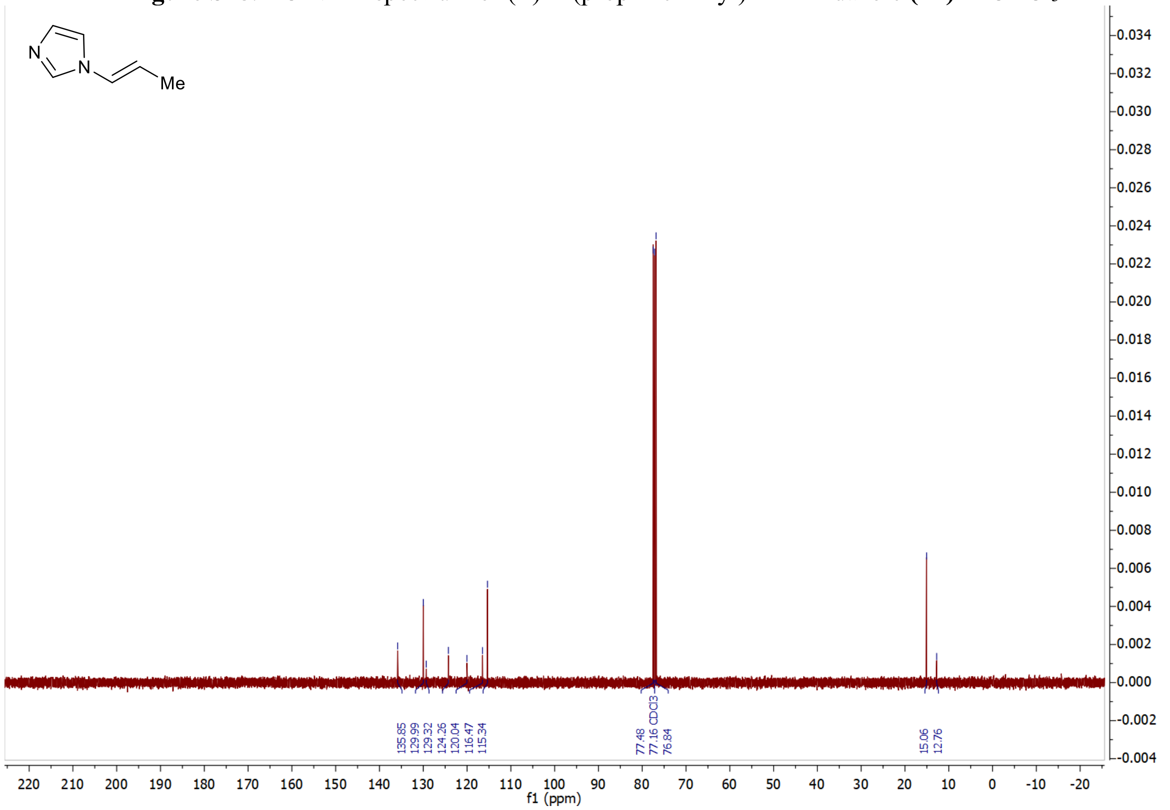

Figure S29. ^{1}H NMR spectrum of (*E*)-phenyl(prop-1-en-1-yl)sulfane **(2o)** in $CDCl_3$

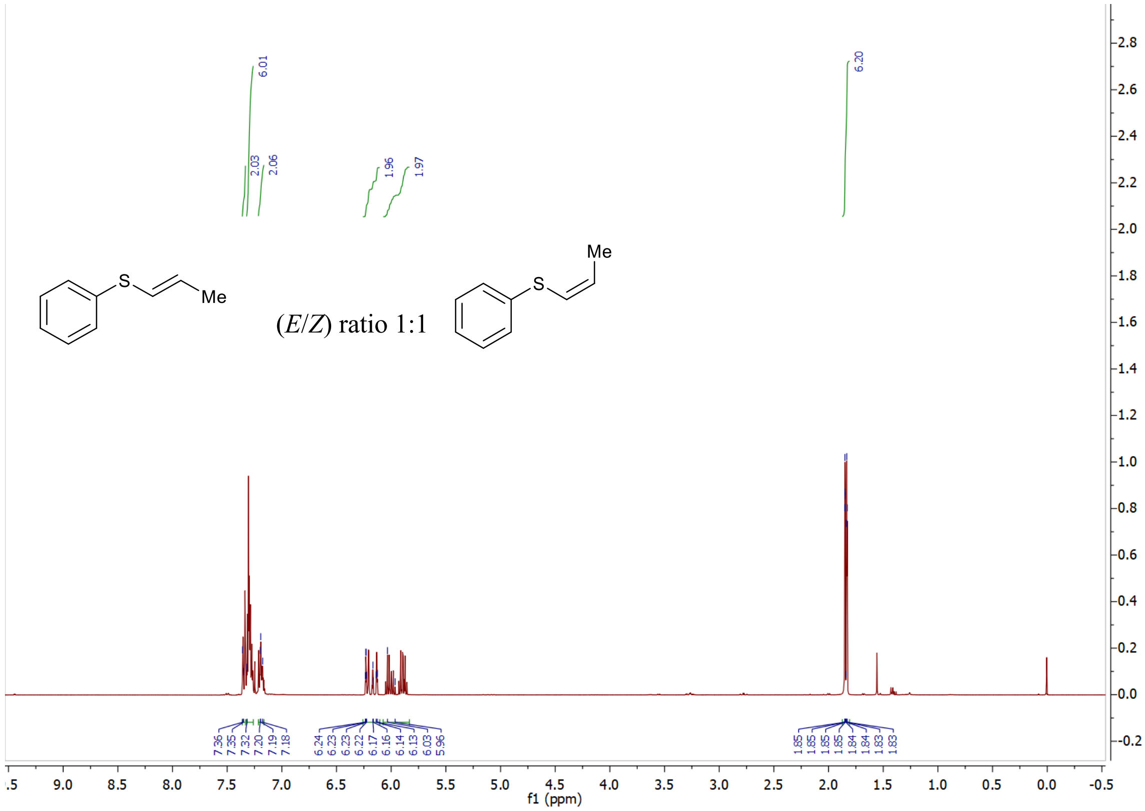

Figure S30. ^{13}C NMR spectrum of (*E*)-phenyl(prop-1-en-1-yl)sulfane **(2o)** in $CDCl_3$

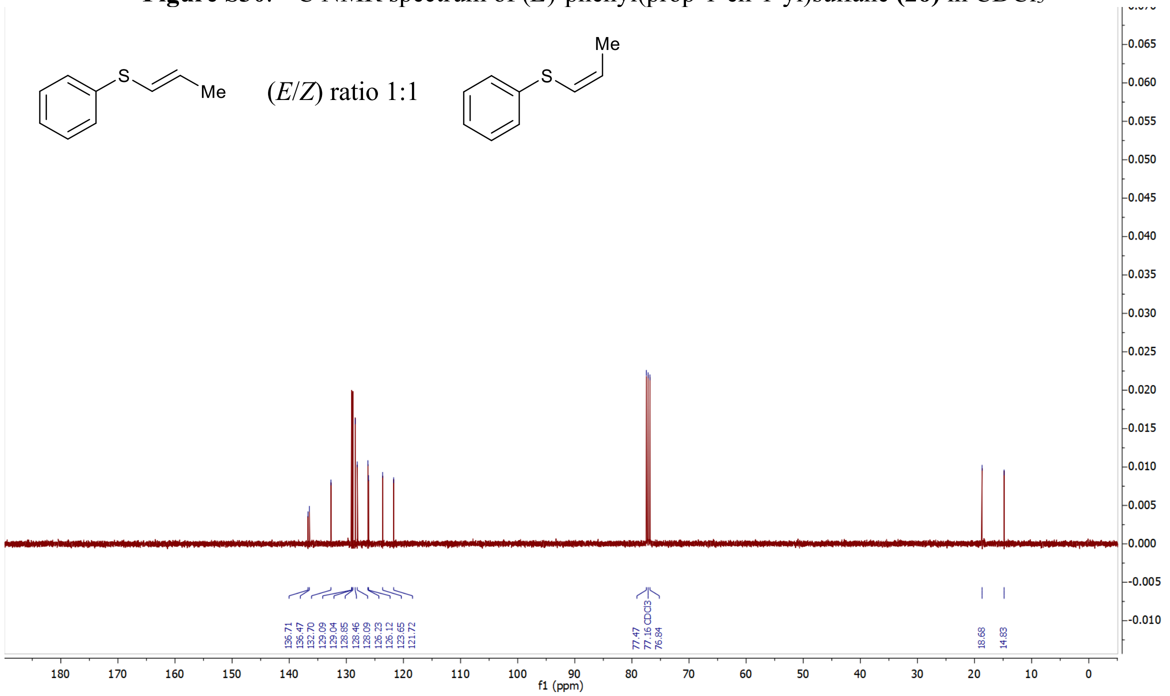

Figure S31. ^{1}H NMR spectrum of (*Z*)-(prop-1-en-1-yloxy)benzene **(2p)** in $CDCl_3$

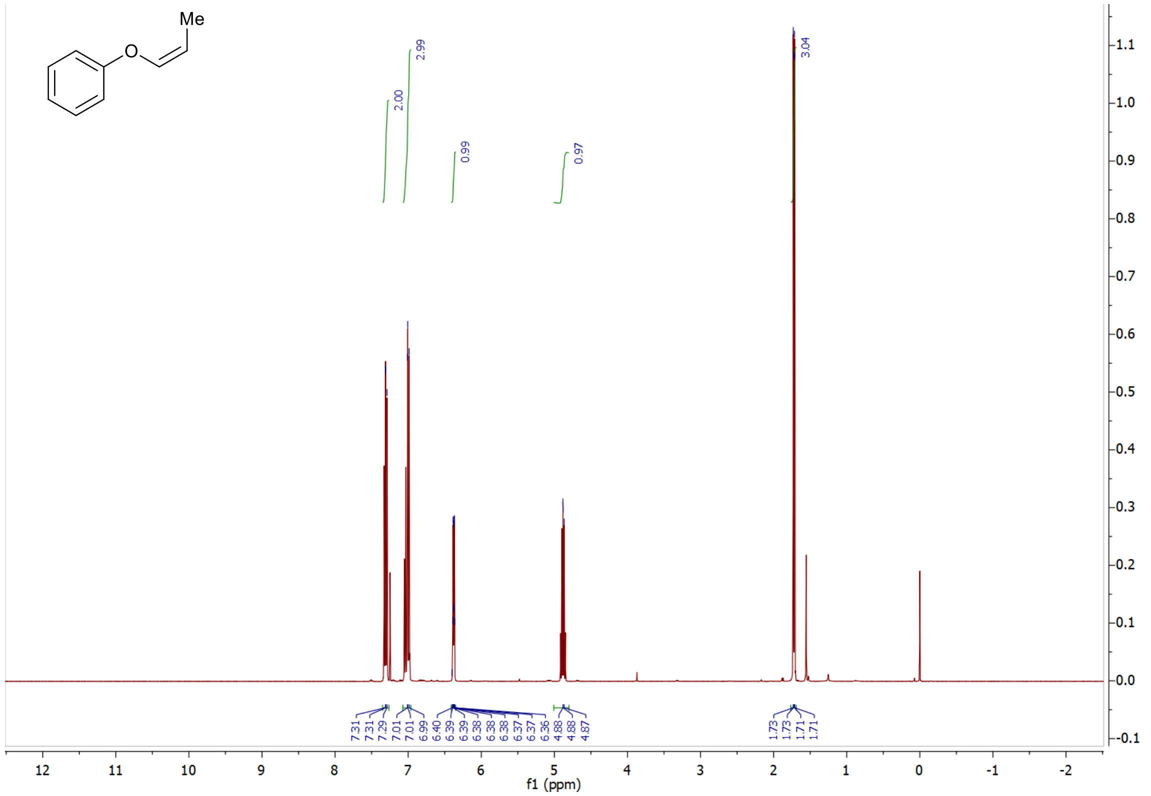

Figure S32. ^{13}C NMR spectrum of (*Z*)-(prop-1-en-1-yloxy)benzene **(2p)** in $CDCl_3$

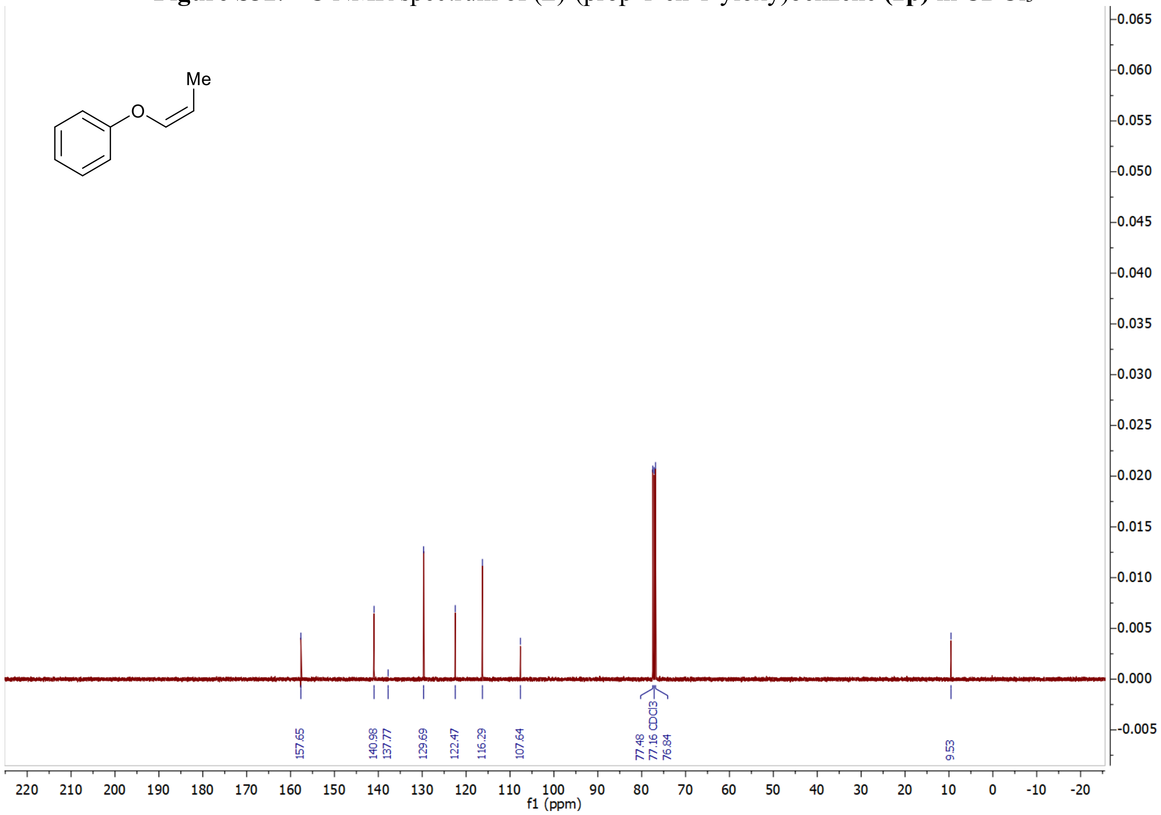

Figure S33. ^{1}H NMR spectrum of 1-((*E*)-prop-1-en-1-yl)-2-(((*Z*)-prop-1-en-1-yl)oxy)benzene **(2q)** in $CDCl_3$

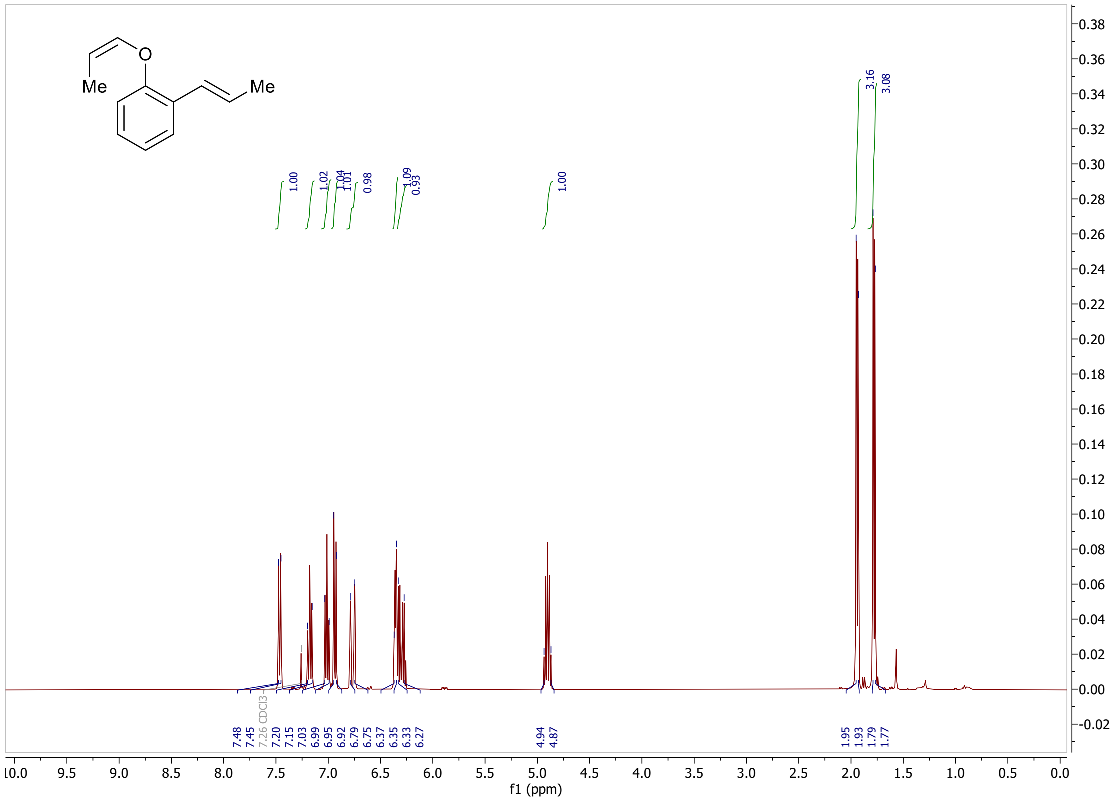

Figure S34. ^{13}C NMR spectrum of 1-((*E*)-prop-1-en-1-yl)-2-(((*E*)-prop-1-en-1-yl)oxy)benzene **(2q)** in $CDCl_3$

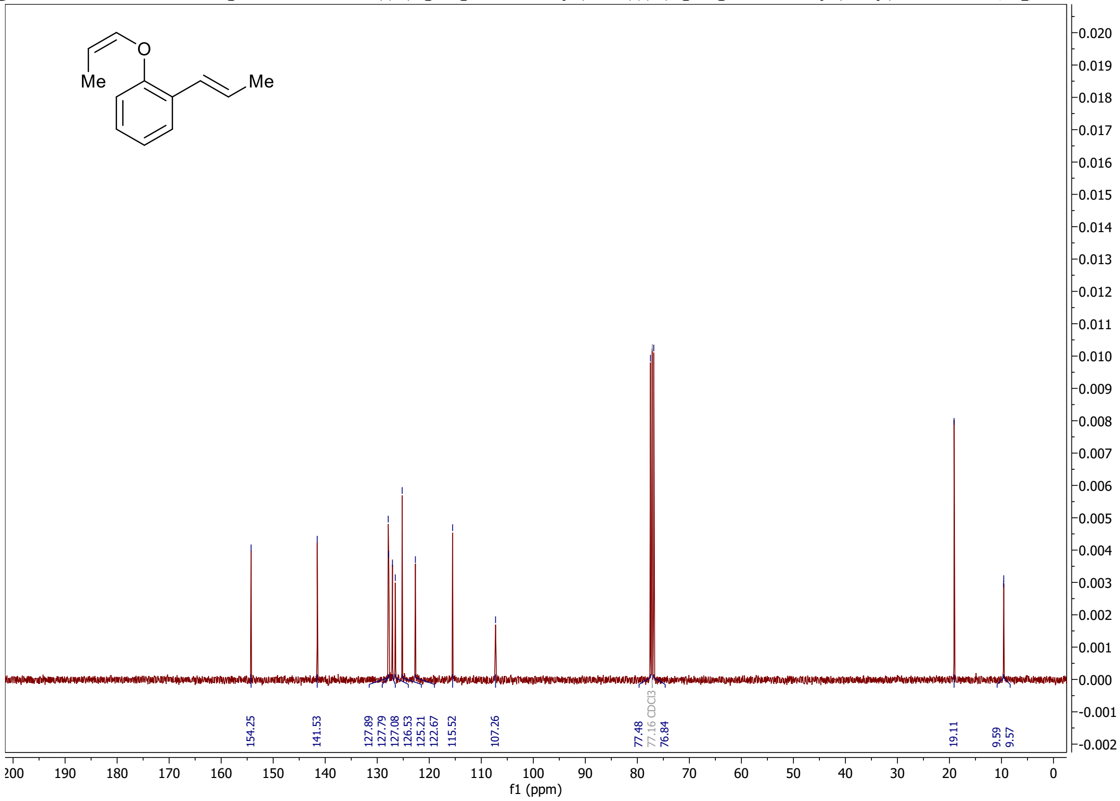

Figure S35. ^{1}H NMR spectrum of 2-methoxy-4-((*E*)-prop-1-en-1-yl)-1-(((*Z*)-prop-1-en-1-yl)oxy)benzene **(2r)** in $CDCl_3$

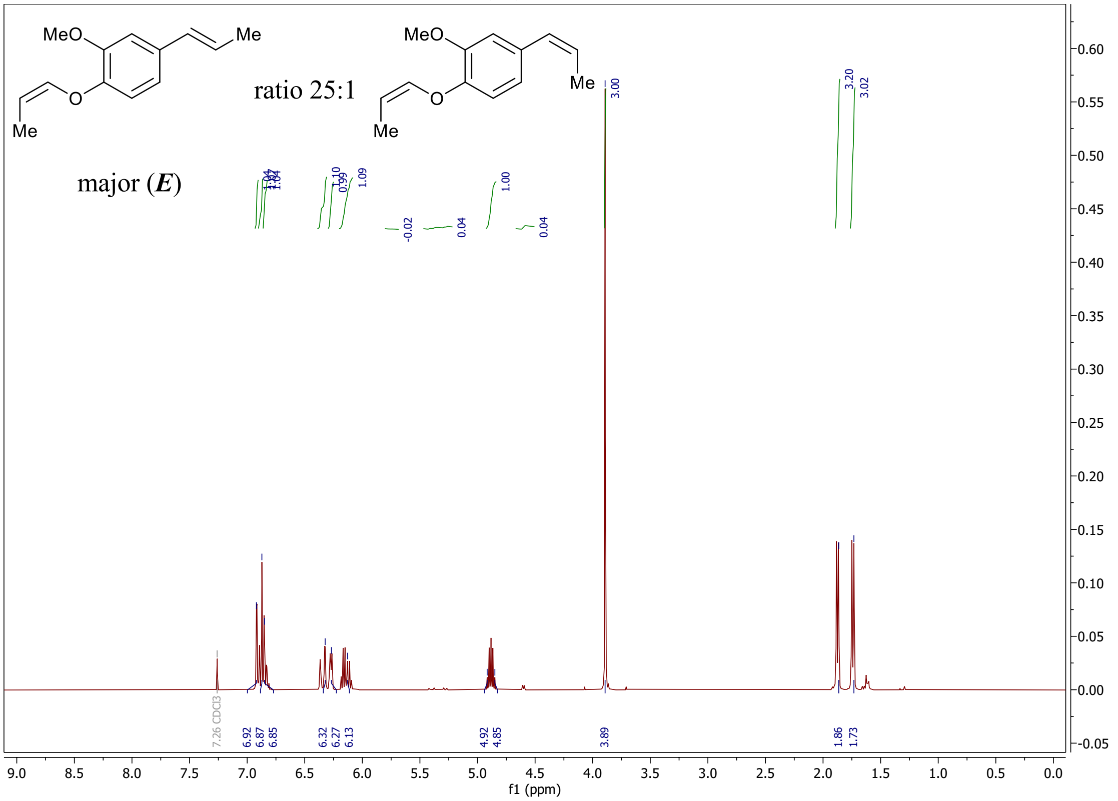

Figure S36. ^{13}C NMR spectrum of 2-methoxy-4-((*E*)-prop-1-en-1-yl)-1-(((*Z*)-prop-1-en-1-yl)oxy)benzene **(2r)** in $CDCl_3$

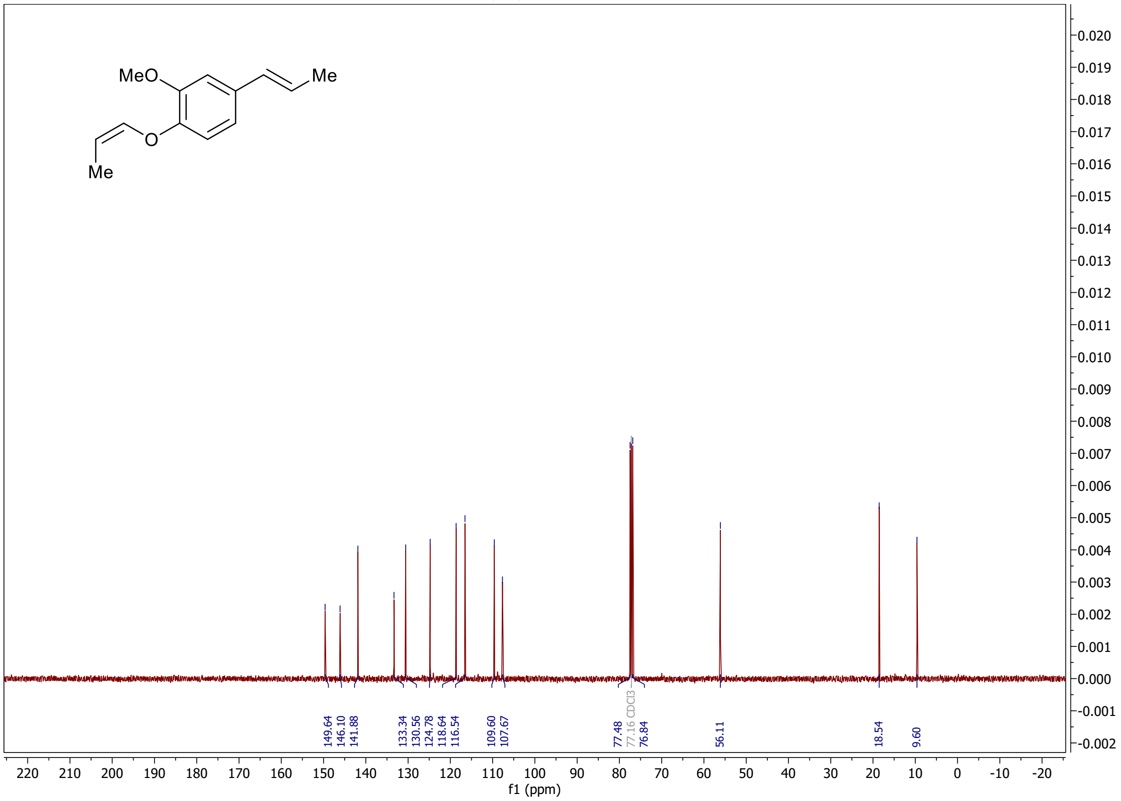

Figure S37. ^{1}H NMR spectrum of (*E*)-but-1-en-1-ylbenzene **(2s)** in $CDCl_3$

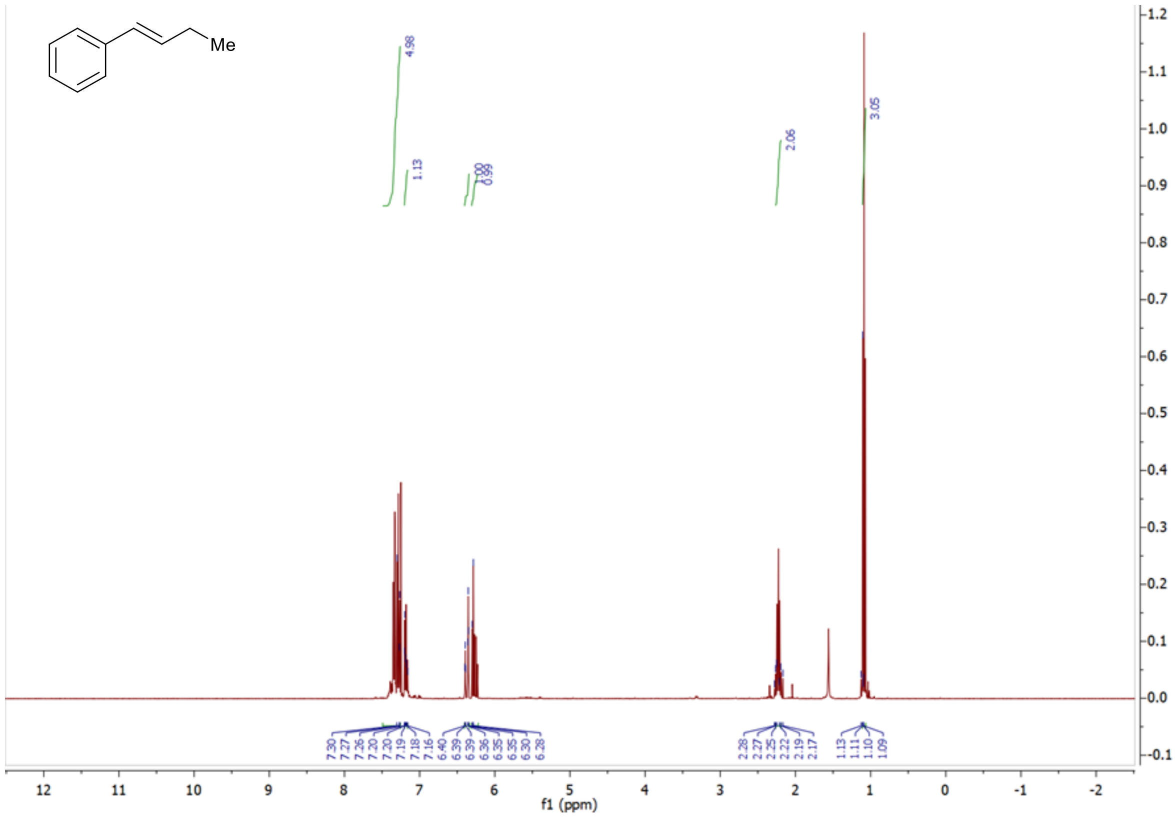

Figure S38. ^{13}C NMR spectrum of (*E*)-but-1-en-1-ylbenzene **(2s)** in $CDCl_3$

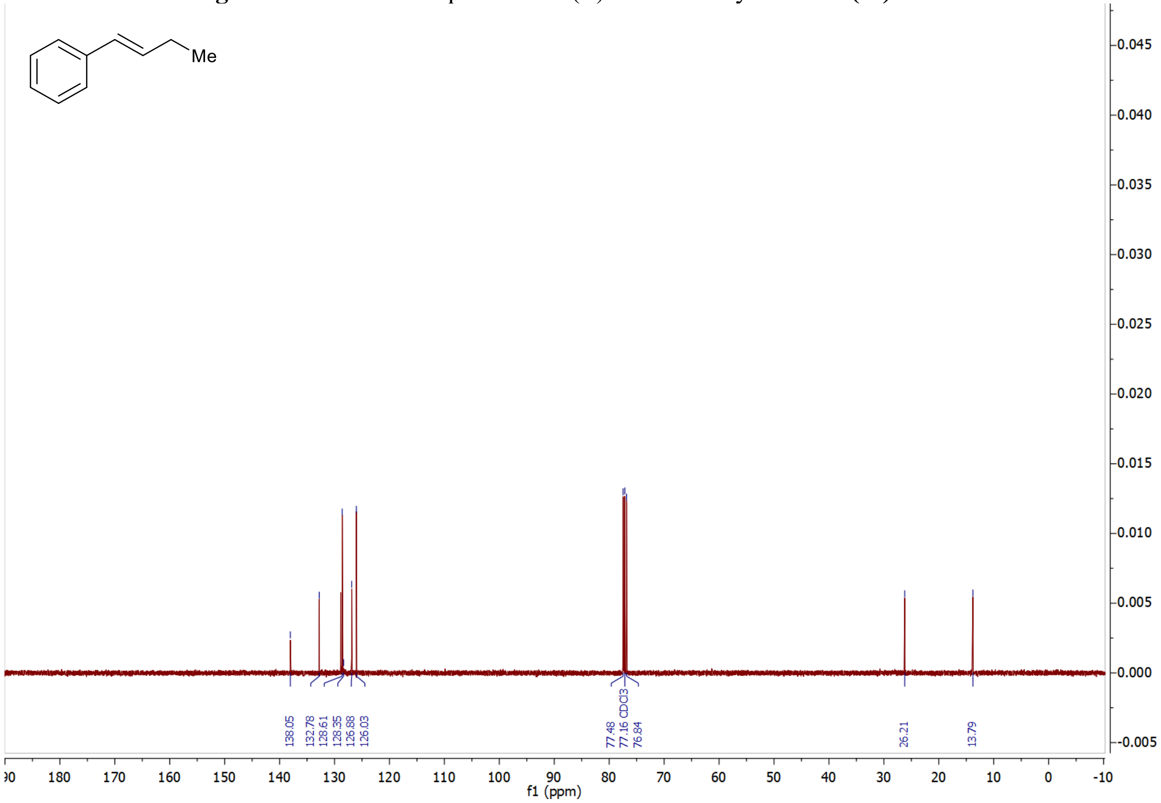

Figure S39. ^{1}H NMR spectrum of *p*-cymene **(2u)** in $CDCl_3$

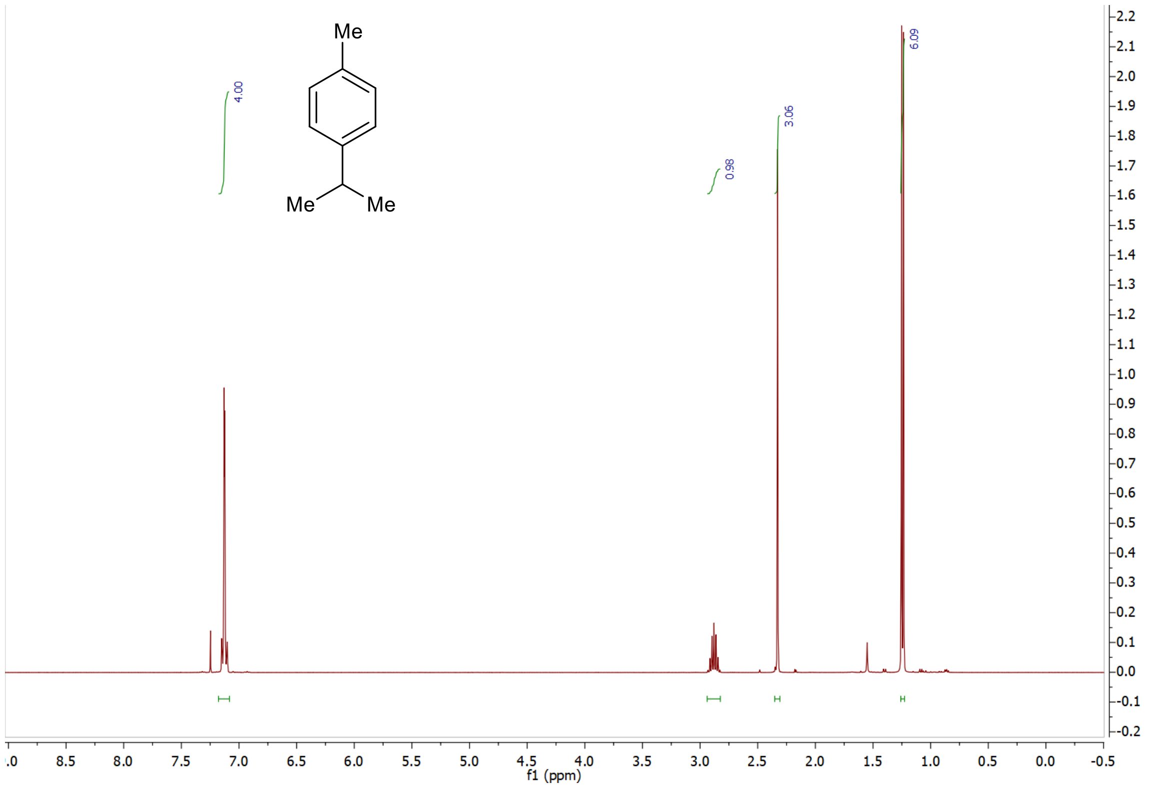

Figure S40. ^{13}C NMR spectrum of *p*-cymene **(2u)** in $CDCl_3$

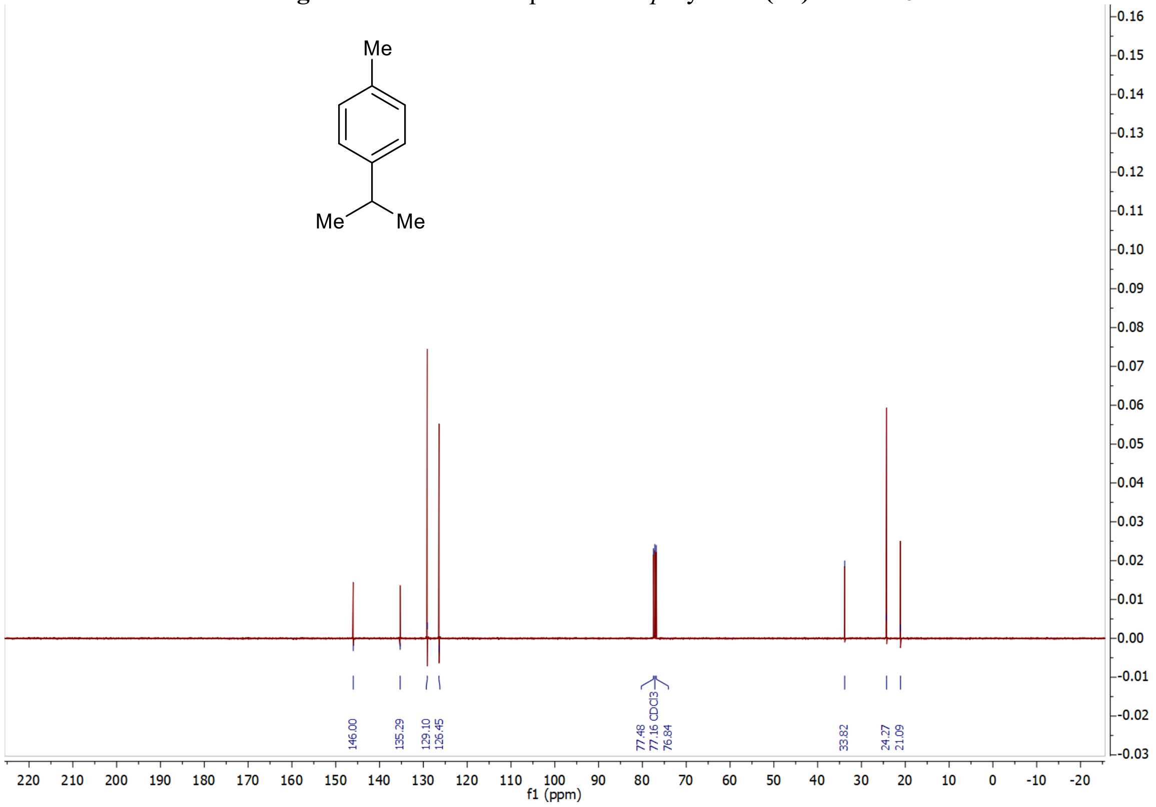

Figure S41. ^{1}H NMR spectrum of (1*Z*,3*Z*)-cycloocta-1,3-diene **(2v)** in $CDCl_3$

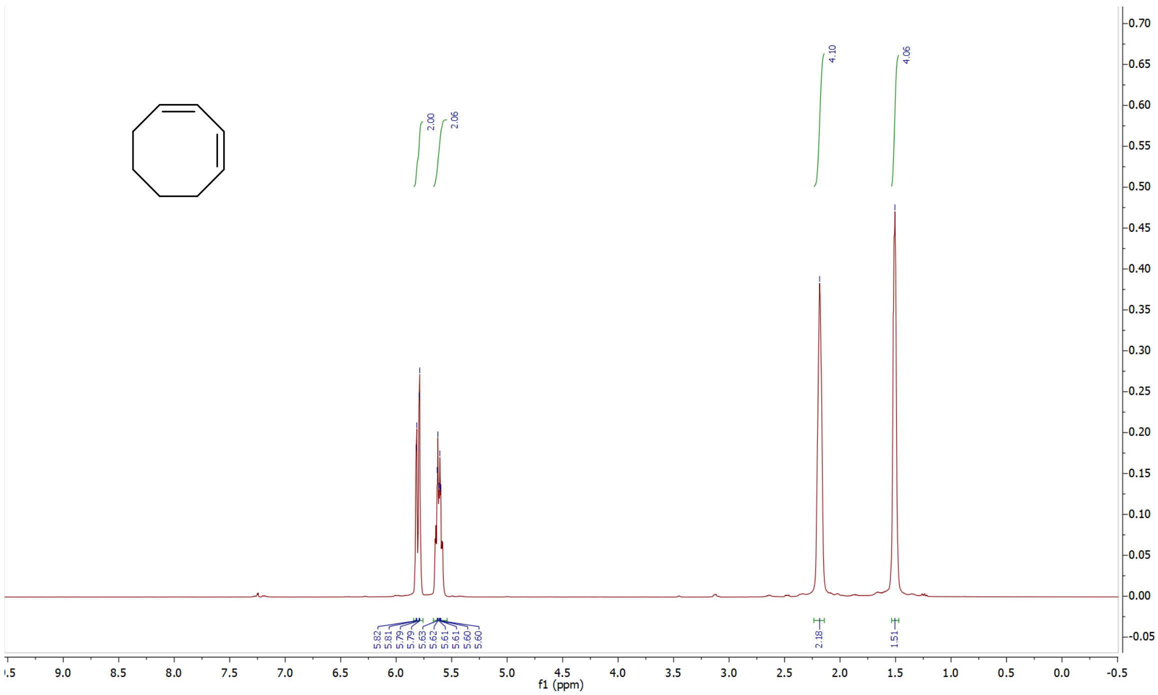

Figure S42. ^{13}C NMR spectrum of (1*Z*,3*Z*)-cycloocta-1,3-diene **(2v)** in $CDCl_3$

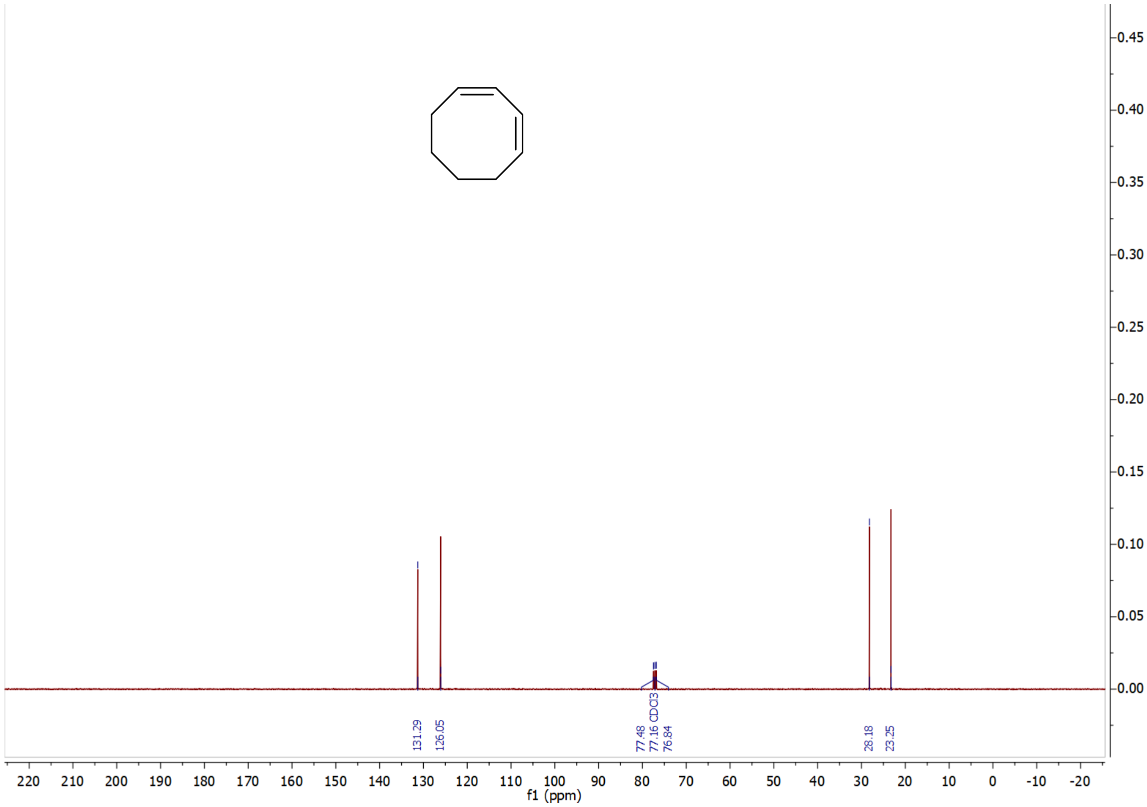

Figure S43. ^{1}H NMR spectrum of (*E*)-2-(prop-1-en-1-yl)phenol **(2z)** in $CDCl_3$

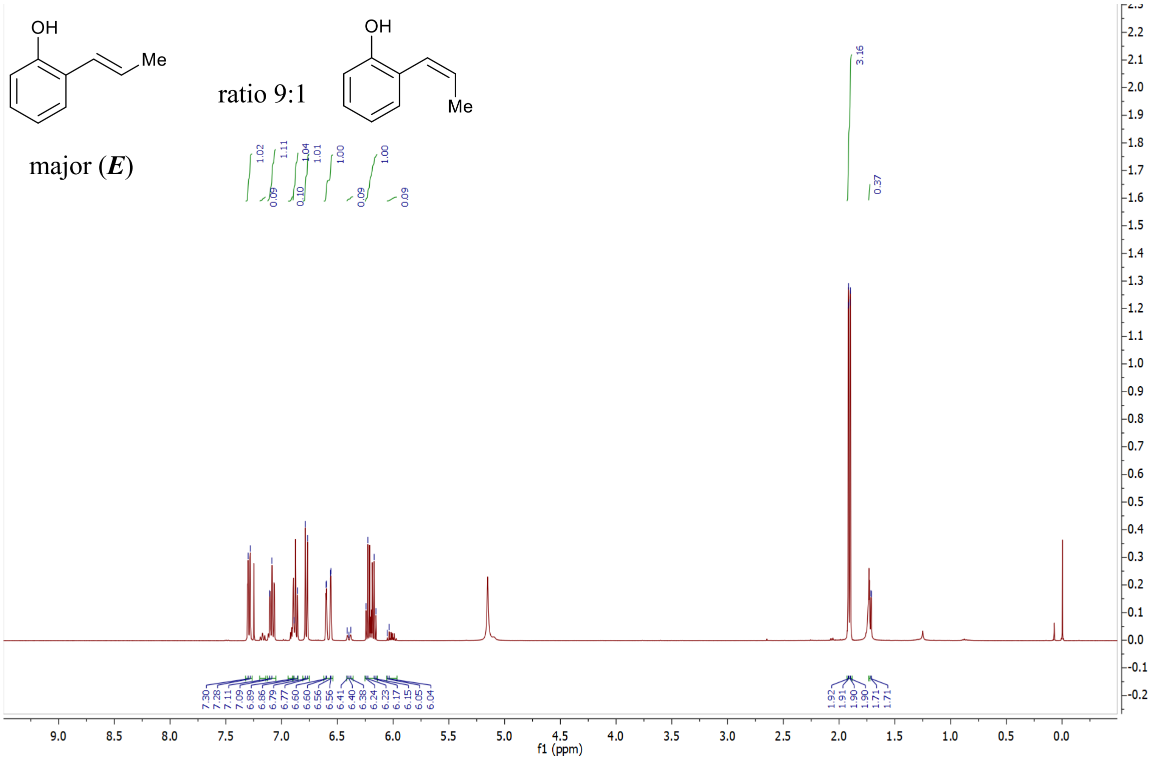

Figure S44. ^{13}C NMR spectrum of (*E*)-2-(prop-1-en-1-yl)phenol **(2z)** in $CDCl_3$

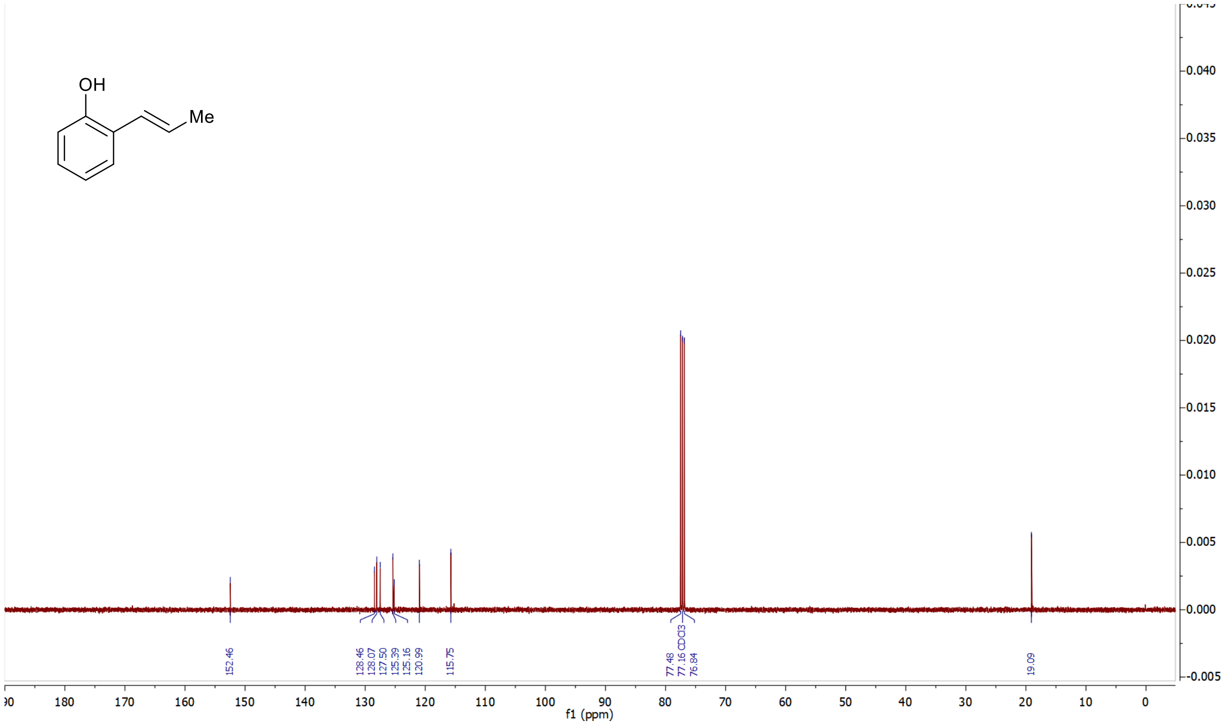

Figure S45. ^{1}H NMR spectrum of (*E*)-2-methoxy-4-(prop-1-en-1-yl)phenol benzene **(2ab)** in $CDCl_3$

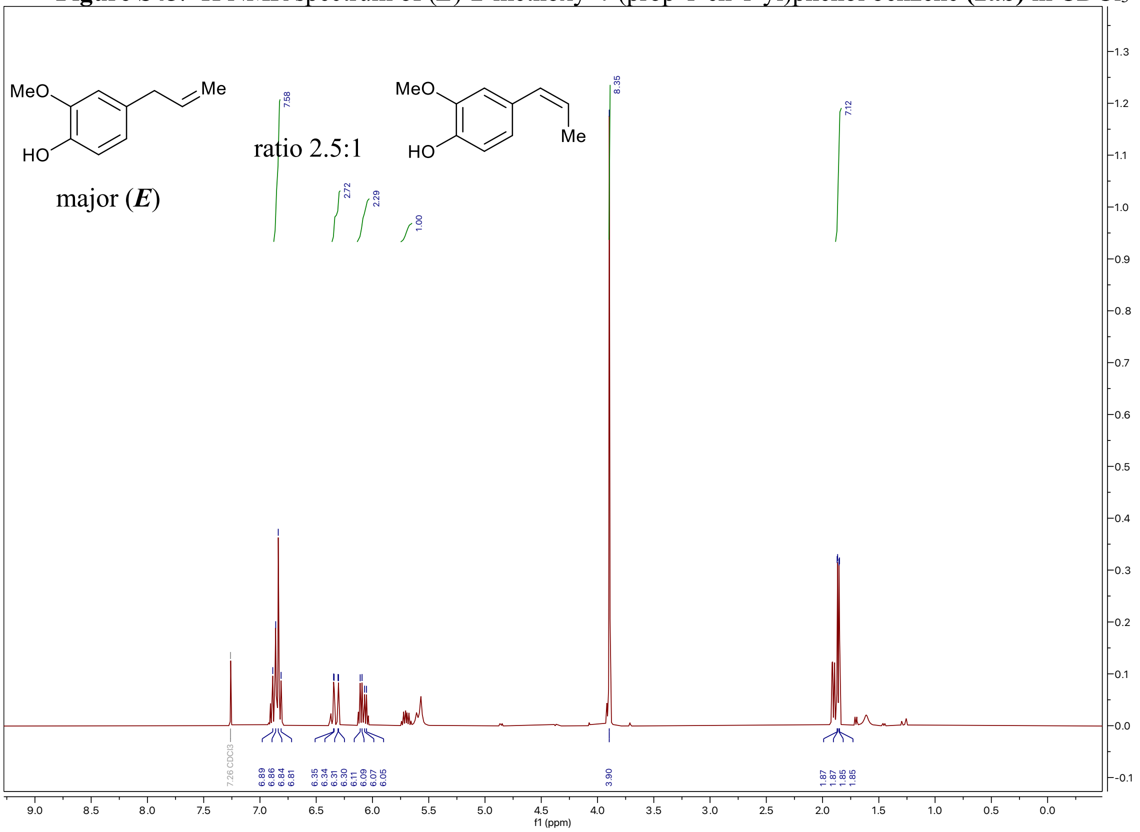

Figure S46. ^{13}C NMR spectrum of (*E*)-2-methoxy-4-(prop-1-en-1-yl)phenol benzene **(2ab)** in $CDCl_3$

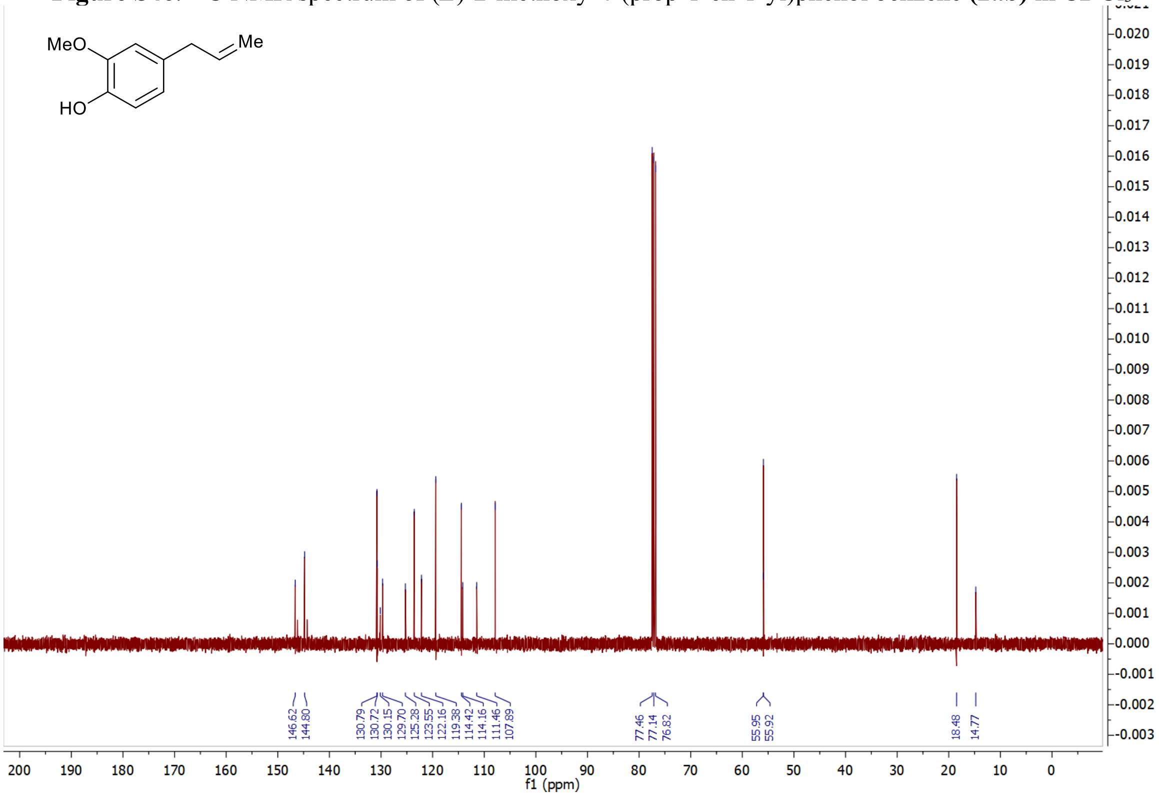

Figure S47. ^{1}H NMR spectrum of 1-phenylpropen-1-one **(2ac)** in $CDCl_3$

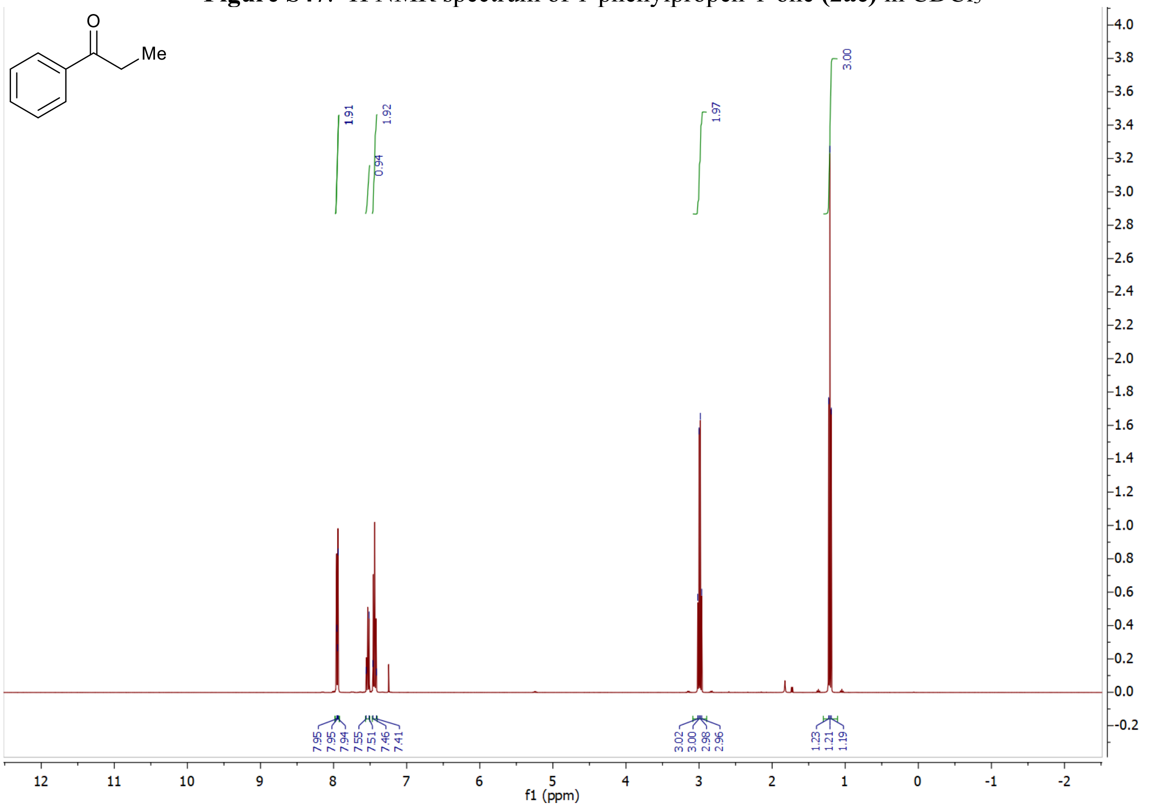

Figure S48. ^{13}C NMR spectrum of 1-phenylpropen-1-one **(2ac)** in $CDCl_3$

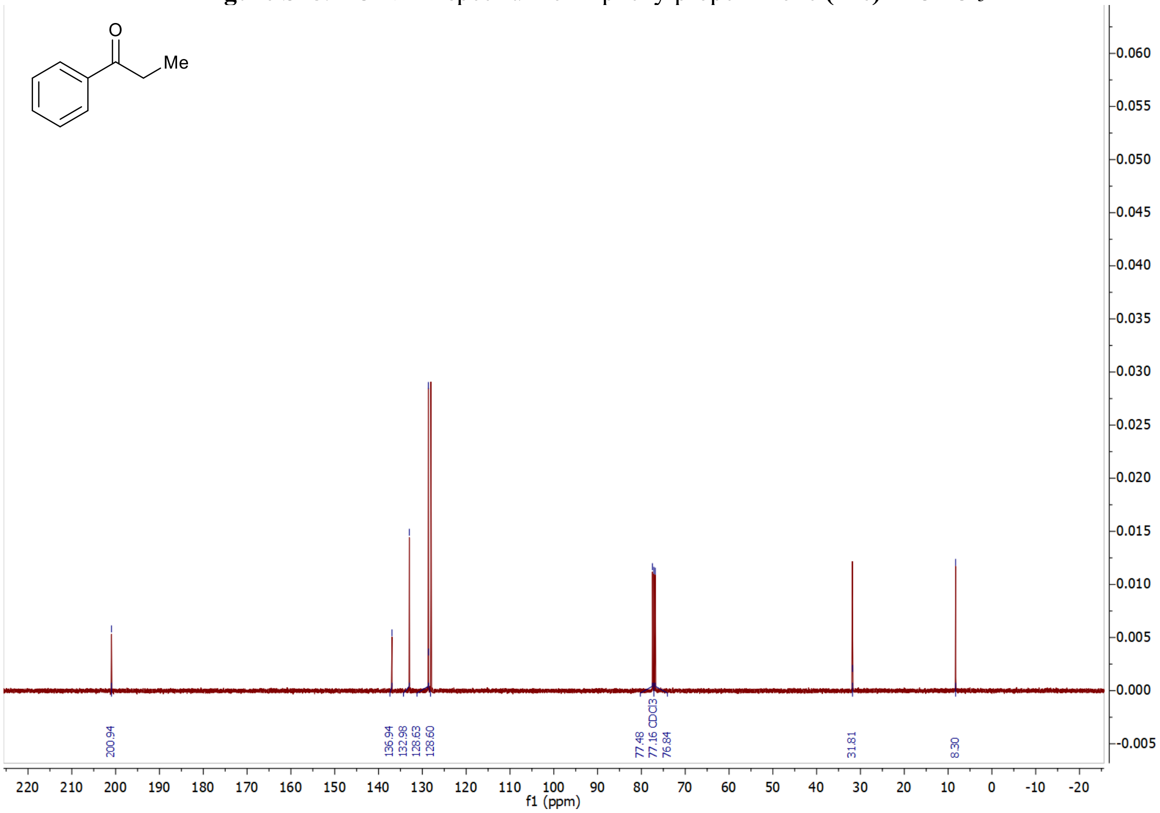

Figure S49. ¹H NMR spectrum of 1-phenylbutan-1-one **(2ad)** in $CDCl_3$

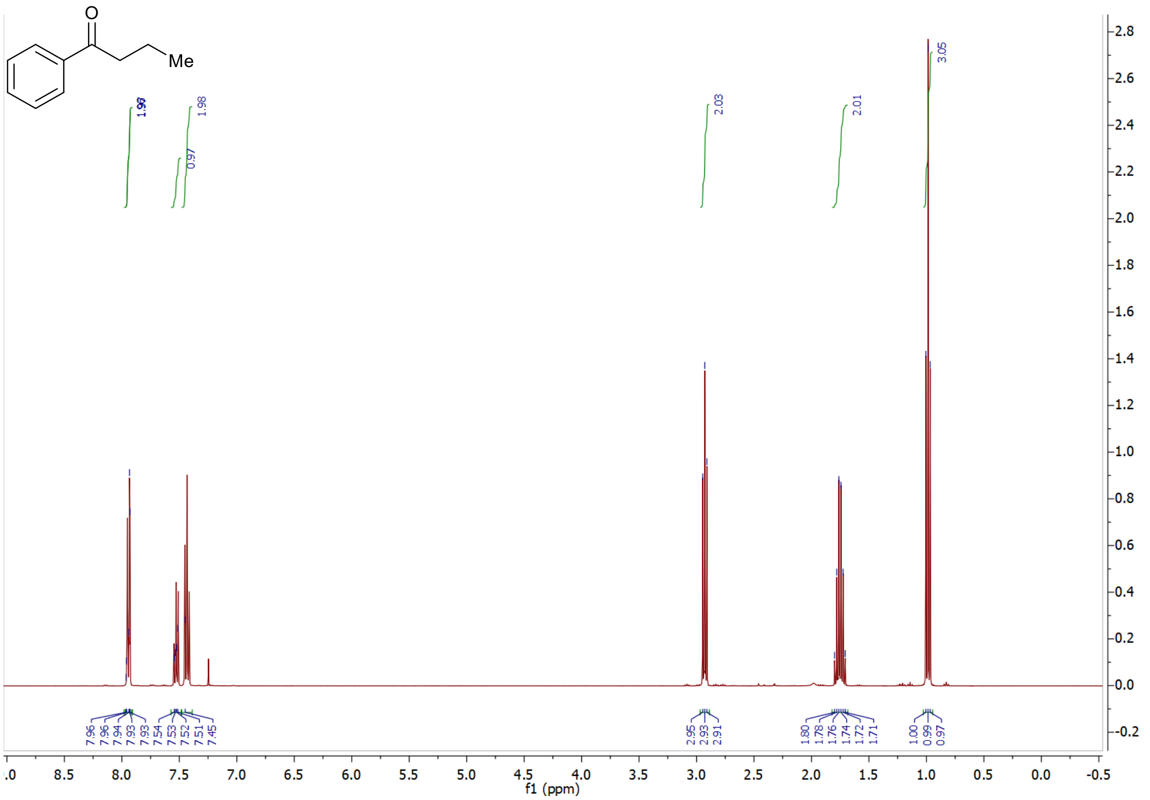

Figure S50. ¹³C NMR spectrum of 1-phenylbutan-1-one **(2ad)** in $CDCl_3$

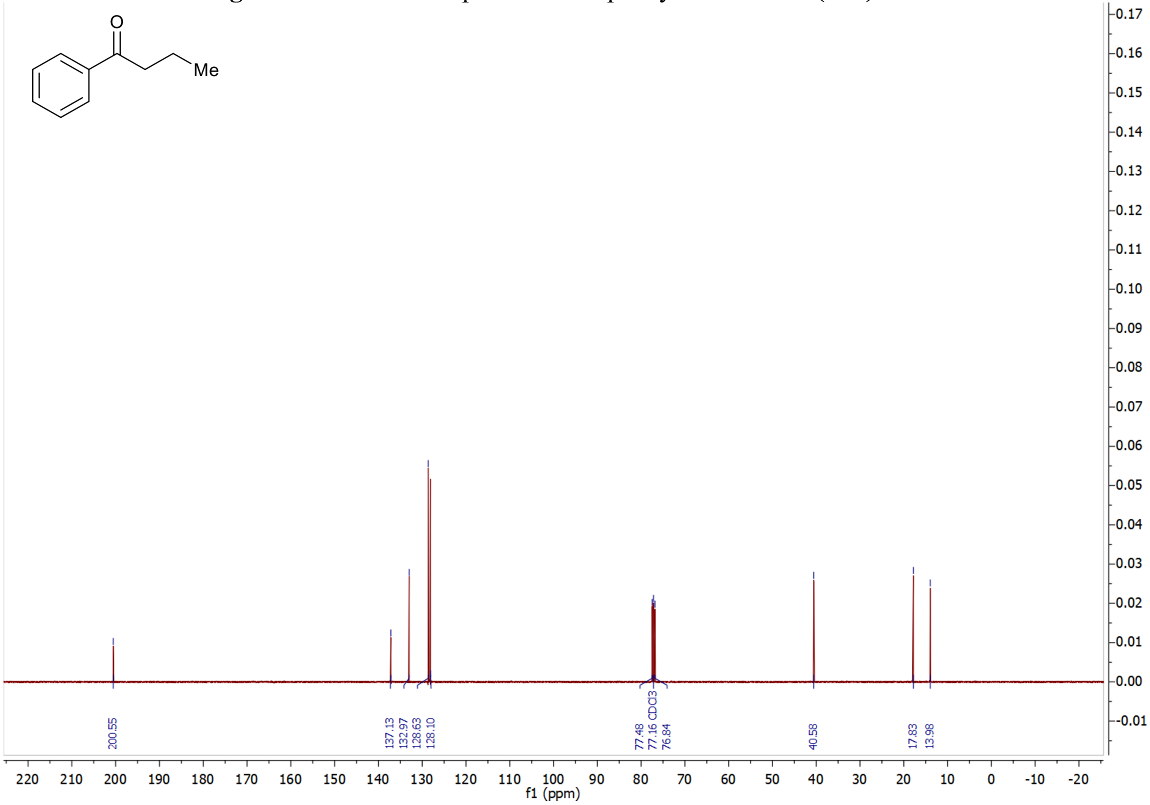

Figure S51. ^{1}H NMR spectrum of 4-nitrophenol **(2ae)** in DMSO-d_6

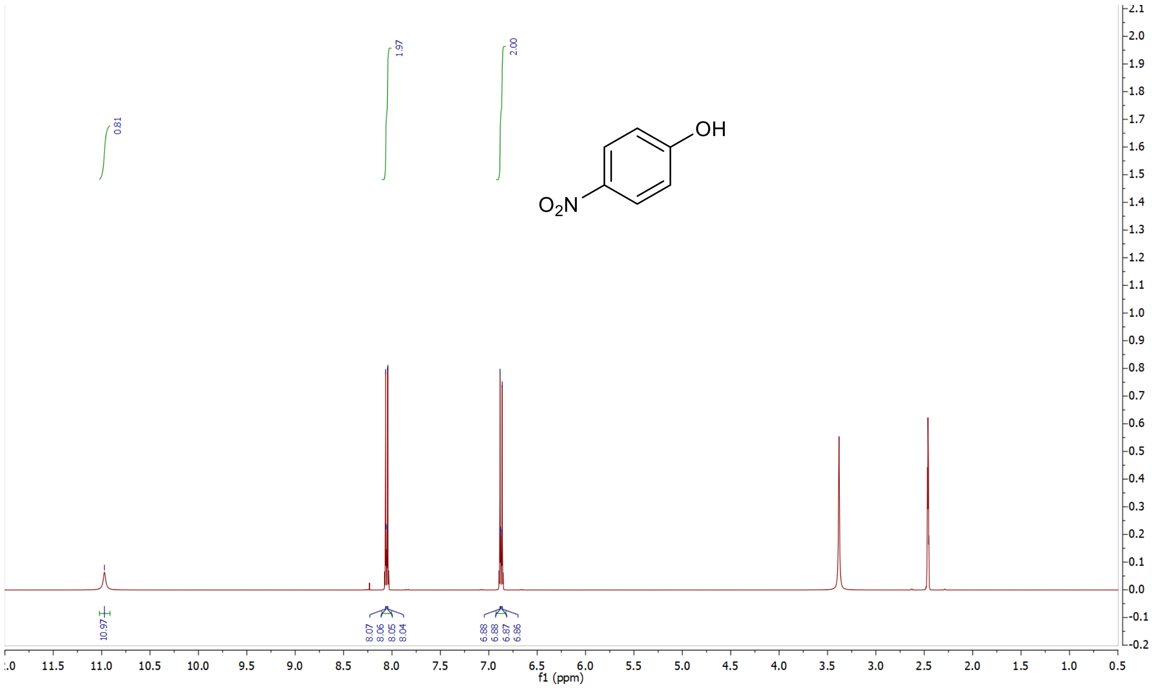

Figure S52. ^{13}C NMR spectrum of 4-nitrophenol **(2ae)** in DMSO-d_6

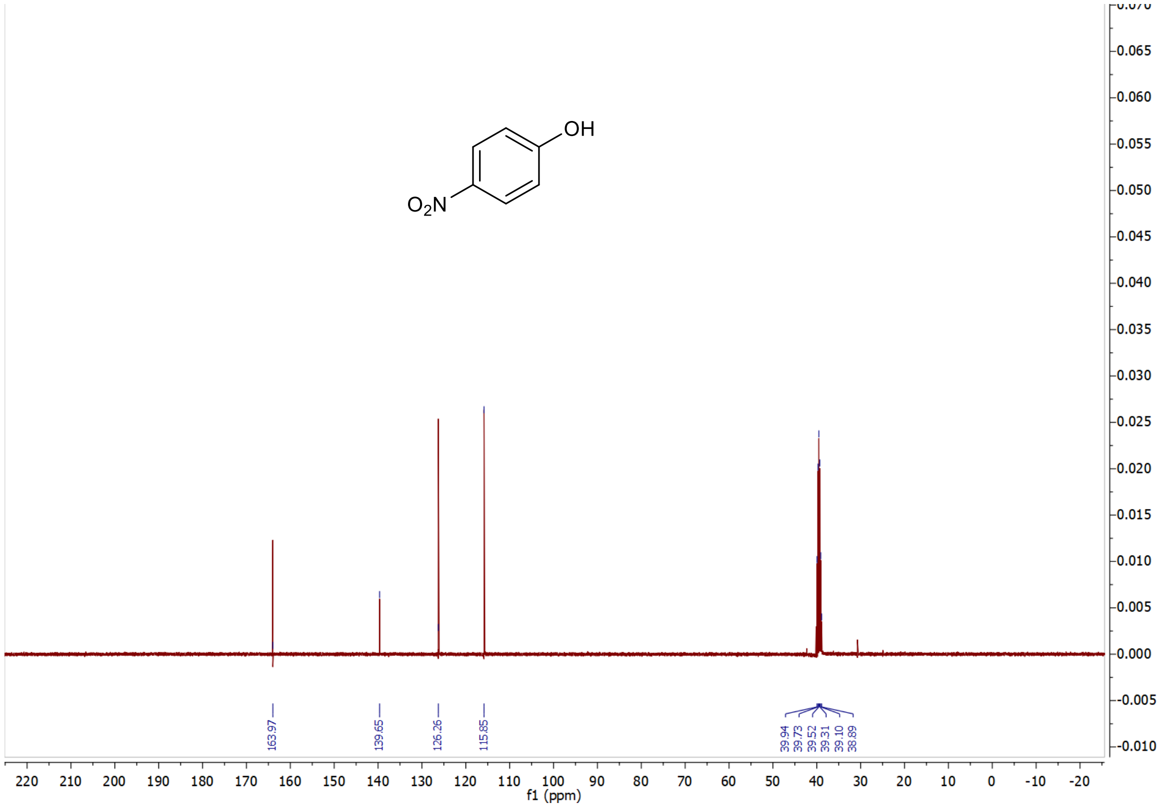

Figure S53. ^{1}H NMR spectrum of aniline **(2af)** in DMSO-d_6

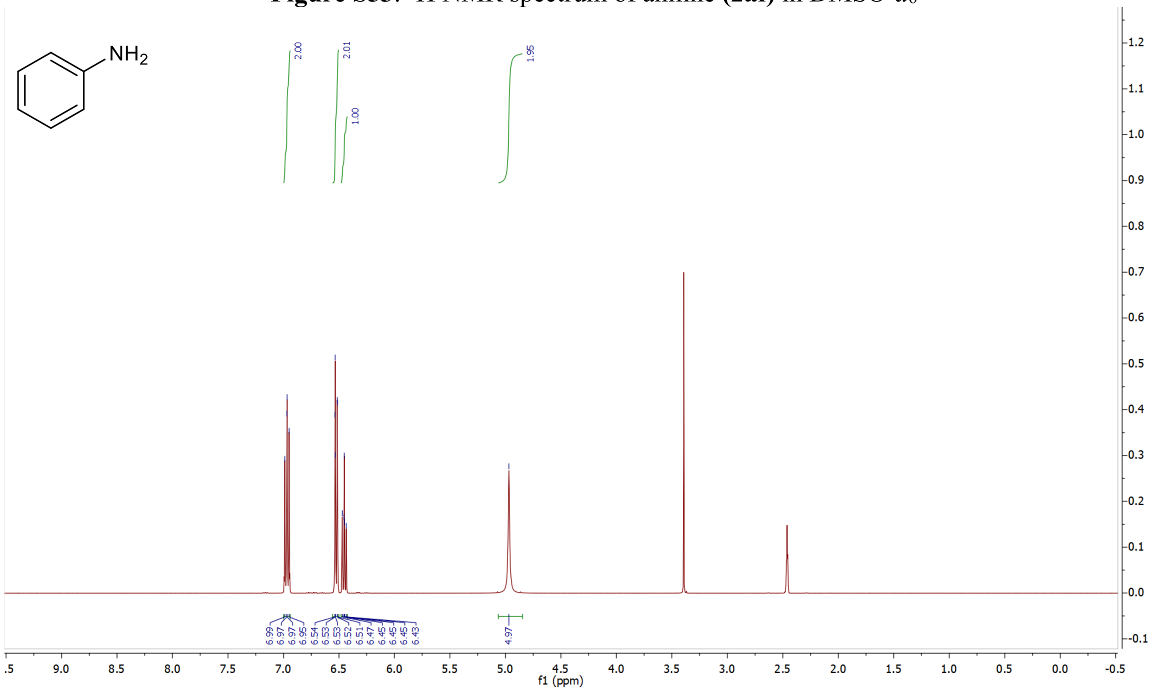

Figure S54. ^{13}C NMR spectrum of aniline **(2af)** in DMSO-d_6

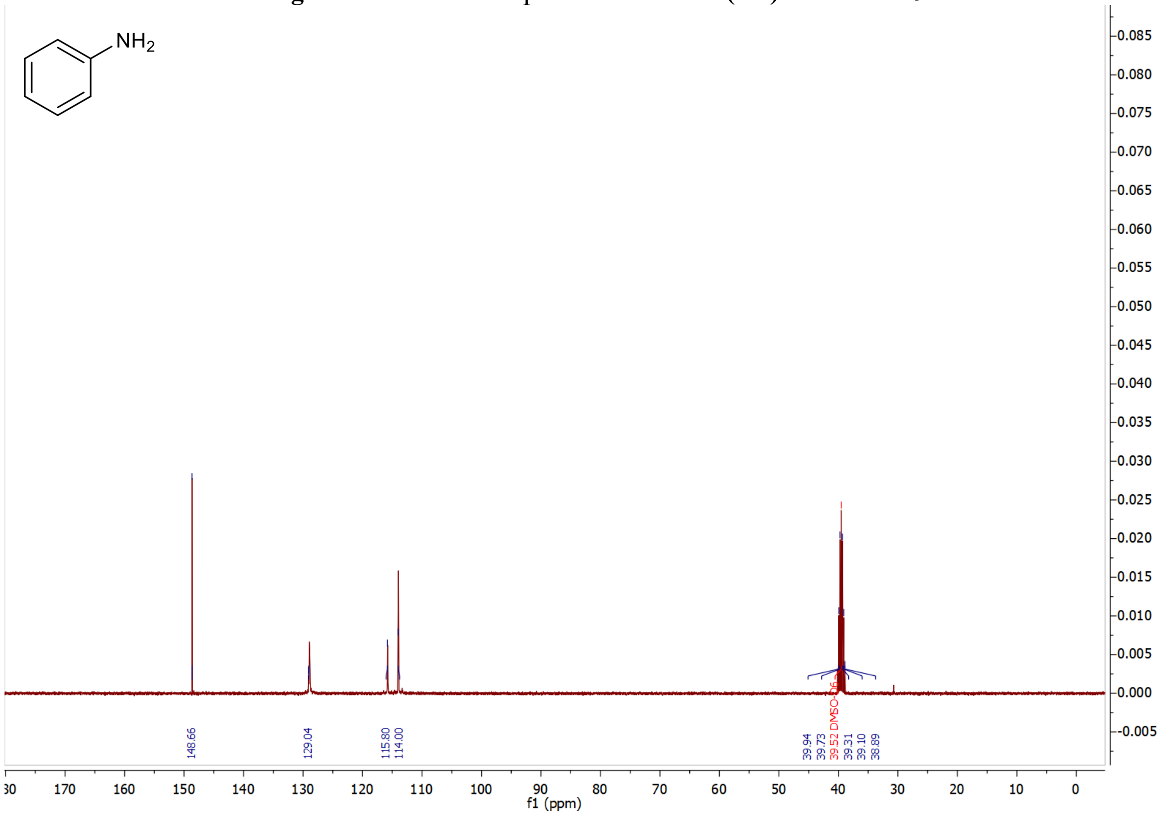

Figure S55. ^{1}H NMR spectrum of benzenesulfonic acid **(2ag)** in DMSO-d_6

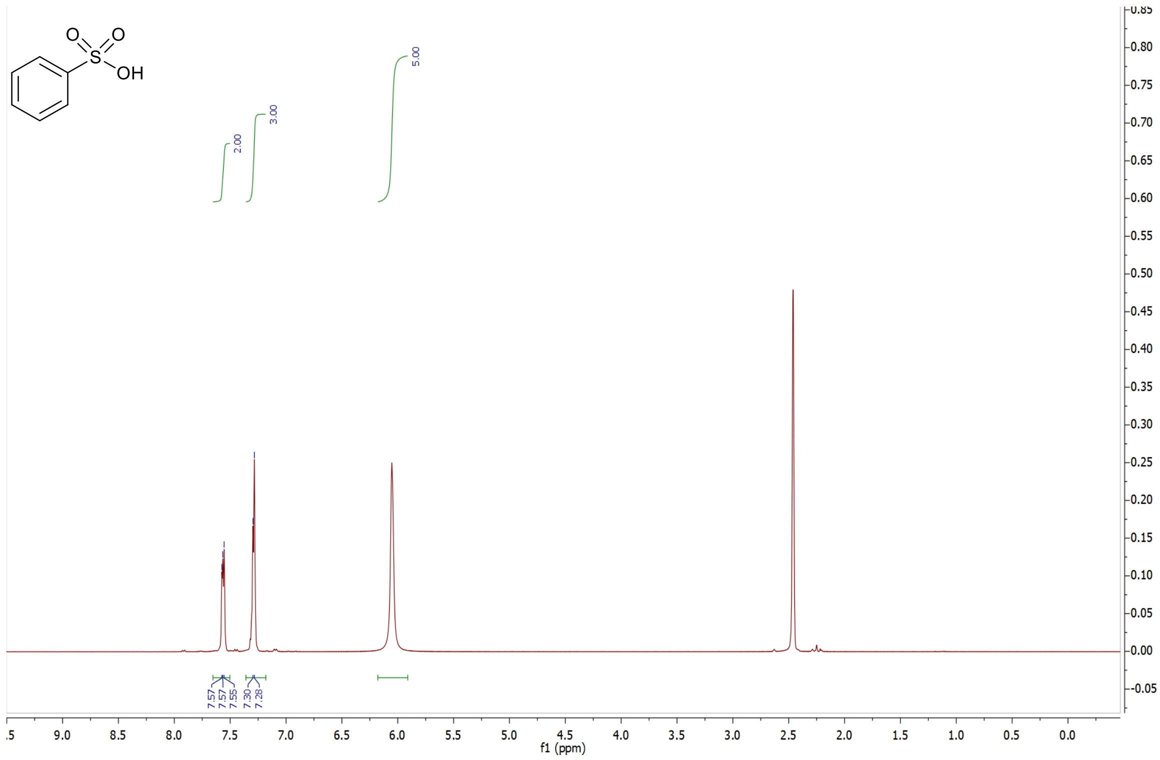

Figure S56. ^{13}C NMR spectrum of benzenesulfonic acid **(2ag)** in DMSO-d_6

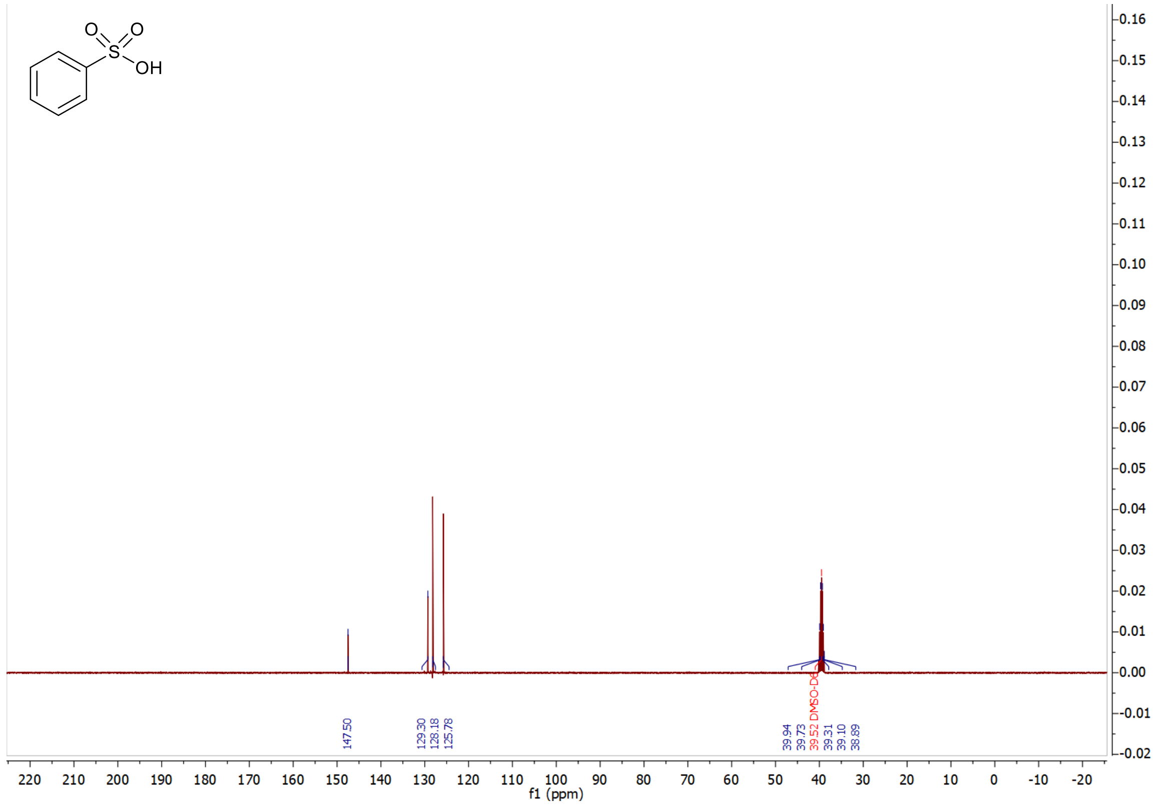

Figure S57. ^{1}H NMR spectrum of 4-methylbenzenesulfonic acid **(2ah)** in DMSO-d_6

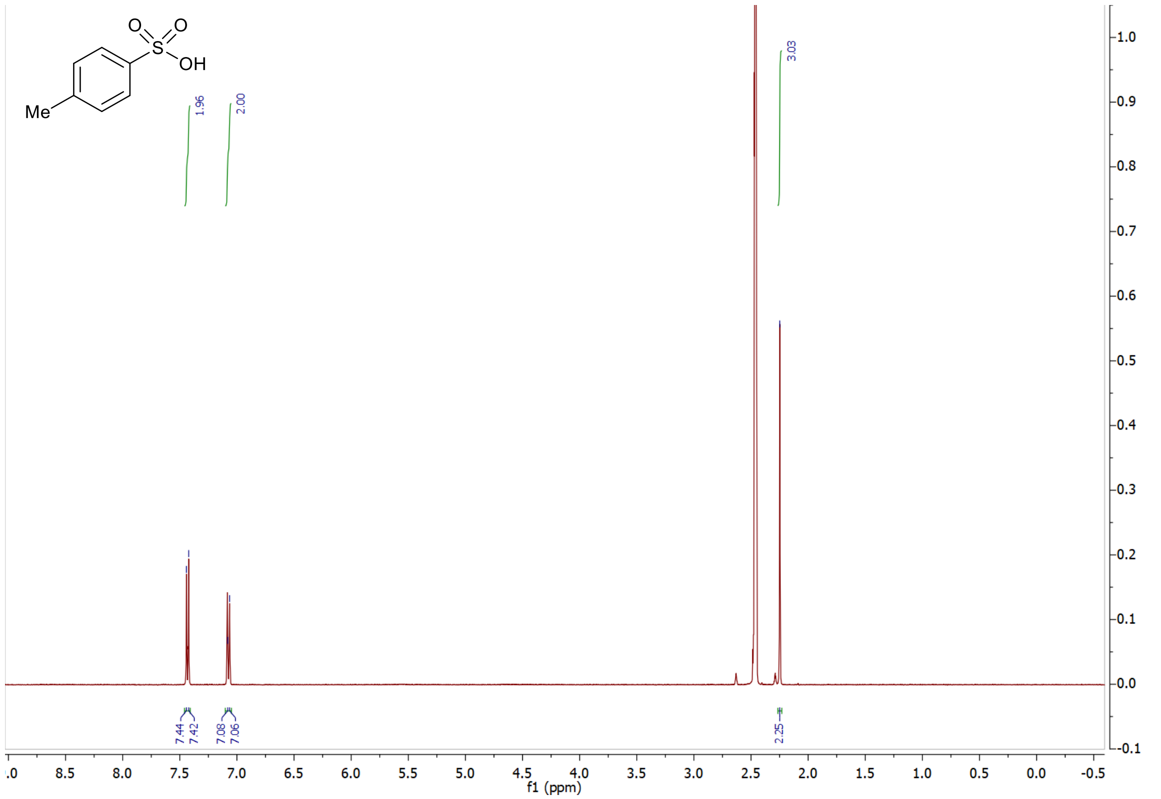

Figure S58. ^{13}C NMR spectrum of 4-methylbenzenesulfonic acid **(2ah)** in DMSO-d_6

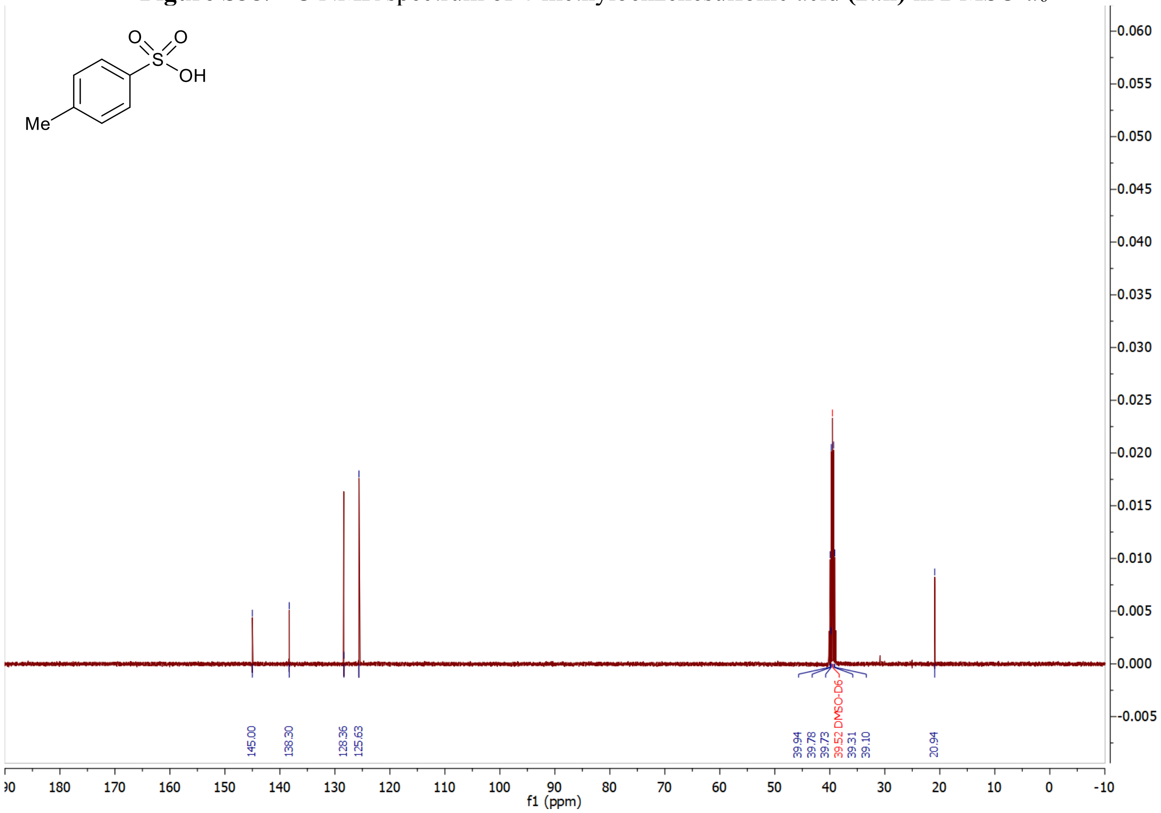

6. Extended optimization studies for selected substates

Table S3. Optimization of reaction conditions using allylbenzene[a]

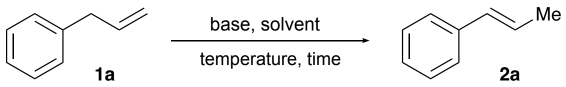

entry	alkene (1 equiv)	base (equiv)	solvent	Temp (°C)	time (min)	*E/Z* ratio	yield (%)[b]
1		none	DMSO	50 °C	120	N/A	NR
2		LiO^{t}Bu (0.25)	DMSO	rt	10	1:0	91
3		NaO^{t}Bu (0.25)	DMSO	rt	10	1:0	98
4		**KO^{t}Bu (0.25)**	**DMSO**	**rt**	**10**	**1:0**	**100**
5		KOH (0.25)	DMSO	rt	120	ND	traces
6		KOH (0.5)	DMSO	rt	120	30:1	93
7		KOH (0.75)	DMSO	rt	10	40:1	96
8		KOH (1.0)	DMSO	rt	10	50:1	98
9		Cs_2CO_3 (1.0)	DMSO	50 °C	120	N/A	NR
10		DBU (1.0)	DMSO	50 °C	120	N/A	NR
11		Et_3N (0.25)	DMSO	50 °C	120	1:0	10
12		Et_3N (1.0)	DMSO	50 °C	120	1:0	25
13		none	DMF	50 °C	120	N/A	NR
14		**KO^{t}Bu (0.25)**	**DMF**	**rt**	**10**	**108:1**	**100**
15		KO^{t}Bu (0.25)	MeCN	rt	10	1:0	23
16		KO^{t}Bu (1.0)	MeCN	50 °C	120	1:0	36
17		KO^{t}Bu (1.0)	DCM	50 °C	120	20:1	9
18		KO^{t}Bu (0.25)	Ethanol	50 °C	120	ND	3

[a]Reactions were carried out with allylbenzene **1a** (0.4 mmol, 53 μL, 1 equiv.), Base (0.1 mmol, 0.25 equiv. or 0.4 mmol, 1.0 equiv.), in 1.0 mL of solvent at room temperature (~22 °C) or at 50 °C. Then, the mixture was stirred for 10 minutes, [b]the yield was analyzed by ^{1}H NMR using DMS0-d_6 as the solvent, and mesitylene as the internal standard. Those unreacted were analyzed again at 2 h. NR = no reaction, ND = no determined, N/A = not applicable.

Table S4. Optimization of reaction conditions using allyl phenyl sulfide[a]

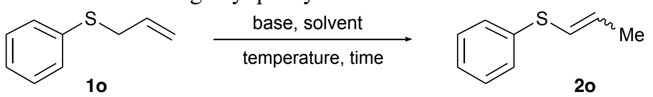

entry	alkene (1 equiv)	base (equiv)	solvent	Temp (°C)	time (min)	*E/Z* ratio	yield (%)[b]
1		none	DMSO	50 °C	120	N/A	NR
2		LiO*t*Bu (0.25)	DMSO	rt	10	1.5:1	100
3		NaO*t*Bu (0.25)	DMSO	rt	10	1:1	100
4		**KO*t*Bu (0.25)**	**DMSO**	**rt**	**10**	**1:1**	**100**
5		KOH (0.25)	DMSO	rt	10	1:1.5	35
6		KOH (1.0)	DMSO	50 °C	120	1:1.2	100
7		Cs_2CO_3 (1.0)	DMSO	50 °C	120	N/A	NR
8		DBU (1.0)	DMSO	50 °C	120	N/A	NR
9		Et_3N (1.0)	DMSO	50 °C	120	1:1	20
10		none	DMF	50 °C	120	N/A	NR
11		**KO*t*Bu (0.25)**	**DMF**	**rt**	**10**	**1:1**	**100**
12		KO*t*Bu (0.25)	MeCN	rt	10	1.5:1	40
13		KO*t*Bu (0.25)	MeCN	50 °C	120	1.3:1	57
14		KO*t*Bu (0.25)	DCM	rt	10	1:1	33
15		KO*t*Bu (0.25)	DCM	50 °C	120	1:1	72
16		KO*t*Bu (1.0)	Ethanol	50 °C	120	ND	3

[a]Reactions were carried out with allyl phenyl sulfide **1o** (0.4 mmol, 60.1 mg, 1 equiv.), Base (0.1 mmol, 0.25 equiv. or 0.4 mmol, 1.0 equiv.), in 1.0 mL of solvent at room temperature (~22 °C) or at 50 °C. Then, the mixture was stirred for 10 minutes, [b] the yield was analyzed by ^{1}H NMR using DMS0-d_6 as the solvent, and mesitylene as the internal standard. Those unreacted were analyzed again at 2 h. NR = no reaction, ND = not determined, N/A = not applicable.

Table S5. Optimization of reaction conditions using allyl phenyl ether[a]

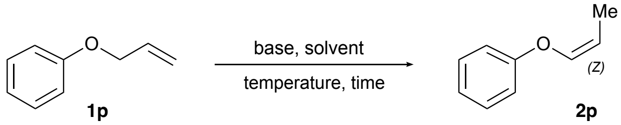

entry	alkene (1 equiv)	base (equiv)	solvent	Temp (°C)	time (min)	*E/Z* ratio	yield (%)[b]
1		none	DMSO	50 °C	120	N/A	NR
2		LiO*t*Bu (0.25)	DMSO	rt	10	0:1	5
3		NaO*t*Bu (0.25)	DMSO	rt	10	0:1	67
4		**KO*t*Bu (0.25)**	**DMSO**	**rt**	**10**	**0:1**	**100**
5		KOH (1.0)	DMSO	rt	10	N/A	NR
6		KOH (1.0)	DMSO	50 °C	120	0:1	42
7		Cs_2CO_3 (1.0)	DMSO	50 °C	120	N/A	NR
8		DBU (1.0)	DMSO	50 °C	120	N/A	NR
9		Et_3N (1.0)	DMSO	50 °C	120	N/A	NR
10		none	DMF	50 °C	120	N/A	NR
11		**KO*t*Bu (0.25)**	**DMF**	**rt**	**10**	0:1	**76**
12		KO*t*Bu (1.0)	MeCN	50 °C	120	N/A	NR
13		KO*t*Bu (1.0)	DCM	50 °C	120	N/A	NR
14		KO*t*Bu (1.0)	Ethanol	50 °C	120	N/A	NR

[a]Reactions were carried out with allyl phenyl ether **1p** (0.4 mmol, 53.7 mg, 1 equiv.), Base (0.1 mmol, 0.25 equiv. or 0.4 mmol, 1.0 equiv.), in 1.0 mL of solvent at room temperature (~22 °C) or at 50 °C. Then, the mixture was stirred for 10 minutes, [b] the yield was analyzed by ^{1}H NMR using DMS0-d_6 as the solvent, and mesitylene as the internal standard. Those unreacted were analyzed again at 2 h. NR = no reaction, ND = no determined, N/A = not applicable.

Table S6. Optimization of reaction conditions using 4-phenyl-1-butene[a]

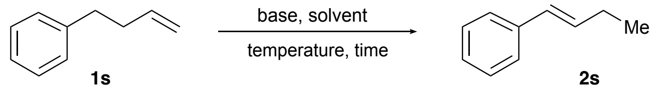

entry	alkene (1 equiv)	base (equiv)	solvent	Temp (°C)	time (min)	*E/Z* ratio	yield (%)[b]
1		none	DMSO	50 °C	120	N/A	NR
2		LiO*t*Bu (1.0)	DMSO	50 °C	120	ND	traces
3		NaO*t*Bu (1.0)	DMSO	50 °C	120	ND	traces
4		KO*t*Bu (1.0)	DMSO	rt	10	1/0	20
5		**KO*t*Bu (1.0)**	**DMSO**	**50 °C**	**120**	**1/0**	**100**[c]
6		KOH (1.0)	DMSO	rt	10	N/A	NR
7		KOH (1.0)	DMSO	50 °C	120	N/A	NR
8		Cs_2CO_3 (1.0)	DMSO	50 °C	120	N/A	NR
9		DBU (1.0)	DMSO	50 °C	120	N/A	NR
10		Et_3N (1.0)	DMSO	50 °C	120	N/A	NR
11		none	DMF	50 °C	120	N/A	NR
12		KO*t*Bu (1.0)	DMF	rt	10	N/A	NR
13		KO*t*Bu (1.0)	DMF	50 °C	120	ND	traces
14		KO*t*Bu (1.0)	MeCN	50 °C	120	N/A	NR
15		KO*t*Bu (1.0)	DCM	50 °C	120	N/A	NR
16		KO*t*Bu (1.0)	Ethanol	50 °C	120	N/A	NR

[a]Reactions were carried out with 4-phenyl-1-butene **1s** (0.4 mmol, 52.9 mg, 1.0 equiv.), Base (0.4 mmol, 1.0 equiv.), in 1.0 mL of solvent at room temperature (~22 °C) or at 50 °C. Then, the mixture was stirred for 10 minutes, [b]the yield was analyzed by ^1H NMR using DMS0-d_6 as the solvent, and mesitylene as the internal standard. Those unreacted were analyzed again at 2 h. NR = no reaction, ND = no determined, N/A = not applicable. , [c]the compound is volatile.

Table S7. Optimization of reaction conditions using *N*-allyl aniline[a]

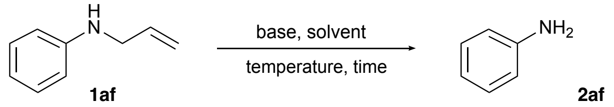

entry	alkene (1 equiv)	base (equiv)	solvent	Temp (°C)	time (min)	*E/Z* ratio	yield (%)[b]
1		none	DMSO	50 °C	120	N/A	NR
2		LiO*t*Bu (1.0)	DMSO	50 °C	120	N/A	7
3		NaO*t*Bu (1.0)	DMSO	50 °C	120	N/A	15
4		KO*t*Bu (1.0)	DMSO	rt	10	N/A	50
5		**KO*t*Bu (1.0)**	**DMSO**	**50 °C**	**120**	**N/A**	**100**
6		KOH (1.0)	DMSO	rt	10	N/A	0
7		KOH (1.0)	DMSO	50 °C	120	N/A	18
8		Cs_2CO_3 (1.0)	DMSO	50 °C	120	N/A	NR
9		DBU (1.0)	DMSO	50 °C	120	N/A	NR
10		Et_3N (1.0)	DMSO	50 °C	120	N/A	NR
11		none	DMF	50 °C	120	N/A	NR
12		KO*t*Bu (1.0)	DMF	rt	10	N/A	20
13		KO*t*Bu (1.0)	DMF	50 °C	120	N/A	33
14		KO*t*Bu (1.0)	MeCN	50 °C	120	N/A	NR
15		KO*t*Bu (1.0)	DCM	50 °C	120	N/A	traces
16		KO*t*Bu (1.0)	Ethanol	50 °C	120	N/A	NR

[a]Reactions were carried out with *N*-allylaniline **1af** (0.4 mmol, 53.3 mg, 1 equiv.), Base (0.4 mmol, 1.0 equiv.), in 1.0 mL of solvent at room temperature (~22 °C) or at 50 °C. Then, the mixture was stirred for 10 minutes, [b]the yield was analyzed by ^1H NMR using DMS0-d_6 as the solvent, and mesitylene as the internal standard. Those unreacted were analyzed again at 2 h. NR = no reaction, ND = no determined, N/A = not applicable.
